# Supplementary material for: Maternal gut and breast milk microbiota affect infant gut antibiotic resistome and mobile genetic elements
Source: Nat Commun. 2018 Sep 24;9:3891. doi: 10.1038/s41467-018-06393-w (PMC6155145; doi:10.1038/s41467-018-06393-w)
Supplement: Supplementary file 1 — Supplementary Information [file 41467_2018_6393_MOESM1_ESM.pdf]

Supplementary Information

Maternal gut and breast milk microbiota affect infant gut antibiotic resistome and mobile genetic elements

Pärnänen et al.

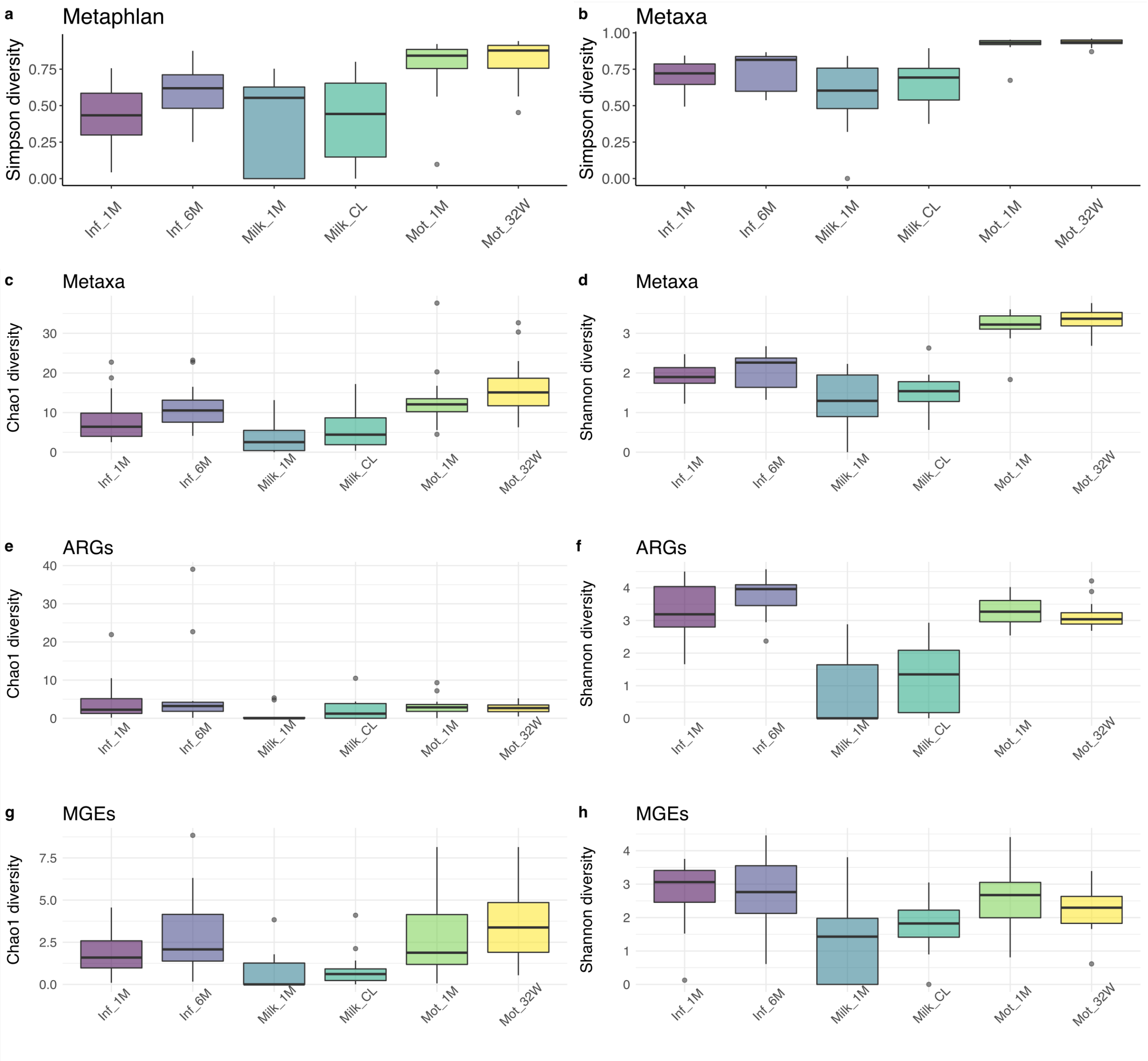

Supplementary Figure 1: Simpson and Shannon diversity and Chao1 estimate of richness
a, Microbial taxa diversity with Simpson index using Metaphlan2[1] taxonomic profiling **b**, Microbial taxa diversity with Simpson index using Metaxa2[2] taxonomic profiling **c**, Microbial taxa richness estimate with Chao1 index using Metaxa2[2] taxonomic profiling **d**, Microbial taxa diversity with Shannon index using Metaxa2[2] taxonomic profiling. **e**, ARG richness estimate with Chao1 index **f**, ARG diversity with Shannon index **g**, MGE richness estimate with Chao1 index **h**, MGE diversity with Shannon index. In boxplots the lower hinge represents 25% quantile, upper hinge 75% quantile and center line the median. Notches are calculated with the formula median +- 1.58 * interquartile range / sqrt(n). Sample names are as follows: Inf_1M = one-month-old infants, Inf_6M = six-month-old infants, Mot_32W = mother fecal samples gestational week 32, Mot_1M = mother fecal samples one month postpartum, Milk_CL = colostrum or milk produced within seven days after delivery, Milk_1M = milk one month postpartum.

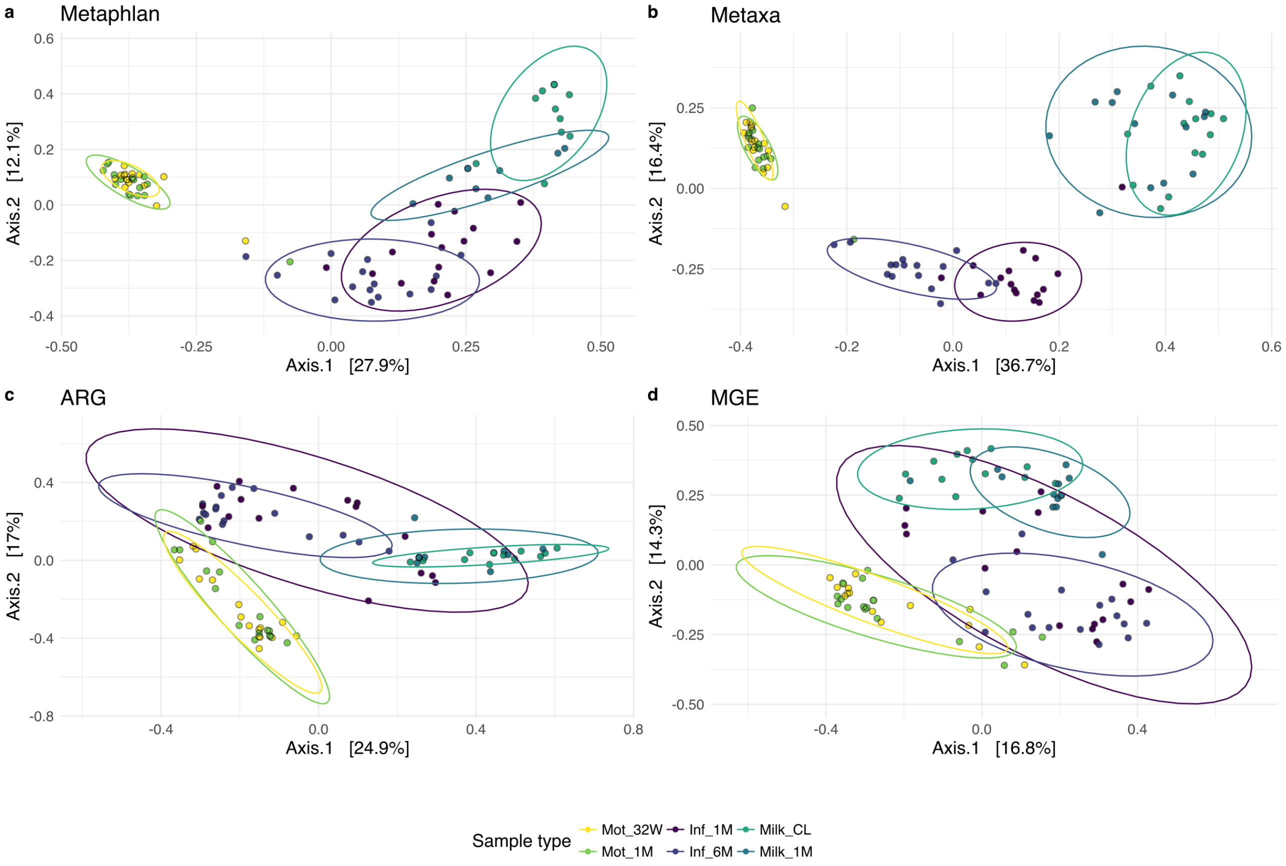

Supplementary Figure 2: PCoA of microbiomes, resistomes and MGEs using presence/absence data.

a, Species level taxonomic identification done based on single copy marker genes with Metaphlan2[1]. **b**, Taxonomic profiling based on 16S rRNA reads retrieved using Metaxa2[2]. **c**, Resistome profiles based on reads mapped against an ARG database and normalized to 16S rRNA gene reads and gene lengths. **d**, MGE profiles based on read mapping against a custom MGE database. Horn-Morisita similarity indexes were used to calculate between-sample overlap for the ordinations. The confidence ellipses are drawn with confidence level of 0.90. The significances and R^2-values of differences between samples are represented in Supplementary Table 2. Sample names are as follows: Inf_1M = one-month-old infants, Inf_6M = six-month-old infants, Mot_32W = mother fecal samples gestational week 32, Mot_1M = mother fecal samples one month postpartum, Milk_CL = colostrum or milk produced within seven days after delivery, Milk_1M = milk one month postpartum.

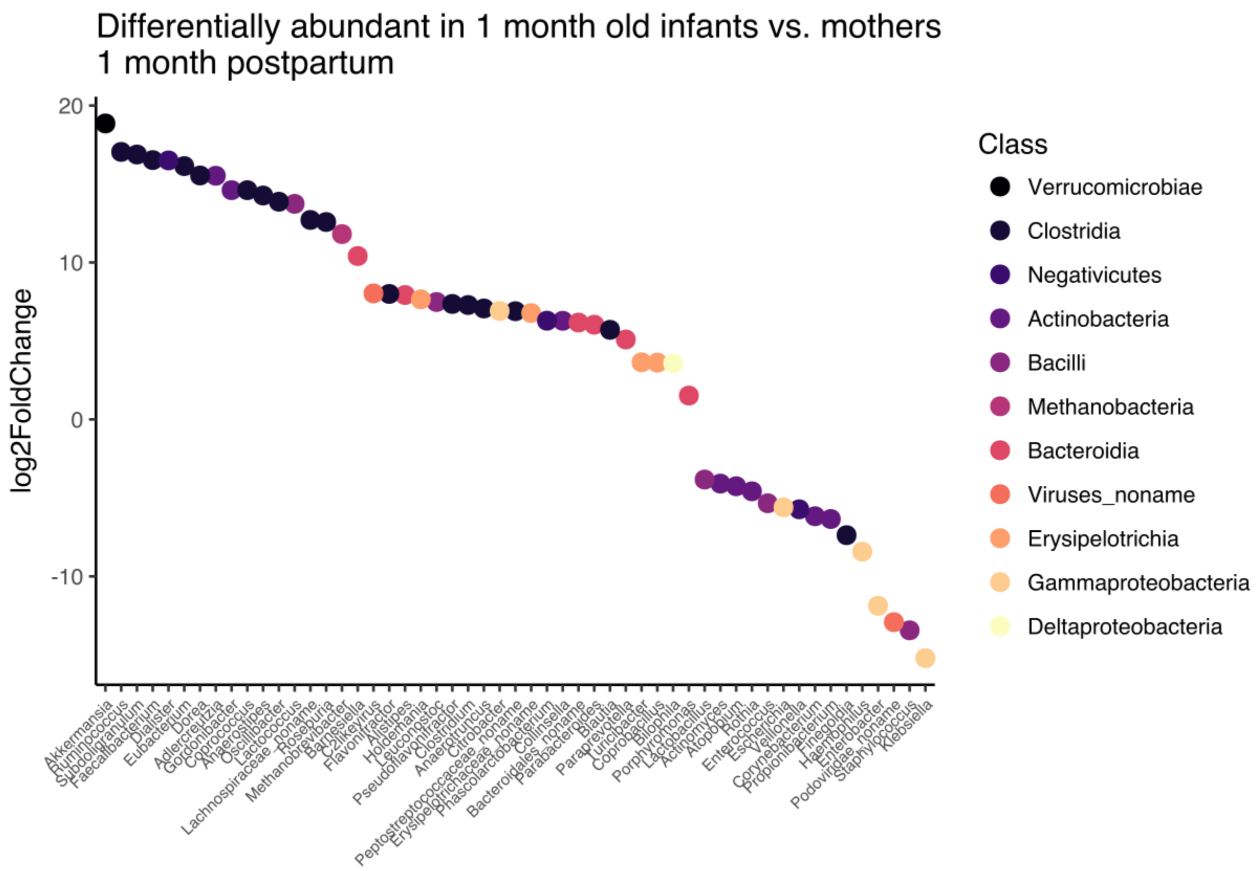

Supplementary Figure 3: Genera differentially abundant in infants and mothers. Analysis is done using DESeq2[3]. The x-axis denotes genus and color class. Negative log fold change values on the y-axis mean that the genus is more abundant in mothers.

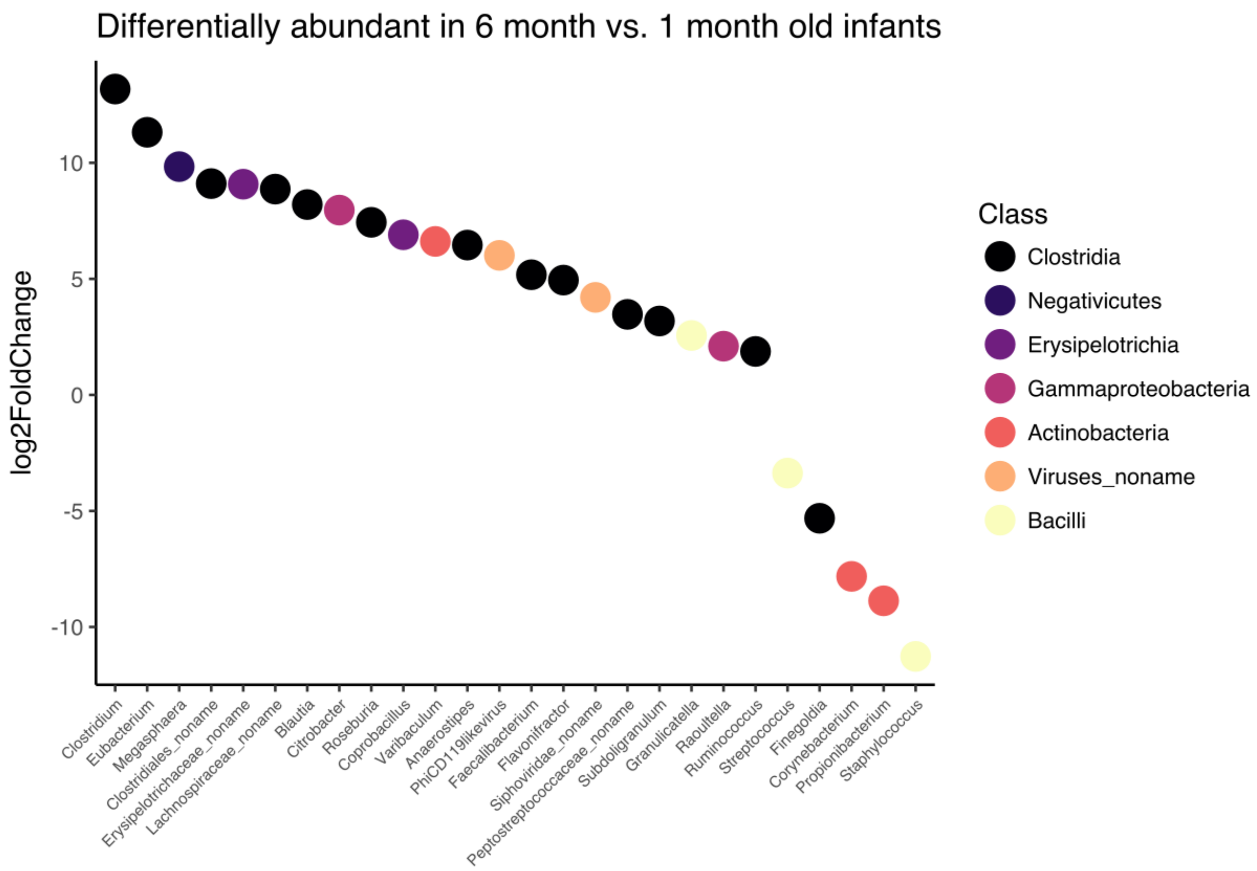

Supplementary Figure 4: Species differentially abundant at one and six months. Analysis done using DESeq2[3]. The x-axis denotes genus and color class. Negative log fold change values on the y-axis mean that the genus is more abundant in mothers.

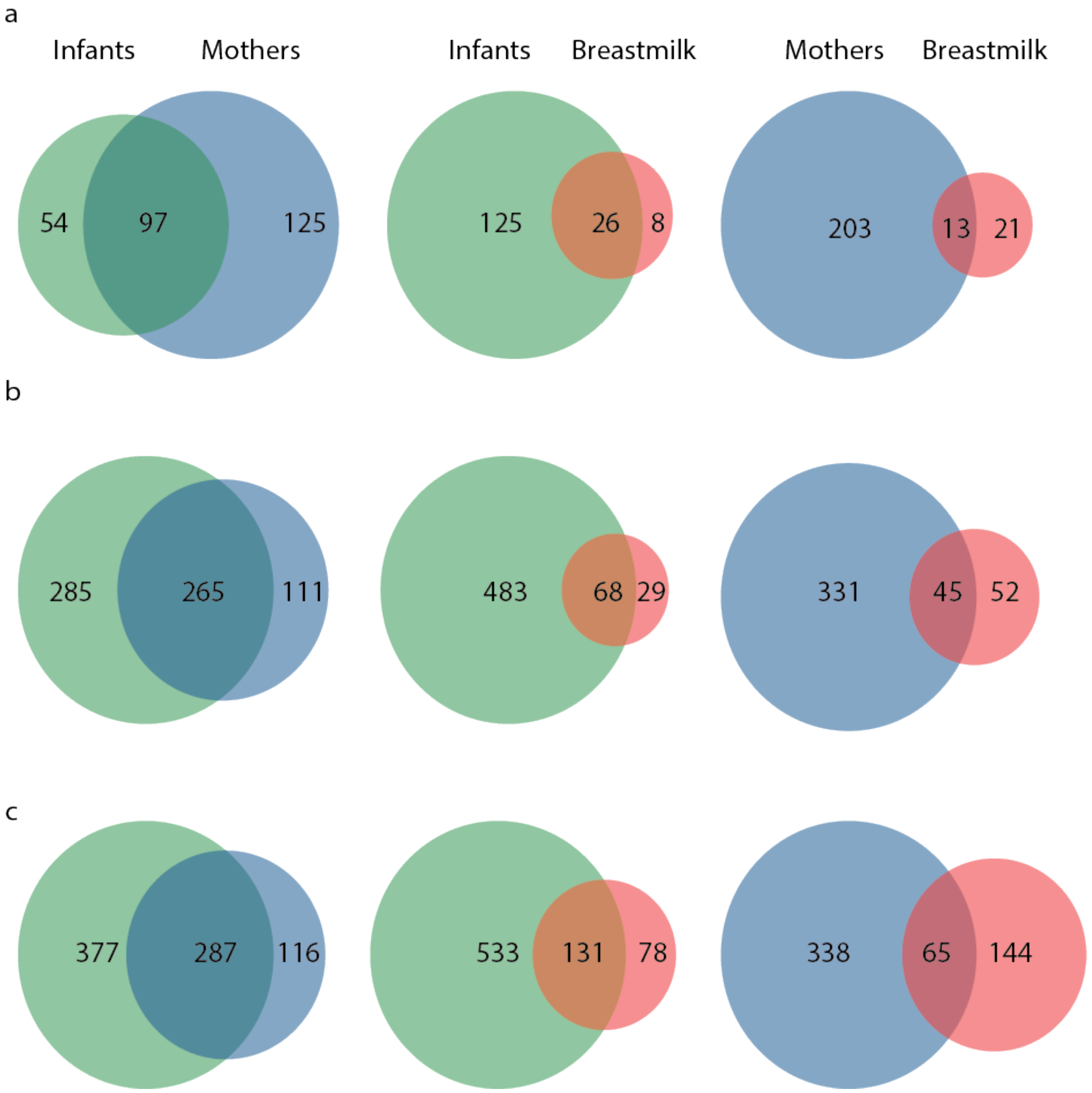

Supplementary Figure 5. Venn diagrams of shared species, ARGs and MGEs between mothers, infants and breast milk

a, Shared species using Metaxa2[2] classification. **b,** Shared ARGs. **c,** Shared MGEs. Colors denote the sample type (infant, mother or breast milk) and both time points for each sample type (32 eeks ante and one month postpartum for mothers and one- and six-month-old infants) have been combined. The size of the larger circle is equal in all comparisons and the size of the smaller circle has been adjusted according to reflect the relative number of species or genes in the sample type with less diversity. The numbers denote the number of species or genes which are shared or only found in one of the sample types. The comparisons were done between all infants and all mothers and sharing was defined as detection of the gene in both sample types compared.

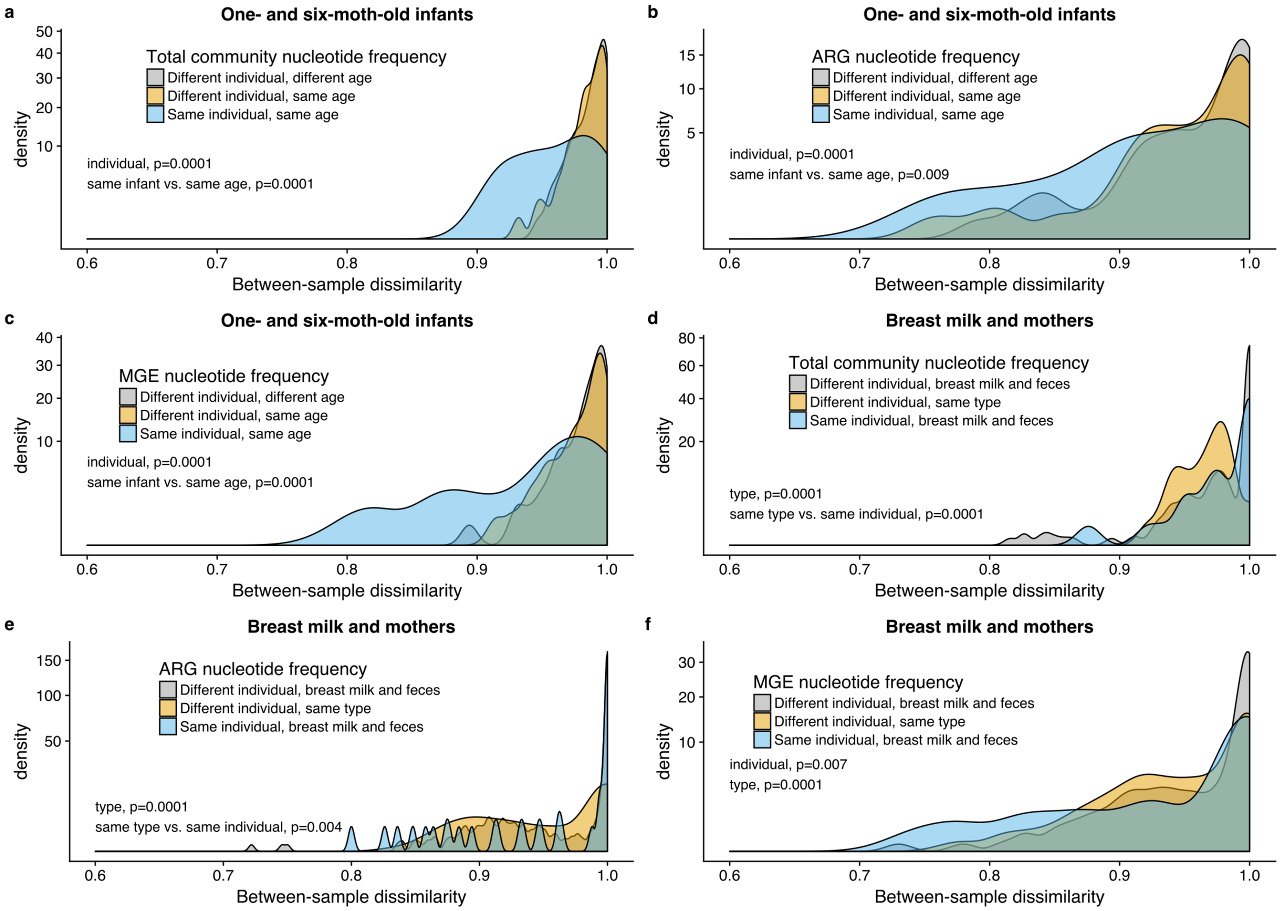

Supplementary Figure 6: Dissimilarities between one and six-month-old infants and mothers' gut and breast milk

a, Dissimilarity of microbial communities between one- and six-month-old-infants using kmer profiles. **b**, Dissimilarity of resistomes between one- and six-month-old-infants using kmer profiles. **c**, Dissimilarity of MGEs between one- and six-month-old-infants using kmer profiles. **d**, Dissimilarity of microbial communities between breast milk and mothers' feces using kmer profiles. **e**, Dissimilarity of resistomes between breast milk and mothers' feces using kmer profiles. **f**, Dissimilarity of MGEs between breast milk and mothers' feces using kmer profiles. Type indicates that mothers and infants or feces and breast milk are significantly different from each other, family indicates that infant's feces are more similar to mother's feces than to unrelated women and individual indicates that infants or mothers are more similar to themselves than to another individual, type vs. individual indicates that feces and breast milk differ significantly in a mother, same infant vs. same age indicates that the infant is more similar to self at a different age than to another infant of the same age. Kmer profiles were calculated using sourmash[4]. Density of the samples in plotted on the y-axis and the x-axis depicts the between-sample dissimilarity calculated with Jaccard similarity index of species, ARGs and MGEs shared between sample types. The x-axis ranges from 0 (no dissimilarity) to 1 (complete dissimilarity). Significance of differences was tested using ANOVA between the similarity indexes in the comparisons and p-values <0.05 are indicated in the figures.

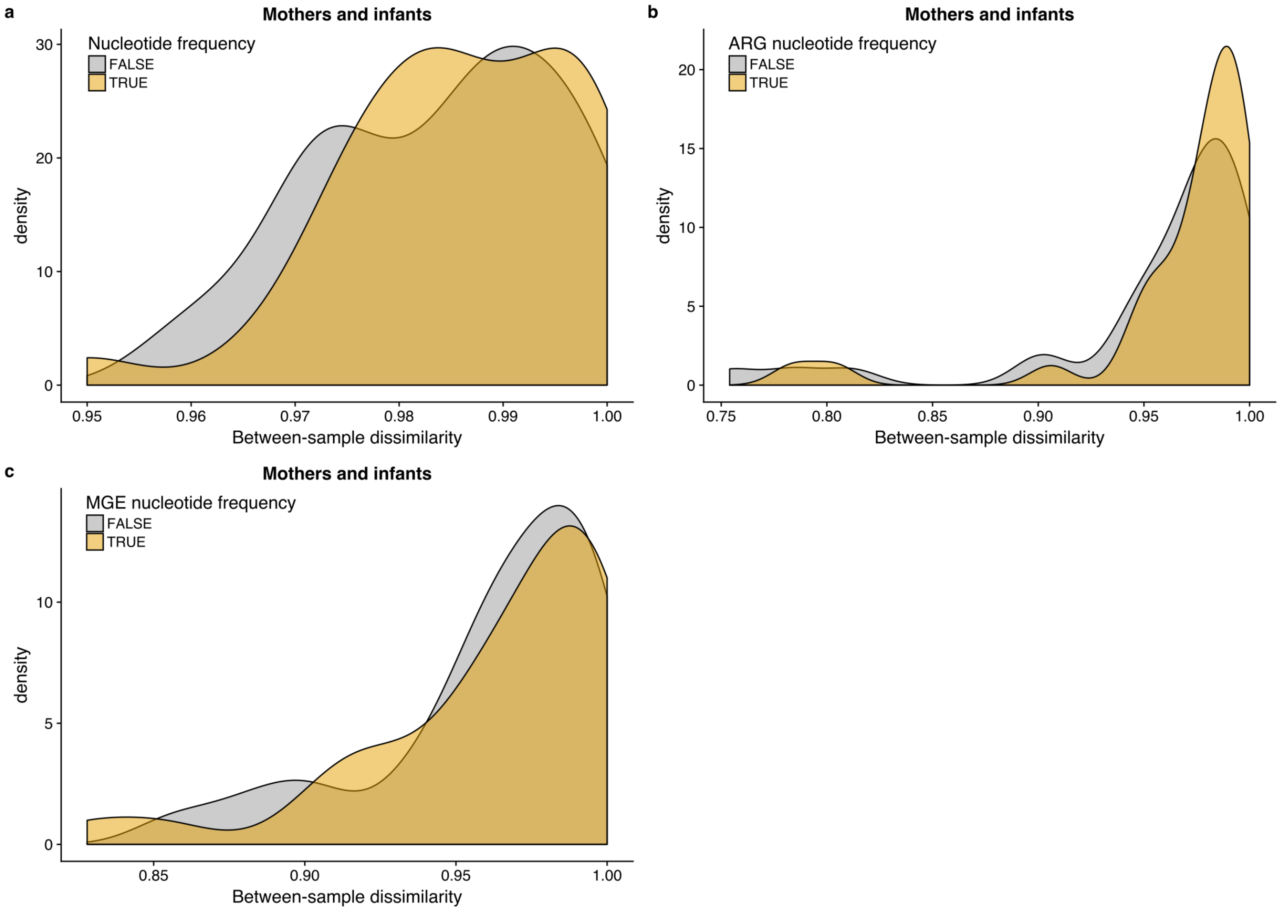

Supplementary Figure 7: Dissimilarities in total microbial community, ARGs and MGEs in relation to sampling time

a, Total community nucleotide frequency profile. No significant differences between similarity of related mother-infant pairs sampled at same or different times (ANOVA, p>0.05). **b**, ARG nucleotide frequency profiles. No significant differences between similarity of related mother-infant pairs sampled at same or different times (ANOVA, p>0.05). **c**, MGE nucleotide frequency profiles. No significant differences between similarity of related mother-infant pairs sampled at same or different times (ANOVA, p>0.05). False indicates that samples were taken at different times and true indicates that samples were taken at the overlapping timepoint of one-month postpartum. Nucleotide frequencies were calculated using sourmash[4].

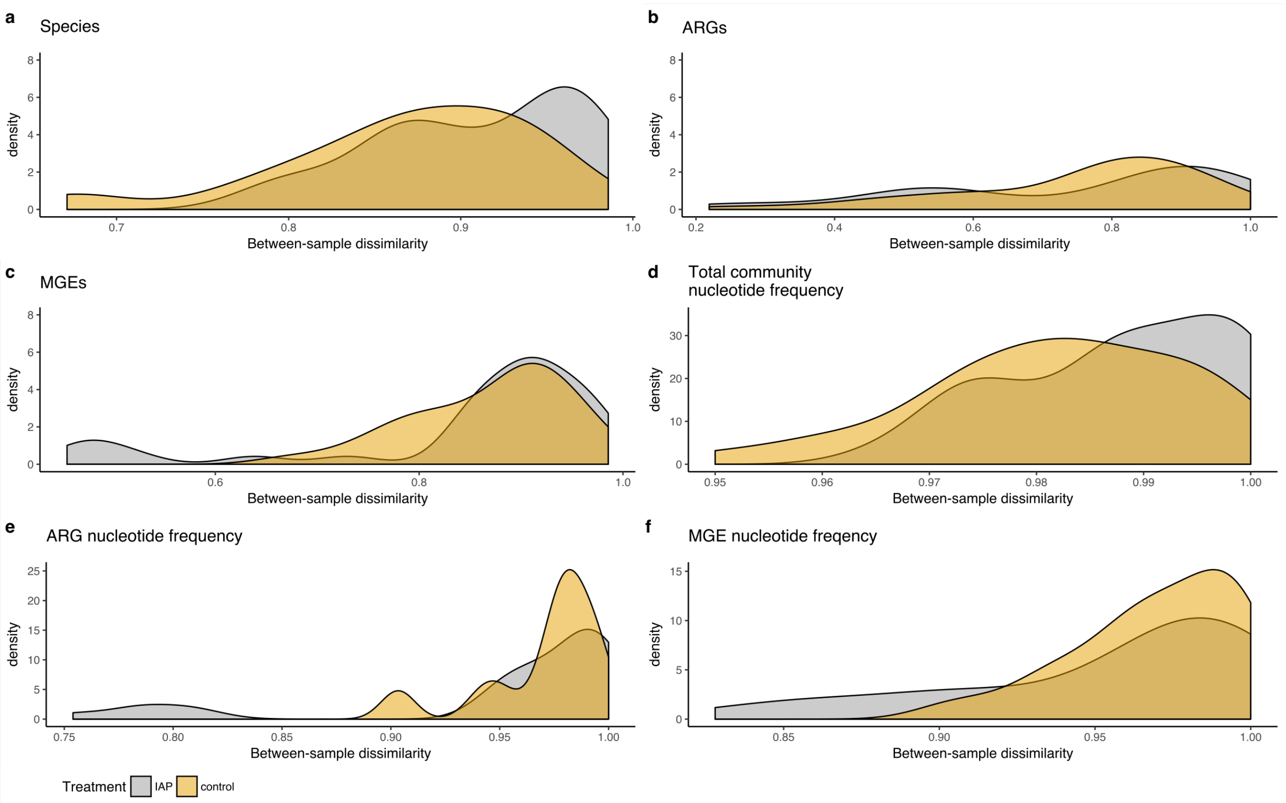

Supplementary Figure 8: Dissimilarities between mothers and their infants in IAP or control groups.

a, Dissimilarity of species in related mother-infant pairs divided by IAP and control groups. Species dissimilarity between infants and their mothers is lower in the control group (ANOVA, p=0.017) **b**, Dissimilarity of ARG types in related mother-infant pairs divided by IAP and control groups. No significant difference between IAP and control groups ANOVA, (p>0.05) **c**, Dissimilarity of MGE types in related mother-infant pairs divided by IAP and control groups. No significant difference between IAP and control groups (ANOVA, p>0.05). **d**, Dissimilarity of total community DNA sequence profiles in related mother-infant pairs divided by IAP and control groups. DNA sequence profile dissimilarity between infants and their mothers is lower in control group (ANOVA, p=0.04) **e,** Dissimilarity of ARG DNA sequence profiles in in related mother-infant pairs divided by IAP and control groups. No significant differences between IAP and control groups (ANOVA, p>0.05). **f**, Dissimilarity of MGE DNA sequence profiles in related mother-infant pairs divided by IAP and control groups. No significant differences between IAP and control groups (ANOVA, p>0.05). Nucleotide frequencies were calculated using sourmash[4].

Supplementary Table 1: Negative binomial GLMs of total relative ARG abundance.

Linear hypotheses	Estimate	Std. Error	z value	Pr(>|z|)	
Inf_6M - Inf_1M == 0	-0.41747	0.38493	-1.085	0.88692	
Milk_1M - Inf_1M == 0	-1.33711	0.45365	-2.947	0.03732	*
Milk_CL - Inf_1M == 0	-1.54570	0.39845	-3.879	0.00144	**
Mot_1M - Inf_1M == 0	-1.99230	0.38495	-5.175	< 0.001	***
Mot_32W - Inf_1M == 0	-1.94961	0.38495	-5.065	< 0.001	***
Milk_1M - Inf_6M == 0	-0.91964	0.45366	-2.027	0.32469	
Milk_CL - Inf_6M == 0	-1.12823	0.39845	-2.832	0.05211	.
Mot_1M - Inf_6M == 0	-1.57483	0.38495	-4.091	< 0.001	***
Mot_32W - Inf_6M == 0	-1.53214	0.38495	-3.980	< 0.001	***
Milk_CL - Milk_1M == 0	-0.20859	0.46519	-0.448	0.99771	
Mot_1M - Milk_1M == 0	-0.65519	0.45368	-1.444	0.69811	
Mot_32W - Milk_1M == 0	-0.61250	0.45367	-1.350	0.75531	
Mot_1M - Milk_CL == 0	-0.44660	0.39847	-1.121	0.87203	
Mot_32W - Milk_CL == 0	-0.40391	0.39847	-1.014	0.91293	
Mot_32W - Mot_1M == 0	0.04269	0.38497	0.07708333	1.00000	

Supplementary Table 2: Negative binomial GLMs of total relative MGE abundance.

Linear Hypotheses:	Estimate	Std. Error	z value	Pr(>|z|)	
Inf_6M - Inf_1M == 0	-0.7559	0.3678	-2.055	0.31081	
Milk_1M - Inf_1M == 0	-0.359	0.3884	-0.924	0.94032	
Milk_CL - Inf_1M == 0	-0.6209	0.3884	-1.599	0.59896	
Mot_1M - Inf_1M == 0	-2.0746	0.3678	-5.641	< 0.001	
Mot_32W - Inf_1M == 0	-2.2291	0.3678	-6.061	< 0.001	***
Milk_1M - Inf_6M == 0	0.3969	0.3884	1.022	0.91061	***
Milk_CL - Inf_6M == 0	0.135	0.3884	0.348	0.99933	
Mot_1M - Inf_6M == 0	-1.3187	0.3678	-3.585	0.00456	
Mot_32W - Inf_6M == 0	-1.4732	0.3678	-4.005	< 0.001	**
Milk_CL - Milk_1M == 0	-0.2619	0.408	-0.642	0.98779	***
Mot_1M - Milk_1M == 0	-1.7156	0.3884	-4.417	< 0.001	
Mot_32W - Milk_1M == 0	-1.8702	0.3884	-4.815	< 0.001	***
Mot_1M - Milk_CL == 0	-1.4537	0.3884	-3.742	0.00253	***
Mot_32W - Milk_CL == 0	-1.6082	0.3884	-4.14	< 0.001	**
Mot_32W - Mot_1M == 0	-0.1545	0.3678	-0.42	0.99833	***

Supplementary Table 3: ANOVA and Tukey's post hoc test of species diversity with Metaphlan2

**Simpson**				
Linear hypotheses	diff	lwr	upr	p adj
Inf_6M-Inf_1M	0.14362589	-0.0847588	0.37201057	0.4482837
Milk_1M-Inf_1M	-0.0432294	-0.3229424	0.23648356	0.9975542
Milk_CL-Inf_1M	-0.0280693	-0.2747529	0.21861439	0.9994413
Mot_1M-Inf_1M	0.33156106	0.10317638	0.55994574	0.0008329
Mot_32W-Inf_1M	0.38022802	0.15184334	0.6086127	0.0000838
Milk_1M-Inf_6M	-0.1868553	-0.4665683	0.09285766	0.3792891
Milk_CL-Inf_6M	-0.1716952	-0.4183788	0.07498849	0.3332022
Mot_1M-Inf_6M	0.18793516	-0.0404495	0.41631984	0.167513
Mot_32W-Inf_6M	0.23660212	0.00821744	0.4649868	0.0378959
Milk_CL-Milk_1M	0.01516015	-0.2796832	0.3100035	0.9999889
Mot_1M-Milk_1M	0.37479046	0.0950775	0.65450343	0.0025642
Mot_32W-Milk_1M	0.42345743	0.14374446	0.70317039	0.0004347
Mot_1M-Milk_CL	0.35963032	0.11294667	0.60631396	0.000782
Mot_32W-Milk_CL	0.40829728	0.16161363	0.65498093	0.0000934
Mot_32W-Mot_1M	0.04866696	-0.1797177	0.27705164	0.9890355
**Shannon**				
Linear hypotheses	diff	lwr	upr	p adj
Inf_6M-Inf_1M	0.39630993	-0.1863505	0.97897033	0.3589164
Milk_1M-Inf_1M	-0.1945036	-0.9081139	0.51910677	0.9673389
Milk_CL-Inf_1M	-0.1597005	-0.7890456	0.46964467	0.9760475
Mot_1M-Inf_1M	1.33645862	0.7537982	1.91911902	0
Mot_32W-Inf_1M	1.58936198	1.0067016	2.17202238	0
Milk_1M-Inf_6M	-0.5908135	-1.3044238	0.12279684	0.1624772
Milk_CL-Inf_6M	-0.5560104	-1.1853556	0.07333474	0.1142213
Mot_1M-Inf_6M	0.94014869	0.3574883	1.52280909	0.0001481
Mot_32W-Inf_6M	1.19305205	0.6103917	1.77571244	0.0000009
Milk_CL-Milk_1M	0.03480307	-0.7174083	0.78701441	0.9999934
Mot_1M-Milk_1M	1.53096218	0.8173519	2.24457252	0.0000003
Mot_32W-Milk_1M	1.78386554	1.0702552	2.49747587	0
Mot_1M-Milk_CL	1.49615911	0.866814	2.12550427	0
Mot_32W-Milk_CL	1.74906247	1.1197173	2.37840763	0
Mot_32W-Mot_1M	0.25290336	-0.329757	0.83556375	0.8012174

Supplementary Table 4: ANOVA and Tukey's post hoc test of species diversity with Metaxa2.

Linear hypotheses	diff	lwr	upr	p adj
**Simpson**				
Inf_6M-Inf_1M	0.02901006	-0.1053052	0.16332533	0.9884821
Milk_1M-Inf_1M	-0.1348162	-0.2766689	0.00703655	0.0721758
Milk_CL-Inf_1M	-0.0622222	-0.2012517	0.0768073	0.7815477
Mot_1M-Inf_1M	0.20550281	0.07118754	0.33981808	0.0003489
Mot_32W-Inf_1M	0.22235117	0.0880359	0.35666644	0.0000868
Milk_1M-Inf_6M	-0.1638263	-0.305679	-0.0219735	0.0140647
Milk_CL-Inf_6M	-0.0912323	-0.2302618	0.04779723	0.4013117
Mot_1M-Inf_6M	0.17649275	0.04217748	0.31080802	0.0032271
Mot_32W-Inf_6M	0.1933411	0.05902584	0.32765637	0.0009122
Milk_CL-Milk_1M	0.07259397	-0.0737304	0.21891831	0.6986277
Mot_1M-Milk_1M	0.340319	0.19846626	0.48217173	0
Mot_32W-Milk_1M	0.35716735	0.21531462	0.49902009	0
Mot_1M-Milk_CL	0.26772503	0.12869552	0.40675454	0.0000036
Mot_32W-Milk_CL	0.28457338	0.14554387	0.4236029	0.0000008
Mot_32W-Mot_1M	0.01684835	-0.1174669	0.15116362	0.9991149
**Shannon**				
Inf_6M-Inf_1M	0.1938482	-0.2804217	0.66811801	0.8396261
Milk_1M-Inf_1M	-0.6031574	-1.1040422	-0.1022726	0.009068
Milk_CL-Inf_1M	-0.3875098	-0.8784258	0.10340608	0.2049132
Mot_1M-Inf_1M	1.3041765	0.8299067	1.77844634	0
Mot_32W-Inf_1M	1.4369362	0.9626663	1.91120601	0
Milk_1M-Inf_6M	-0.7970056	-1.2978904	-0.2961208	0.0001784
Milk_CL-Inf_6M	-0.581358	-1.0722739	-0.0904421	0.0108524
Mot_1M-Inf_6M	1.1103283	0.6360585	1.58459817	0
Mot_32W-Inf_6M	1.243088	0.7688182	1.71735784	0
Milk_CL-Milk_1M	0.2156476	-0.3010265	0.73232166	0.8274311
Mot_1M-Milk_1M	1.9073339	1.4064491	2.40821868	0
Mot_32W-Milk_1M	2.0400936	1.5392088	2.54097835	0
Mot_1M-Milk_CL	1.6916863	1.2007704	2.18260227	0
Mot_32W-Milk_CL	1.824446	1.3335301	2.31536193	0
Mot_32W-Mot_1M	0.1327597	-0.3415102	0.60702951	0.9637959
**Chao1**				
Inf_6M-Inf_1M	3.03825	-3.4026518	9.4791527	0.7414921
Milk_1M-Inf_1M	-4.196373	-10.998724	2.6059775	0.4720917
Milk_CL-Inf_1M	-2.243792	-8.9107596	4.4231748	0.9226797
Mot_1M-Inf_1M	4.852952	-1.5879503	11.2938541	0.2500808
Mot_32W-Inf_1M	7.729609	1.2887072	14.1705117	0.0094111
Milk_1M-Inf_6M	-7.234624	-14.036975	-0.4322729	0.0303614
Milk_CL-Inf_6M	-5.282043	-11.94901	1.3849244	0.2015187
Mot_1M-Inf_6M	1.814701	-4.6262008	8.2556037	0.962782
Mot_32W-Inf_6M	4.691359	-1.7495432	11.1322612	0.2852183
Milk_CL-Milk_1M	1.952581	-5.0641994	8.9693615	0.9646981
Mot_1M-Milk_1M	9.049325	2.2469744	15.8516763	0.0027516
Mot_32W-Milk_1M	11.925983	5.1236319	18.7283338	0.0000281
Mot_1M-Milk_CL	7.096744	0.4297771	13.7637115	0.0301371
Mot_32W-Milk_CL	9.973402	3.3064346	16.6403691	0.0005031
Mot_32W-Mot_1M	2.876658	-3.5642447	9.3175598	0.7830239

Supplementary Table 5: ANOVA and Tukey's post hoc test of ARG diversities.

Linear hypotheses	diff	lwr	upr	p adj
**Simpson**				
Inf_6M-Inf_1M	0.01663435	-0.1966518	0.2299205	0.9999131
Milk_1M-Inf_1M	-0.5759308	-0.827291	-0.3245707	0
Milk_CL-Inf_1M	-0.3926521	-0.6134243	-0.1718799	0.000022
Mot_1M-Inf_1M	0.00097719	-0.212309	0.2142634	1
Mot_32W-Inf_1M	-0.005632	-0.2189182	0.2076542	0.9999996
Milk_1M-Inf_6M	-0.5925652	-0.8439254	-0.341205	0
Milk_CL-Inf_6M	-0.4092865	-0.6300587	-0.1885143	0.0000091
Mot_1M-Inf_6M	-0.0156572	-0.2289434	0.197629	0.9999356
Mot_32W-Inf_6M	-0.0222664	-0.2355526	0.1910198	0.9996353
Milk_CL-Milk_1M	0.18327872	-0.074464	0.4410214	0.3103049
Mot_1M-Milk_1M	0.57690804	0.32554785	0.8282682	0
Mot_32W-Milk_1M	0.57029881	0.31893861	0.821659	0.0000001
Mot_1M-Milk_CL	0.39362932	0.17285713	0.6144015	0.0000209
Mot_32W-Milk_CL	0.38702008	0.1662479	0.6077923	0.0000296
Mot_32W-Mot_1M	-0.0066092	-0.2198954	0.206677	0.9999991
**Shannon**				
Inf_6M-Inf_1M	0.41926341	-0.341346	1.1798728	0.5950694
Milk_1M-Inf_1M	-2.4506716	-3.3470583	-1.5542848	0
Milk_CL-Inf_1M	-2.0953209	-2.8826264	-1.3080153	0
Mot_1M-Inf_1M	-0.0356427	-0.7962521	0.7249667	0.9999931
Mot_32W-Inf_1M	-0.1645773	-0.9251867	0.5960321	0.9883093
Milk_1M-Inf_6M	-2.869935	-3.7663218	-1.9735482	0
Milk_CL-Inf_6M	-2.5145843	-3.3018898	-1.7272787	0
Mot_1M-Inf_6M	-0.4549061	-1.2155155	0.3057033	0.5061592
Mot_32W-Inf_6M	-0.5838407	-1.3444501	0.1767687	0.2310046
Milk_CL-Milk_1M	0.3553507	-0.563797	1.2744985	0.8681005
Mot_1M-Milk_1M	2.41502888	1.5186421	3.3114157	0
Mot_32W-Milk_1M	2.28609424	1.3897075	3.182481	0
Mot_1M-Milk_CL	2.05967818	1.2723727	2.8469837	0
Mot_32W-Milk_CL	1.93074354	1.143438	2.7180491	0
Mot_32W-Mot_1M	-0.1289346	-0.889544	0.6316748	0.9962313
**Chao1**				
Inf_6M-Inf_1M	1.6010274	-3.881412	7.083467	0.9564396
Milk_1M-Inf_1M	-3.3814384	-9.842555	3.079678	0.6476261
Milk_CL-Inf_1M	-2.2813252	-7.956189	3.393538	0.8481134
Mot_1M-Inf_1M	-1.3944486	-6.876888	4.08799	0.9759259
Mot_32W-Inf_1M	-1.8597719	-7.342211	3.622667	0.919803
Milk_1M-Inf_6M	-4.9824658	-11.443582	1.478651	0.2265166
Milk_CL-Inf_6M	-3.8823526	-9.557216	1.792511	0.3530953
Mot_1M-Inf_6M	-2.995476	-8.477915	2.486963	0.6042634
Mot_32W-Inf_6M	-3.4607993	-8.943238	2.02164	0.4447852
Milk_CL-Milk_1M	1.1001132	-5.525063	7.72529	0.9965815
Mot_1M-Milk_1M	1.9869898	-4.474127	8.448106	0.945991
Mot_32W-Milk_1M	1.5216665	-4.93945	7.982783	0.982872
Mot_1M-Milk_CL	0.8868766	-4.787987	6.56174	0.997436
Mot_32W-Milk_CL	0.4215533	-5.25331	6.096417	0.9999317
Mot_32W-Mot_1M	-0.4653233	-5.947762	5.017116	0.999868

Supplementary Table 6: ANOVA and Tukey's post hoc test of MGE diversities.

Linear hypotheses	diff	lwr	upr	p adj
**Simpson**				
Inf_6M-Inf_1M	-0.0475679	-0.310187	0.21505122	0.994882
Milk_1M-Inf_1M	-0.3375626	-0.6149193	-0.0602059	0.0080771
Milk_CL-Inf_1M	-0.0922808	-0.3696375	0.18507594	0.9260267
Mot_1M-Inf_1M	-0.0409091	-0.3035282	0.22171002	0.9974892
Mot_32W-Inf_1M	-0.0821333	-0.3447524	0.18048586	0.9423799
Milk_1M-Inf_6M	-0.2899947	-0.5673514	-0.012638	0.0350834
Milk_CL-Inf_6M	-0.0447129	-0.3220696	0.23264384	0.9970445
Mot_1M-Inf_6M	0.00665879	-0.2559603	0.26927791	0.9999997
Mot_32W-Inf_6M	-0.0345654	-0.2971845	0.22805376	0.9988811
Milk_CL-Milk_1M	0.24528183	-0.0460679	0.53663159	0.1496013
Mot_1M-Milk_1M	0.29665349	0.01929679	0.5740102	0.0289239
Mot_32W-Milk_1M	0.25542934	-0.0219274	0.53278605	0.0888563
Mot_1M-Milk_CL	0.05137166	-0.225985	0.32872836	0.9943192
Mot_32W-Milk_CL	0.01014751	-0.2672092	0.28750421	0.999998
Mot_32W-Mot_1M	-0.0412242	-0.3038433	0.22139497	0.9973958
**Shannon**				
Inf_6M-Inf_1M	0.03839637	-0.9395019	1.01629466	0.9999971
Milk_1M-Inf_1M	-1.5186348	-2.5514105	-0.4858591	0.0006629
Milk_CL-Inf_1M	-0.963001	-1.9957767	0.0697747	0.0819944
Mot_1M-Inf_1M	-0.1957521	-1.1736504	0.78214621	0.9918555
Mot_32W-Inf_1M	-0.4929094	-1.4708077	0.48498889	0.6840274
Milk_1M-Inf_6M	-1.5570312	-2.5898069	-0.5242555	0.0004467
Milk_CL-Inf_6M	-1.0013974	-2.0341731	0.03137832	0.0627474
Mot_1M-Inf_6M	-0.2341485	-1.2120467	0.74374983	0.9816512
Mot_32W-Inf_6M	-0.5313058	-1.509204	0.44659252	0.610876
Milk_CL-Milk_1M	0.55563381	-0.5292469	1.64051455	0.6691684
Mot_1M-Milk_1M	1.32288273	0.29010704	2.35565842	0.004451
Mot_32W-Milk_1M	1.02572542	-0.0070503	2.05850111	0.0526516
Mot_1M-Milk_CL	0.76724892	-0.2655268	1.80002461	0.2643572
Mot_32W-Milk_CL	0.47009161	-0.5626841	1.5028673	0.768984
Mot_32W-Mot_1M	-0.2971573	-1.2750556	0.68074097	0.9488146
**Chao1**				
Inf_6M-Inf_1M	1.1126599	-0.8784189	3.10373862	0.5814277
Milk_1M-Inf_1M	-1.2150055	-3.317819	0.88780805	0.545322
Milk_CL-Inf_1M	-0.9311772	-3.0339907	1.17163632	0.788849
Mot_1M-Inf_1M	0.9990351	-0.9920437	2.9901138	0.6881985
Mot_32W-Inf_1M	1.8527286	-0.1383502	3.8438073	0.0831116
Milk_1M-Inf_6M	-2.3276654	-4.4304789	-0.2248518	0.021239
Milk_CL-Inf_6M	-2.0438371	-4.1466506	0.05897644	0.0616777
Mot_1M-Inf_6M	-0.1136248	-2.1047036	1.87745393	0.9999816
Mot_32W-Inf_6M	0.7400687	-1.2510101	2.73114742	0.8864194
Milk_CL-Milk_1M	0.2838283	-1.9250753	2.49273181	0.9990038
Mot_1M-Milk_1M	2.2140405	0.111227	4.31685406	0.0330908
Mot_32W-Milk_1M	3.067734	0.9649205	5.17054756	0.0007486
Mot_1M-Milk_CL	1.9302123	-0.1726013	4.03302579	0.0907365
Mot_32W-Milk_CL	2.7839058	0.6810922	4.88671929	0.0029393
Mot_32W-Mot_1M	0.8536935	-1.1373853	2.84477225	0.8105183

Supplementary Table 7: ADONIS of ARGs and MGEs

**Comparison**	**R2**	**p**	**adjusted p-value**	**R2**	**p**	**adjusted p-value**
ARGs, relative abundance				ARGs, presence/absence		
Inf_6M - Inf_1M	0.03695	0.287	0.3075	0.06364	0.03	0.03461538
Milk_1M - Inf_1M	0.09274	0.001	0.00125	0.15432	0.001	0.00125
Milk_1M - Inf_6M	0.1266	0.001	0.00125	0.24941	0.001	0.00125
Milk_CL - Inf_1M	0.15383	0.001	0.00125	0.19801	0.001	0.00125
Milk_CL - Inf_6M	0.18841	0.001	0.00125	0.30891	0.001	0.00125
Milk_CL - Milk_1M	0.074	0.023	0.02653846	0.06036	0.154	0.165
Mot_1M - Inf_1M	0.21718	0.001	0.00125	0.21383	0.001	0.00125
Mot_1M - Inf_6M	0.22258	0.001	0.00125	0.24086	0.001	0.00125
Mot_1M - Milk_1M	0.22842	0.001	0.00125	0.29903	0.001	0.00125
Mot_1M - Milk_CL	0.30412	0.001	0.00125	0.35166	0.001	0.00125
Mot_32W - Inf_1M	0.26222	0.001	0.00125	0.2426	0.001	0.00125
Mot_32W - Inf_6M	0.28479	0.001	0.00125	0.284	0.001	0.00125
Mot_32W - Milk_1M	0.2685	0.001	0.00125	0.35982	0.001	0.00125
Mot_32W - Milk_CL	0.34418	0.001	0.00125	0.30748	0.001	0.00125
Mot_32W - Mot_1M	0.02066	0.779	0.779	0.00937	0.996	0.996
MGEs, relative abundance				MGEs, presence/absence		
Inf_6M - Inf_1M	0.04042	0.148	0.15857143	0.05212	0.049	0.0525
Milk_1M - Inf_1M	0.0633	0.001	0.00125	0.09805	0.001	0.00125
Milk_1M - Inf_6M	0.10324	0.001	0.00125	0.16795	0.001	0.00125
Milk_CL - Inf_1M	0.10061	0.001	0.00125	0.15229	0.001	0.00125
Milk_CL - Inf_6M	0.13862	0.001	0.00125	0.24948	0.001	0.00125
Milk_CL - Milk_1M	0.0668	0.002	0.00230769	0.11661	0.001	0.00125
Mot_1M - Inf_1M	0.12787	0.001	0.00125	0.17159	0.001	0.00125
Mot_1M - Inf_6M	0.10721	0.001	0.00125	0.22153	0.001	0.00125
Mot_1M - Milk_1M	0.15874	0.001	0.00125	0.23902	0.001	0.00125
Mot_1M - Milk_CL	0.19678	0.001	0.00125	0.28965	0.001	0.00125
Mot_32W - Inf_1M	0.15094	0.001	0.00125	0.17415	0.001	0.00125
Mot_32W - Inf_6M	0.17504	0.001	0.00125	0.23598	0.001	0.00125
Mot_32W - Milk_1M	0.17504	0.001	0.00125	0.24232	0.001	0.00125
Mot_32W - Milk_CL	0.21548	0.001	0.00125	0.28528	0.001	0.00125
Mot_32W - Mot_1M	0.0314	0.524	0.524	0.02748	0.623	0.623

Supplementary Table 8: ADONIS of microbial communities

**Comparison**	**R2**	**p**	**adjusted p-value**	**R2**	**p**	**adjusted p-value**
Metaphlan2, relative abundance				Metaphlan2, presence/absence		
Inf_6M - Inf_1M	0.0877	0.001	0.00115385	0.0877	0.001	0.00125
Milk_1M - Inf_1M	0.10161	0.001	0.00115385	0.10161	0.004	0.00462
Milk_1M - Inf_6M	0.19753	0.001	0.00115385	0.19753	0.001	0.00125
Milk_CL - Inf_1M	0.21746	0.001	0.00115385	0.21746	0.001	0.00125
Milk_CL - Inf_6M	0.31893	0.001	0.00115385	0.31893	0.001	0.00125
Milk_CL - Milk_1M	0.11617	0.023	0.02464286	0.11617	0.027	0.0289
Mot_1M - Inf_1M	0.3332	0.001	0.00115385	0.3332	0.001	0.00125
Mot_1M - Inf_6M	0.32817	0.001	0.00115385	0.32817	0.001	0.00125
Mot_1M - Milk_1M	0.36052	0.001	0.00115385	0.36052	0.001	0.00125
Mot_1M - Milk_CL	0.48467	0.001	0.00115385	0.48467	0.001	0.00125
Mot_32W - Inf_1M	0.33996	0.001	0.00115385	0.33996	0.001	0.00125
Mot_32W - Inf_6M	0.33691	0.001	0.00115385	0.33691	0.001	0.00125
Mot_32W - Milk_1M	0.36564	0.001	0.00115385	0.36564	0.001	0.00125
Mot_32W - Milk_CL	0.48856	0.001	0.00115385	0.48856	0.001	0.00125
Mot_32W - Mot_1M	0.01178	0.983	0.983	0.01178	0.987	0.987
Metaxa2, relative abundance				Metaxa2, presence/absence		
Inf_6M - Inf_1M	0.04096	0.237	0.25392857	0.14092	0.001	0.00125
Milk_1M - Inf_1M	0.22975	0.001	0.00125	0.24148	0.001	0.00125
Milk_1M - Inf_6M	0.26217	0.001	0.00125	0.32694	0.001	0.00125
Milk_CL - Inf_1M	0.29679	0.001	0.00125	0.32491	0.001	0.00125
Milk_CL - Inf_6M	0.33276	0.001	0.00125	0.43541	0.001	0.00125
Milk_CL - Milk_1M	0.07908	0.011	0.01269231	0.06321	0.069	0.0739
Mot_1M - Inf_1M	0.30421	0.001	0.00125	0.51296	0.001	0.00125
Mot_1M - Inf_6M	0.25396	0.001	0.00125	0.42003	0.001	0.00125
Mot_1M - Milk_1M	0.37087	0.001	0.00125	0.44339	0.001	0.00125
Mot_1M - Milk_CL	0.45026	0.001	0.00125	0.57213	0.001	0.00125
Mot_32W - Inf_1M	0.32584	0.001	0.00125	0.54677	0.001	0.00125
Mot_32W - Inf_6M	0.28342	0.001	0.00125	0.46887	0.001	0.00125
Mot_32W - Milk_1M	0.37084	0.001	0.00125	0.46028	0.001	0.00125
Mot_32W - Milk_CL	0.44995	0.001	0.00125	0.59201	0.001	0.00125
Mot_32W - Mot_1M	0.02548	0.644	0.644	0.02457	0.732	0.732

Supplementary References

1. Segata, N. *et al*. Metagenomic microbial community profiling using unique clade-specific marker genes. *Nat. Methods* **9,** 811–814 (2012).
2. Bengtsson-Palme, J. *et al*. METAXA2: Improved identification and taxonomic classification of small and large subunit rRNA in metagenomic data. *Mol. Ecol. Resour.* **15,** 1403–1414 (2015).
3. Love, M. I., Huber, W. & Anders, S. Moderated estimation of fold change and dispersion for RNA-seq data with DESeq2. *Genome Biol.* **15,** (2014).
4. Titus Brown, C. & Irber, L. sourmash: a library for MinHash sketching of DNA. *J. Open Source Softw.* **1,** 27 (2016).
