# Supplementary material for: Maternal gut and breast milk microbiota affect infant gut antibiotic resistome and mobile genetic elements
Source: Nat Commun. 2018 Sep 24;9:3891. doi: 10.1038/s41467-018-06393-w (PMC6155145; doi:10.1038/s41467-018-06393-w)
Supplement: Supplementary file 3 — Description of Additional Supplementary Files [file 41467_2018_6393_MOESM3_ESM.pdf]

Description of Additional Supplementary Files

File Name: Supplementary Software
Description: Supplementary Software: Custom R-scripts related to statistical analysis of the data

File Name: Supplementary Dataset 1
Description: Supplementary Data 1: Sample metadata

File Name: Supplementary Dataset 2
Description: Supplementary Data 2: Fecal sample sequencing data

File Name: Supplementary Dataset 3
Description: Supplementary Data 3: Breast milk sample sequencing data

File Name: Supplementary Dataset 4
Description: Supplementary Data 4: Microbial taxa correlations with ARGs and MGEs

File Name: Supplementary Dataset 5
Description: Supplementary Data 5: ARG containing MGE contig sharing
