# Supplementary material for: Maternal gut and breast milk microbiota affect infant gut antibiotic resistome and mobile genetic elements
Source: Nat Commun. 2018 Sep 24;9:3891. doi: 10.1038/s41467-018-06393-w (PMC6155145; doi:10.1038/s41467-018-06393-w)
Supplement: Supplementary file 9 — Supplementary Software [file 41467_2018_6393_MOESM9_ESM.zip › Supplementary_methods2.html]

Milk\_metagenomes


# Milk\_metagenomes

## Set environment

Acquire required packages and set working directory.

```
library(formatR)
library(reshape2)
library(vegan)
library(dplyr)
library(phyloseq)
library(reshape2)
library(ggplot2)
library(knitr)
library(corrplot)
library(psych)
library(DESeq2)
library(rmarkdown)
library(cowplot)
library(car)
library(multcomp)
library(glmnet)
library(VennDiagram)
library(Hmisc)
library(plyr)
library(viridis)
setwd("/Users/kparnane/Documents/Maito/Nextseq_milk1-2/")
```

## Read in metaphlan results on species level

The abundance\_species.txt file is created from the merged abundance table from Metaphlan. Lines with ’’ are picked and the lines with ’t\_\_’ are removed. The abundance table then has only species level entries. Column with OTU and running number is added after this.

```
metaphlan_sp <- as.matrix(read.table("abundances_species.txt", fill = 1, header = T, 
    row.names = 1, check.names = F))
```

## Read in taxonomy table for species level

Taxonomy table is created from the species level merged abundance table with awk and sed scripts.

```
tax_sp <- read.table(("tax_table_species.txt"), fill = 1, row.names = 1)
tax_sp <- apply(tax_sp, 2, function(y) (gsub(".__", "", y)))
```

## Read in metadata

```
sample_data <- read.table(as.matrix("Milk_metadataNEW2.txt"), fill = 1, header = T, 
    row.names = 1, stringsAsFactors = FALSE)

# Change the Type codes
sample_data$TYPE[sample_data$TYPE == "RM1"] <- "Milk_CL"
sample_data$TYPE[sample_data$TYPE == "RM2"] <- "Milk_1M"
sample_data$TYPE[sample_data$TYPE == "LU2"] <- "Inf_1M"
sample_data$TYPE[sample_data$TYPE == "LU4"] <- "Inf_6M"
sample_data$TYPE[sample_data$TYPE == "AU1"] <- "Mot_32W"
sample_data$TYPE[sample_data$TYPE == "AU2"] <- "Mot_1M"
```

## Merge into a phyloseq object

```
PHY_SP <- phyloseq(otu_table(metaphlan_sp, taxa_are_rows = TRUE), tax_table(as.matrix(tax_sp)), 
    sample_data(sample_data))
```

## Change the taxonomic levels to something meaningful

```
colnames(tax_table(PHY_SP)) <- c("Domain", "Phylum", "Class", "Order", "Family", 
    "Genus", "Species")
# Remove viruses
PHY_SP <- subset_taxa(PHY_SP, Domain != "Viruses")
```

## Remove samples without any classified taxa

There are some milk samples without any classified species with Metaphlan.

```
PHY_SP <- subset_samples(PHY_SP, sample_sums(PHY_SP) != 0)
```

The sample names are: Mot\_32W=Mother’s fecal samples (32 weeks gestational time), Mot\_1M=Mother’s fecal samples (1 month post partum), Inf\_1M=Infant fecal samples (1 month), Inf\_6M=Infant fecal samples (6 months), Milk\_CL=Breast milk (colostrum), Milk\_1M=Breast milk (1 month) AB=intrapartum IV antibiotic treatment CON=no antibiotic treatment

# Analysis with Metaxa 16S rRNA data

```
# Read in metaxa OTU table and tax table
metaxa <- as.matrix(read.table("metaxa_otu_table.txt", fill = 1, header = T, 
    row.names = 1, check.names = F))
metaxa_tax <- read.table(("metaxa_tax.txt"), fill = 1, row.names = 1, header = F)

# Make phyloseq object
PHY_mtx <- phyloseq(otu_table(metaxa, taxa_are_rows = TRUE), tax_table(as.matrix(metaxa_tax)), 
    sample_data(sample_data))


# Change the levels to something meaningful
colnames(tax_table(PHY_mtx)) <- c("Domain", "Phylum", "Class", "Order", "Family", 
    "Genus")

# Exclude Eukaryota and unknown domains and samples with less than 20 16S
# SSU matches
PHY_mtx_mod <- subset_taxa(PHY_mtx, !Domain %in% c("Eukaryota", "unknown"))
PHY_mtx <- subset_samples(PHY_mtx_mod, sample_sums(PHY_mtx) > 20)

# Turn into relative data
PHY_mtx_rel <- transform_sample_counts(PHY_mtx, function(x) x/sum(x))
```

# Analysis of ARGs and MGEs

## Read in ARG and MGE bowtie2 mapping results

```
ARG_bt <- as.matrix(read.table("ARG_genemat.txt", fill = 1, header = T, row.names = 1, 
    check.names = F))
MGE_bt <- as.matrix((read.table("genemat_MGE.txt", fill = 1, header = T, row.names = 1, 
    check.names = F)))
ARG_tax <- read.table("ARG_tax_table_final.txt", fill = 1, row.names = 1, header = F)
MGE_tax <- read.table("MGE_tax_table.txt", fill = 1, row.names = 1, header = F)

# Modify the taxonomy table
MGE_tax$V2 <- gsub("_", " ", MGE_tax$V2)
MGE_tax$V2 <- gsub("insertion element ", "", MGE_tax$V2)
ARG_tax$V2 <- gsub("_resistance", "", ARG_tax$V2)
ARG_tax$V2 <- gsub("_", " ", ARG_tax$V2)
```

## Normalize with SSU counts from Metaxa

First read in metaxa otu table and then remove Eukaryotic and unknown domains. Take sample sums from the modified metaxa phyloseq object and use for normalization.

Use gene lengths to normalize the mapping results. Remove genes without any matches and samples without any matches (some breast milk samples have no reads mapping to ARGs or MGEs).

```
# Normalize ARG and MGE counts
SSU_counts <- as.matrix(sample_sums(PHY_mtx_mod))
ARG_lengths <- as.matrix(read.table("ARG_genelenghts.txt", fill = 1, header = F, 
    row.names = 1, check.names = T))

# Divide by ARG gene lengths
arg_length_norm <- ARG_bt/ARG_lengths[, 1]

# Divide by SSU counts and normalize to 16S rRNA length (1541)
ARG_SSU_length_norm <- t(t(arg_length_norm)/SSU_counts[, 1]) * 1541

# Replace NAs with 0
ARG_SSU_length_norm[is.na(ARG_SSU_length_norm)] <- 0

# Repeat for MGEs
MGE_length <- as.matrix(read.table("MGE_genelenghts.txt", fill = 1, header = F, 
    row.names = 1, check.names = F))
mge_length_norm <- MGE_bt/MGE_length[, 1]
MGE_SSU_length_norm <- t(t(mge_length_norm)/SSU_counts[, 1]) * 1541
MGE_SSU_length_norm[is.na(MGE_SSU_length_norm)] <- 0

# Make phyloseq object without normalizing the 16S counts
ARG_noSSU <- phyloseq(otu_table(arg_length_norm, taxa_are_rows = T), sample_data(sample_data))
sample_data(ARG_noSSU)$SSU <- SSU_counts[, 1]

# Make phyloseq objects from ARG and MGE matrixes
ARG <- phyloseq(otu_table(ARG_SSU_length_norm, taxa_are_rows = T), sample_data(sample_data), 
    tax_table(as.matrix(ARG_tax)))
MGE <- phyloseq(otu_table(MGE_SSU_length_norm, taxa_are_rows = T), sample_data(sample_data), 
    tax_table(as.matrix(MGE_tax)))

# Remove samples with no matches
ARG_mod <- subset_samples(ARG, sample_sums(ARG) != 0)

MGE_mod <- subset_samples(MGE, sample_sums(MGE) != 0)

# Remove the genes which have no matches
ARG_mod2 <- otu_table(ARG_mod)[rowSums(otu_table(ARG_mod)) > 0]
ARG_PHY <- phyloseq(ARG_mod2, sample_data(sample_data), tax_table(as.matrix(ARG_tax)))
ARG_PHY <- subset_taxa(ARG_PHY, !V2 %in% c("Bifidobacteria"))
MGE_mod2 <- otu_table(MGE_mod)[rowSums(otu_table(MGE_mod)) > 0]
MGE_PHY <- phyloseq(MGE_mod2, sample_data(sample_data), tax_table(as.matrix(MGE_tax)))
```

# Make ordinations to be used in publication

Ordinations are done using Horn-Morisita similarity index and PCoA.

```
# Save plots

PHY_SP_ord <- ordinate(PHY_SP, method = "PCoA", distance = "horn")

p <- plot_ordination(PHY_SP, PHY_SP_ord, color = "TYPE")
metaphlan.plot <- p + scale_color_viridis(discrete = TRUE) + geom_point(colour = "black", 
    pch = 21, size = 2, alpha = 0.5) + stat_ellipse(level = 0.9, linetype = 1) + 
    theme_minimal() + labs(title = "Metaphlan")

PHY_mtx_ord <- ordinate(PHY_mtx_rel, method = "PCoA", distance = "horn")
p <- plot_ordination(PHY_mtx_rel, PHY_mtx_ord, color = "TYPE")
metaxa.plot <- p + scale_color_viridis(discrete = TRUE) + geom_point(colour = "black", 
    pch = 21, size = 2, alpha = 0.5) + stat_ellipse(level = 0.9, linetype = 1) + 
    theme_minimal() + labs(title = "Metaxa")

ARG_PHY_ord <- ordinate(ARG_PHY, "PCoA", "horn")
ARG.plot <- p <- plot_ordination(ARG_PHY, ARG_PHY_ord, color = "TYPE", title = "PCoA ARGs") + 
    scale_color_viridis(discrete = TRUE) + geom_point(colour = "black", pch = 21, 
    size = 2, alpha = 0.5) + theme_minimal() + stat_ellipse(linetype = 1) + 
    labs(title = "ARG")

MGE_PHY_ord <- ordinate(MGE_PHY, "PCoA", "horn")
p <- plot_ordination(MGE_PHY, MGE_PHY_ord, color = "TYPE", title = "PCoA MGEs")
MGE.plot <- p + scale_color_brewer(palette = "Paired", "Sample\ntype") + geom_point(colour = "black", 
    pch = 21, size = 2, alpha = 0.5) + theme_minimal() + stat_ellipse(linetype = 1) + 
    labs(title = "MGE")

plot_grid(metaphlan.plot, metaxa.plot, ARG.plot, MGE.plot, labels = c("auto"), 
    align = "h")
```

# Check if the differences is distances are significant using adonis

```
# Check if the separations are significant, do this for all pairwise
# comparisons

ARG_temp <- subset_samples(ARG_PHY, (TYPE == "Inf_6M" | TYPE == "Inf_1M"))
ARG_dist <- vegdist(t(otu_table(ARG_temp)), dist = "horn")
adonis(ARG_dist ~ TYPE, data = data.frame(sample_data(ARG_temp), permutations = 9999))
```

```
## 
## Call:
## adonis(formula = ARG_dist ~ TYPE, data = data.frame(sample_data(ARG_temp),      permutations = 9999)) 
## 
## Permutation: free
## Number of permutations: 999
## 
## Terms added sequentially (first to last)
## 
##           Df SumsOfSqs MeanSqs F.Model      R2 Pr(>F)
## TYPE       1    0.4406 0.44057   1.151 0.03695  0.272
## Residuals 30   11.4832 0.38277         0.96305       
## Total     31   11.9238                 1.00000
```

```
# After this adjust p-values with p-adjust, method='fdr'
```

# Make diversity plots and ARG + MGE sum plots using negative binomial distibution models to be used in publication

Diversity is calculated using Shannon and sums are relative abundance sums of all the genes based on 16S rRNA gene counts.

```
# Save plots
df <- data.frame(DIV = diversity(t(otu_table(PHY_SP))), TYPE = sample_data(PHY_SP)$TYPE)
metaphlan.plot <- ggplot(df, aes(x = TYPE, y = DIV, fill = TYPE, alpha = 0.5)) + 
    geom_boxplot() + scale_fill_brewer(palette = "Paired", "Sample\ntype") + 
    theme_minimal() + guides(fill = FALSE, alpha = FALSE) + theme(axis.text.x = element_text(angle = 90)) + 
    labs(y = "Shannon diversity", x = "") + ggtitle("Metaphlan")

df <- data.frame(DIV = diversity(t(otu_table(PHY_mtx_rel))), TYPE = sample_data(PHY_mtx_rel)$TYPE)
metaxa.plot <- ggplot(df, aes(x = TYPE, y = DIV, fill = TYPE, alpha = 0.5)) + 
    geom_boxplot() + scale_fill_brewer(palette = "Paired", "Sample\ntype") + 
    theme_minimal() + guides(fill = FALSE, alpha = FALSE) + theme(axis.text.x = element_text(angle = 90)) + 
    labs(y = "Shannon diversity", x = "") + ggtitle("Metaxa")


df <- data.frame(DIV = diversity(t(otu_table(ARG_PHY))), TYPE = sample_data(ARG_PHY)$TYPE)
ARG.plot <- ggplot(df, aes(x = TYPE, y = DIV, fill = TYPE, alpha = 0.5)) + geom_boxplot() + 
    scale_fill_brewer(palette = "Paired", "Sample\ntype") + theme_minimal() + 
    guides(fill = FALSE, alpha = FALSE) + theme(axis.text.x = element_text(angle = 90)) + 
    labs(title = "ARGs", y = "Shannon diversity", x = "")

df <- data.frame(DIV = diversity(t(otu_table(MGE_PHY))), TYPE = sample_data(MGE_PHY)$TYPE)
MGE.plot <- ggplot(df, aes(x = TYPE, y = DIV, fill = TYPE, alpha = 0.5)) + geom_boxplot() + 
    scale_fill_brewer(palette = "Paired", "Sample\ntype") + theme_minimal() + 
    guides(fill = FALSE, alpha = FALSE) + theme(axis.text.x = element_text(angle = 90)) + 
    labs(title = "MGEs", y = "Shannon diverisity", x = "")

# Save mean 16S read count in fecal samples
mean16S_counts_fec <- mean(SSU_counts[grep("RM", row.names(SSU_counts), invert = TRUE), 
    ])

# Get integers for negative binomial distribution by multiplying the
# relative gene counts to 16S with mean 16S counts
dfA <- data.frame(SUM = round(mean16S_counts_fec * (sample_sums(otu_table(ARG_PHY)))), 
    TYPE = sample_data(ARG_PHY)$TYPE)

# Get the predictors for mean and SE from negative binomial model and save
# to dataframe
fit <- glm.nb(SUM ~ TYPE, data = dfA, link = log)

dfA <- cbind(dfA, Mean = predict(fit, newdata = dfA, type = "response"), SE = predict(fit, 
    newdata = dfA, type = "response", se.fit = T)$se.fit)

ARG.sum.plot <- ggplot(dfA, aes(x = TYPE, y = Mean)) + scale_color_brewer(palette = "Paired") + 
    geom_line() + geom_jitter(data = dfA, aes(x = TYPE, y = SUM, color = TYPE), 
    size = 1.3, alpha = 0.5, width = 0.3) + geom_errorbar(aes(ymin = Mean - 
    SE, ymax = Mean + SE), width = 0.3, lwd = 0.3) + geom_point(size = 0.9) + 
    theme_minimal() + theme(axis.text.x = element_text(angle = 90)) + labs(y = "Sum abundance/16S", 
    x = "") + guides(color = FALSE, alpha = FALSE) + labs(title = "ARGs") + 
    scale_y_log10()


# MGEs Get integers for negative binomial distribution by multiplying the
# relative gene counts to 16S with mean 16S counts
dfM <- data.frame(SUM = round(mean16S_counts_fec * (sample_sums(otu_table(MGE_PHY)))), 
    TYPE = sample_data(MGE_PHY)$TYPE)

# Get the predictors for mean and SE from negative binomial model and save
# to dataframe
fit <- glm.nb(SUM ~ TYPE, data = dfM, link = log)

dfM <- cbind(dfM, Mean = predict(fit, newdata = dfM, type = "response"), SE = predict(fit, 
    newdata = dfM, type = "response", se.fit = T)$se.fit)

MGE.sum.plot <- ggplot(dfM, aes(x = TYPE, y = Mean)) + scale_color_brewer(palette = "Paired") + 
    geom_line() + geom_jitter(data = dfM, aes(x = TYPE, y = SUM, color = TYPE), 
    size = 1.3, alpha = 0.5, width = 0.3) + geom_errorbar(aes(ymin = Mean - 
    SE, ymax = Mean + SE), width = 0.3, lwd = 0.3) + geom_point(size = 0.9) + 
    theme_minimal() + theme(axis.text.x = element_text(angle = 90)) + labs(y = "Sum abundance/16S", 
    x = "") + guides(color = FALSE, alpha = FALSE) + labs(title = "MGEs") + 
    scale_y_log10()

plot_grid(metaphlan.plot, metaxa.plot, ARG.plot, MGE.plot, ARG.sum.plot, MGE.sum.plot, 
    labels = c("auto"), align = "v", ncol = 2, label_size = 10)
```

# Check are the differences in sums significant using negative binomial GLMS

Use mean 16S counts for normalization.

```
# Test statistical significances between total sums abundances of the
# different types

# Save mean 16S read count in fecal samples
mean16S_counts_fec <- mean(SSU_counts[grep("RM", row.names(SSU_counts), invert = TRUE), 
    ])

# ARGs Get integers for negative binomial distribution by multiplying the
# relative gene counts to 16S with mean 16S counts
df <- data.frame(SUM = round(mean16S_counts_fec * (sample_sums(otu_table(ARG_PHY)))), 
    TYPE = sample_data(ARG_PHY)$TYPE)

fit <- glm.nb(SUM ~ TYPE, data = df, link = log)

# Tukey's post hoc test
glht.mod <- glht(fit, mcp(TYPE = "Tukey"))
summary(glht(glht.mod))
```

```
## 
##   Simultaneous Tests for General Linear Hypotheses
## 
## Linear Hypotheses:
##                        Estimate Std. Error z value Pr(>|z|)    
## Inf_6M - Inf_1M == 0   -0.41747    0.38493  -1.085  0.88689    
## Milk_1M - Inf_1M == 0  -1.33711    0.45365  -2.947  0.03754 *  
## Milk_CL - Inf_1M == 0  -1.54570    0.39845  -3.879  0.00145 ** 
## Mot_1M - Inf_1M == 0   -1.99230    0.38495  -5.175  < 0.001 ***
## Mot_32W - Inf_1M == 0  -1.94961    0.38495  -5.065  < 0.001 ***
## Milk_1M - Inf_6M == 0  -0.91964    0.45366  -2.027  0.32477    
## Milk_CL - Inf_6M == 0  -1.12823    0.39845  -2.832  0.05208 .  
## Mot_1M - Inf_6M == 0   -1.57483    0.38495  -4.091  < 0.001 ***
## Mot_32W - Inf_6M == 0  -1.53214    0.38495  -3.980  < 0.001 ***
## Milk_CL - Milk_1M == 0 -0.20859    0.46519  -0.448  0.99771    
## Mot_1M - Milk_1M == 0  -0.65519    0.45368  -1.444  0.69820    
## Mot_32W - Milk_1M == 0 -0.61250    0.45367  -1.350  0.75537    
## Mot_1M - Milk_CL == 0  -0.44660    0.39847  -1.121  0.87206    
## Mot_32W - Milk_CL == 0 -0.40391    0.39847  -1.014  0.91293    
## Mot_32W - Mot_1M == 0   0.04269    0.38497   0.111  1.00000    
## ---
## Signif. codes:  0 '***' 0.001 '**' 0.01 '*' 0.05 '.' 0.1 ' ' 1
## (Adjusted p values reported -- single-step method)
```

```
# MGEs
df <- data.frame(SUM = round(mean16S_counts_fec * (sample_sums(otu_table(MGE_PHY)))), 
    TYPE = sample_data(MGE_PHY)$TYPE)

fit <- glm.nb(SUM ~ TYPE, data = df, link = log)

# Tukey's post hoc test
glht.mod <- glht(fit, mcp(TYPE = "Tukey"))
summary(glht(glht.mod))
```

```
## 
##   Simultaneous Tests for General Linear Hypotheses
## 
## Linear Hypotheses:
##                        Estimate Std. Error z value Pr(>|z|)    
## Inf_6M - Inf_1M == 0    -0.7499     0.3690  -2.032  0.32358    
## Milk_1M - Inf_1M == 0   -0.3461     0.3897  -0.888  0.94940    
## Milk_CL - Inf_1M == 0   -0.6322     0.3897  -1.622  0.58333    
## Mot_1M - Inf_1M == 0    -2.0834     0.3690  -5.646  < 0.001 ***
## Mot_32W - Inf_1M == 0   -2.2385     0.3690  -6.066  < 0.001 ***
## Milk_1M - Inf_6M == 0    0.4038     0.3897   1.036  0.90572    
## Milk_CL - Inf_6M == 0    0.1177     0.3897   0.302  0.99967    
## Mot_1M - Inf_6M == 0    -1.3335     0.3690  -3.614  0.00414 ** 
## Mot_32W - Inf_6M == 0   -1.4886     0.3690  -4.034  < 0.001 ***
## Milk_CL - Milk_1M == 0  -0.2861     0.4094  -0.699  0.98209    
## Mot_1M - Milk_1M == 0   -1.7373     0.3897  -4.458  < 0.001 ***
## Mot_32W - Milk_1M == 0  -1.8924     0.3897  -4.856  < 0.001 ***
## Mot_1M - Milk_CL == 0   -1.4512     0.3897  -3.723  0.00275 ** 
## Mot_32W - Milk_CL == 0  -1.6063     0.3897  -4.121  < 0.001 ***
## Mot_32W - Mot_1M == 0   -0.1551     0.3691  -0.420  0.99833    
## ---
## Signif. codes:  0 '***' 0.001 '**' 0.01 '*' 0.05 '.' 0.1 ' ' 1
## (Adjusted p values reported -- single-step method)
```

```
# Next we want to see if there are differences in the species, ARGs or MGEs
# due to antibiotic use or breastfeeding.

# Check for all treatments and all sample types
PHY_SPInf_1M <- subset_samples(PHY_SP, (TYPE == "Inf_1M"))
phy_distSP <- vegdist(t(otu_table(PHY_SPInf_1M)), dist = "horn")
adonis(phy_distSP ~ GROUP, data = data.frame(sample_data(PHY_SPInf_1M)), permutations = 99999)
```

```
## 
## Call:
## adonis(formula = phy_distSP ~ GROUP, data = data.frame(sample_data(PHY_SPInf_1M)),      permutations = 99999) 
## 
## Permutation: free
## Number of permutations: 99999
## 
## Terms added sequentially (first to last)
## 
##           Df SumsOfSqs MeanSqs F.Model      R2  Pr(>F)  
## GROUP      1    0.8094 0.80936  2.1683 0.13411 0.04775 *
## Residuals 14    5.2258 0.37327         0.86589          
## Total     15    6.0352                 1.00000          
## ---
## Signif. codes:  0 '***' 0.001 '**' 0.01 '*' 0.05 '.' 0.1 ' ' 1
```

# Check are the differences in diversity between types significant with ANOVA and Tukey’s test.

```
# Shannon diversity species
a0 <- aov(diversity(t(otu_table(PHY_SP))) ~ sample_data(PHY_SP)$TYPE, data = as.data.frame(otu_table(PHY_SP)))
TukeyHSD(a0)
```

```
##   Tukey multiple comparisons of means
##     95% family-wise confidence level
## 
## Fit: aov(formula = diversity(t(otu_table(PHY_SP))) ~ sample_data(PHY_SP)$TYPE, data = as.data.frame(otu_table(PHY_SP)))
## 
## $`sample_data(PHY_SP)$TYPE`
##                        diff        lwr        upr     p adj
## Inf_6M-Inf_1M    0.39630993 -0.1863505 0.97897033 0.3589164
## Milk_1M-Inf_1M  -0.19450356 -0.9081139 0.51910677 0.9673389
## Milk_CL-Inf_1M  -0.15970049 -0.7890456 0.46964467 0.9760475
## Mot_1M-Inf_1M    1.33645862  0.7537982 1.91911902 0.0000000
## Mot_32W-Inf_1M   1.58936198  1.0067016 2.17202238 0.0000000
## Milk_1M-Inf_6M  -0.59081350 -1.3044238 0.12279684 0.1624772
## Milk_CL-Inf_6M  -0.55601042 -1.1853556 0.07333474 0.1142213
## Mot_1M-Inf_6M    0.94014869  0.3574883 1.52280909 0.0001481
## Mot_32W-Inf_6M   1.19305205  0.6103917 1.77571244 0.0000009
## Milk_CL-Milk_1M  0.03480307 -0.7174083 0.78701441 0.9999934
## Mot_1M-Milk_1M   1.53096218  0.8173519 2.24457252 0.0000003
## Mot_32W-Milk_1M  1.78386554  1.0702552 2.49747587 0.0000000
## Mot_1M-Milk_CL   1.49615911  0.8668140 2.12550427 0.0000000
## Mot_32W-Milk_CL  1.74906247  1.1197173 2.37840763 0.0000000
## Mot_32W-Mot_1M   0.25290336 -0.3297570 0.83556375 0.8012174
```

```
# Shannon diversity metaxa
a1 <- aov(diversity(t(otu_table(PHY_mtx))) ~ sample_data(PHY_mtx)$TYPE, data = as.data.frame(otu_table(PHY_mtx)))
TukeyHSD(a1)
```

```
##   Tukey multiple comparisons of means
##     95% family-wise confidence level
## 
## Fit: aov(formula = diversity(t(otu_table(PHY_mtx))) ~ sample_data(PHY_mtx)$TYPE, data = as.data.frame(otu_table(PHY_mtx)))
## 
## $`sample_data(PHY_mtx)$TYPE`
##                       diff        lwr         upr     p adj
## Inf_6M-Inf_1M    0.1938482 -0.2804217  0.66811801 0.8396261
## Milk_1M-Inf_1M  -0.6031574 -1.1040422 -0.10227263 0.0090680
## Milk_CL-Inf_1M  -0.3875098 -0.8784258  0.10340608 0.2049132
## Mot_1M-Inf_1M    1.3041765  0.8299067  1.77844634 0.0000000
## Mot_32W-Inf_1M   1.4369362  0.9626663  1.91120601 0.0000000
## Milk_1M-Inf_6M  -0.7970056 -1.2978904 -0.29612080 0.0001784
## Milk_CL-Inf_6M  -0.5813580 -1.0722739 -0.09044209 0.0108524
## Mot_1M-Inf_6M    1.1103283  0.6360585  1.58459817 0.0000000
## Mot_32W-Inf_6M   1.2430880  0.7688182  1.71735784 0.0000000
## Milk_CL-Milk_1M  0.2156476 -0.3010265  0.73232166 0.8274311
## Mot_1M-Milk_1M   1.9073339  1.4064491  2.40821868 0.0000000
## Mot_32W-Milk_1M  2.0400936  1.5392088  2.54097835 0.0000000
## Mot_1M-Milk_CL   1.6916863  1.2007704  2.18260227 0.0000000
## Mot_32W-Milk_CL  1.8244460  1.3335301  2.31536193 0.0000000
## Mot_32W-Mot_1M   0.1327597 -0.3415102  0.60702951 0.9637959
```

```
# Shannon diversity ARGs
a2 <- aov(diversity(t(otu_table(ARG_PHY))) ~ sample_data(ARG_PHY)$TYPE, data = as.data.frame(otu_table(ARG_PHY)))
TukeyHSD(a2)
```

```
##   Tukey multiple comparisons of means
##     95% family-wise confidence level
## 
## Fit: aov(formula = diversity(t(otu_table(ARG_PHY))) ~ sample_data(ARG_PHY)$TYPE, data = as.data.frame(otu_table(ARG_PHY)))
## 
## $`sample_data(ARG_PHY)$TYPE`
##                        diff        lwr        upr     p adj
## Inf_6M-Inf_1M    0.41926341 -0.3413460  1.1798728 0.5950694
## Milk_1M-Inf_1M  -2.45067157 -3.3470583 -1.5542848 0.0000000
## Milk_CL-Inf_1M  -2.09532087 -2.8826264 -1.3080153 0.0000000
## Mot_1M-Inf_1M   -0.03564269 -0.7962521  0.7249667 0.9999931
## Mot_32W-Inf_1M  -0.16457733 -0.9251867  0.5960321 0.9883093
## Milk_1M-Inf_6M  -2.86993498 -3.7663218 -1.9735482 0.0000000
## Milk_CL-Inf_6M  -2.51458428 -3.3018898 -1.7272787 0.0000000
## Mot_1M-Inf_6M   -0.45490610 -1.2155155  0.3057033 0.5061592
## Mot_32W-Inf_6M  -0.58384074 -1.3444501  0.1767687 0.2310046
## Milk_CL-Milk_1M  0.35535070 -0.5637970  1.2744985 0.8681005
## Mot_1M-Milk_1M   2.41502888  1.5186421  3.3114157 0.0000000
## Mot_32W-Milk_1M  2.28609424  1.3897075  3.1824810 0.0000000
## Mot_1M-Milk_CL   2.05967818  1.2723727  2.8469837 0.0000000
## Mot_32W-Milk_CL  1.93074354  1.1434380  2.7180491 0.0000000
## Mot_32W-Mot_1M  -0.12893464 -0.8895440  0.6316748 0.9962313
```

```
# Shannon diversity MGEs
a3 <- aov(diversity(t(otu_table(MGE_PHY))) ~ sample_data(MGE_PHY)$TYPE, data = as.data.frame(otu_table(MGE_PHY)))
TukeyHSD(a3)
```

```
##   Tukey multiple comparisons of means
##     95% family-wise confidence level
## 
## Fit: aov(formula = diversity(t(otu_table(MGE_PHY))) ~ sample_data(MGE_PHY)$TYPE, data = as.data.frame(otu_table(MGE_PHY)))
## 
## $`sample_data(MGE_PHY)$TYPE`
##                        diff         lwr         upr     p adj
## Inf_6M-Inf_1M    0.04686588 -0.93547738  1.02920915 0.9999925
## Milk_1M-Inf_1M  -1.51968878 -2.55715889 -0.48221866 0.0007034
## Milk_CL-Inf_1M  -0.97840779 -2.01587791  0.05906232 0.0760458
## Mot_1M-Inf_1M   -0.20352030 -1.18586356  0.77882296 0.9904608
## Mot_32W-Inf_1M  -0.50396532 -1.48630858  0.47837795 0.6675790
## Milk_1M-Inf_6M  -1.56655466 -2.60402478 -0.52908455 0.0004356
## Milk_CL-Inf_6M  -1.02527368 -2.06274379  0.01219644 0.0546407
## Mot_1M-Inf_6M   -0.25038618 -1.23272945  0.73195708 0.9758175
## Mot_32W-Inf_6M  -0.55083120 -1.53317446  0.43151206 0.5777946
## Milk_CL-Milk_1M  0.54128099 -0.54853101  1.63109299 0.6973852
## Mot_1M-Milk_1M   1.31616848  0.27869836  2.35363859 0.0050007
## Mot_32W-Milk_1M  1.01572346 -0.02174665  2.05319358 0.0585276
## Mot_1M-Milk_CL   0.77488749 -0.26258263  1.81235761 0.2588298
## Mot_32W-Milk_CL  0.47444247 -0.56302764  1.51191259 0.7654763
## Mot_32W-Mot_1M  -0.30044502 -1.28278828  0.68189825 0.9474280
```

# Barplots of most abundant ARGs and MGEs and taxa by sample type

Descriptive figures of genes and taxa found in the samples

```
# Barplot most abundant ARGs Glom to class level

ARG_PHY_V2 <- tax_glom(ARG_PHY, taxrank = "V2")

# Take 12 most abundant
ARG_PHY_V2_abund <- prune_taxa(names(sort(taxa_sums(ARG_PHY_V2), TRUE)[1:12]), 
    ARG_PHY_V2)

# Remove samples with none of the 12 most abundant ARG classes (one milk
# sample)
ARG_PHY_V2_abund <- subset_samples(ARG_PHY_V2_abund, sample_sums(ARG_PHY_V2_abund) != 
    0)

# Normalize by number of samples in each sample type
otu_table(ARG_PHY_V2_abund) <- (otu_table(ARG_PHY_V2_abund)[, ]/as.matrix(table(sample_data(ARG_PHY_V2_abund)$TYPE))[, 
    1])

# Plot
p <- plot_bar(ARG_PHY_V2_abund, "TYPE", fill = "V2")
a <- p + geom_bar(aes(color = V2, fill = V2), stat = "identity", position = "stack") + 
    scale_color_brewer(palette = "Set3", "Resistance class") + scale_fill_brewer(palette = "Set3", 
    "Resistance class") + ylab("Sum abundance/16S") + xlab("")

# Repeat for MGEs Barplot 12 most abundant MGEs
MGE_PHY_V2 <- tax_glom(MGE_PHY, taxrank = "V2")
MGE_PHY_V2_abund <- prune_taxa(names(sort(taxa_sums(MGE_PHY_V2), TRUE)[1:12]), 
    MGE_PHY_V2)

# Normalize by number of samples in each sample type
otu_table(MGE_PHY_V2_abund) <- (otu_table(MGE_PHY_V2_abund)[, ]/as.matrix(table(sample_data(MGE_PHY_V2_abund)$TYPE))[, 
    1])

p <- plot_bar(MGE_PHY_V2_abund, "TYPE", fill = "V2")
b <- p + geom_bar(aes(color = V2, fill = V2), stat = "identity", position = "stack") + 
    scale_color_brewer(palette = "Set3", "MGE class") + scale_fill_brewer(palette = "Set3", 
    "MGE class") + ylab("Sum abundance/16S") + xlab("")


# Repeat for Genus Barplot Genus
PHY_SP_genus <- tax_glom(PHY_SP, taxrank = "Genus")
PHY_SP_genus_abun <- prune_taxa(names(sort(taxa_sums(PHY_SP_genus), TRUE)[1:12]), 
    PHY_SP_genus)
mrg <- merge_samples(PHY_SP_genus_abun, "TYPE")

# Normalize by number of samples in each sample type
otu_table(mrg) <- otu_table(mrg)[, ]/as.matrix(table(sample_data(PHY_SP)$TYPE))[, 
    1]
p <- plot_bar(mrg)
c <- p + geom_bar(aes(fill = Genus), stat = "identity", position = "stack") + 
    scale_fill_brewer(palette = "Set3", "Genus") + ylab("Relative abundance (%)") + 
    xlab("")

# Repeat for class Barplot Class
PHY_SP_class <- tax_glom(PHY_SP, taxrank = "Class")
PHY_SP_class_abun <- prune_taxa(names(sort(taxa_sums(PHY_SP_class), TRUE)[1:12]), 
    PHY_SP_class)

# Normalize by number of samples in each sample type
mrg <- merge_samples(PHY_SP_class_abun, "TYPE")
otu_table(mrg) <- otu_table(mrg)[, ]/as.matrix(table(sample_data(PHY_SP)$TYPE))[, 
    1]
p <- plot_bar(mrg)
d <- p + geom_bar(aes(fill = Class), stat = "identity", position = "stack") + 
    scale_fill_brewer(palette = "Set3", "Class") + ylab("Relative abundance (%)") + 
    xlab("")

# Make cowplot
plot_grid(a, b, c, d, labels = "auto", align = "h")
```

# Check the effect of treatments on sample types and differences between classes of ARGs and MGEs using negative binomial GLMs. Check if there are differences between different types of samples in the ARG and MGE sums.

```
# Treatments, check sample types and treatments with the glm.nb
df <- data.frame(SUM = round(mean16S_counts_fec * sample_sums(otu_table(subset_samples(ARG_PHY, 
    TYPE == "Milk_CL")))), TYPE = sample_data(subset_samples(ARG_PHY, TYPE == 
    "Milk_CL"))$TYPE, GROUP = sample_data(subset_samples(ARG_PHY, TYPE == "Milk_CL"))$GROUP, 
    BFAT6MO = sample_data(subset_samples(ARG_PHY, TYPE == "Milk_CL"))$BFAT6MO, 
    PROB = sample_data(subset_samples(ARG_PHY, TYPE == "Milk_CL"))$PROB)

# Change to wanted parameter and test
fit <- glm.nb(SUM ~ GROUP, data = df, link = log)

# Test are the genes classes significantly different with negative binomial
# GLMs, repeat for all classes and MGEs
df <- data.frame(SUM = round(mean16S_counts_fec * (sample_sums(subset_taxa(subset_samples(ARG_PHY, 
    TYPE %in% c("Mot_32W", "Mot_1M", "Inf_1M", "Inf_6M")), V2 == "tetracycline")))), 
    TYPE = sample_data(subset_samples(ARG_PHY, TYPE %in% c("Mot_32W", "Mot_1M", 
        "Inf_1M", "Inf_6M")))$TYPE)

fit <- glm.nb(SUM ~ TYPE, data = df, link = log)

glht.mod <- glht(fit, mcp(TYPE = "Tukey"))
summary(glht(glht.mod))
```

```
## Warning in chkdots(...): Argument(s) 'complete' passed to '...' are ignored

## Warning in chkdots(...): Argument(s) 'complete' passed to '...' are ignored
```

```
## 
##   Simultaneous Tests for General Linear Hypotheses
## 
## Linear Hypotheses:
##                       Estimate Std. Error z value Pr(>|z|)   
## Inf_6M - Inf_1M == 0   -0.2925     0.4127  -0.709  0.89368   
## Mot_1M - Inf_1M == 0    0.9807     0.4126   2.377  0.08165 . 
## Mot_32W - Inf_1M == 0   1.2265     0.4126   2.973  0.01545 * 
## Mot_1M - Inf_6M == 0    1.2732     0.4127   3.085  0.01113 * 
## Mot_32W - Inf_6M == 0   1.5190     0.4127   3.681  0.00139 **
## Mot_32W - Mot_1M == 0   0.2458     0.4125   0.596  0.93335   
## ---
## Signif. codes:  0 '***' 0.001 '**' 0.01 '*' 0.05 '.' 0.1 ' ' 1
## (Adjusted p values reported -- single-step method)
```

# Mantel test for comparing species and ARG and MGE distance matrixes

```
# Test if community is explaining the ARGs, make distance matrices
PHY_bacteria <- subset_taxa(PHY_SP, Domain %in% c("Bacteria"))
PHY_bacteria <- subset_samples(PHY_bacteria, sample_sums(PHY_bacteria) != 0)

# Calculate Horn-Morisita distances
ARG_DIST <- vegdist(t(as.matrix(otu_table(ARG_PHY))), method = "horn")
MGE_DIST <- vegdist(t(as.matrix(otu_table(MGE_PHY))), method = "horn")

# Make intersects of samples found in both distance matrixes
tempAS <- otu_table(PHY_bacteria)[, sample_names(PHY_bacteria) %in% c(intersect(sample_names(PHY_bacteria), 
    sample_names(ARG_PHY)))]

tempSA <- otu_table(ARG_PHY)[, sample_names(ARG_PHY) %in% c(intersect(sample_names(ARG_PHY), 
    sample_names(PHY_bacteria)))]


ARG_BACT_dist <- vegdist(t(as.matrix(tempSA)), method = "horn")


tempMS <- otu_table(PHY_bacteria)[, sample_names(PHY_bacteria) %in% c(intersect(sample_names(PHY_bacteria), 
    sample_names(MGE_PHY)))]

tempSM <- otu_table(MGE_PHY)[, sample_names(MGE_PHY) %in% c(intersect(sample_names(MGE_PHY), 
    sample_names(PHY_bacteria)))]

MGE_bact_dist <- vegdist(t(as.matrix(tempSM)), method = "horn")

PHY_bact_DIST_ARG <- vegdist(t(as.matrix(tempAS)), method = "horn")
PHY_bact_DIST_MGE <- vegdist(t(as.matrix(tempMS)), method = "horn")

# Do a mantel test for ARGs+MGEs and taxa distance matrixes
mantel(ARG_BACT_dist, PHY_bact_DIST_ARG, method = "kendall")
```

```
## 
## Mantel statistic based on Kendall's rank correlation tau 
## 
## Call:
## mantel(xdis = ARG_BACT_dist, ydis = PHY_bact_DIST_ARG, method = "kendall") 
## 
## Mantel statistic r: 0.4661 
##       Significance: 0.001 
## 
## Upper quantiles of permutations (null model):
##    90%    95%  97.5%    99% 
## 0.0429 0.0546 0.0668 0.0903 
## Permutation: free
## Number of permutations: 999
```

```
mantel(MGE_bact_dist, PHY_bact_DIST_MGE, method = "kendall")
```

```
## 
## Mantel statistic based on Kendall's rank correlation tau 
## 
## Call:
## mantel(xdis = MGE_bact_dist, ydis = PHY_bact_DIST_MGE, method = "kendall") 
## 
## Mantel statistic r: 0.4993 
##       Significance: 0.001 
## 
## Upper quantiles of permutations (null model):
##    90%    95%  97.5%    99% 
## 0.0494 0.0642 0.0788 0.0938 
## Permutation: free
## Number of permutations: 999
```

# Do a Mantel test also for relation between ARGs and MGEs

```
# Do a mantel test for ARG and MGE distance matrixes
tempAM <- otu_table(MGE_PHY)[, sample_names(MGE_PHY) %in% c(intersect(sample_names(MGE_PHY), 
    sample_names(ARG_PHY)))]
tempMA <- otu_table(ARG_PHY)[, sample_names(ARG_PHY) %in% c(intersect(sample_names(ARG_PHY), 
    sample_names(MGE_PHY)))]

MGE_AM_dist <- vegdist(t(as.matrix(tempAM)), method = "horn")
ARG_MA_dist <- vegdist(t(as.matrix(tempMA)), method = "horn")
mantel(MGE_AM_dist, ARG_MA_dist, method = "kendall")
```

```
## 
## Mantel statistic based on Kendall's rank correlation tau 
## 
## Call:
## mantel(xdis = MGE_AM_dist, ydis = ARG_MA_dist, method = "kendall") 
## 
## Mantel statistic r: 0.4506 
##       Significance: 0.001 
## 
## Upper quantiles of permutations (null model):
##    90%    95%  97.5%    99% 
## 0.0497 0.0631 0.0783 0.0950 
## Permutation: free
## Number of permutations: 999
```

# Check which taxa correlate with ARGs and MGEs using GLM nb

Get candidiate taxa for major ARG and MGE carriers.

```
############ Check correlating taxa#############

########### Inf_6M############## Subset
ARG_PHY_LU <- subset_samples(ARG_PHY, TYPE %in% c("Inf_6M"))
MGE_PHY_LU <- subset_samples(MGE_PHY, TYPE %in% c("Inf_6M"))
PHY_SP_LU <- subset_samples(PHY_SP, TYPE %in% c("Inf_6M"))

############### MGEs############
genus = c()
class = c()
pnlb = c()
pnlb2 = c()
estimate = c()


# Glom to Genus level
PHY_SP_LU_gen <- tax_glom(PHY_SP_LU, taxrank = "Genus")

# Prune out taxa with mean abundance of less than 1% in the sample set
PHY_SP_LU_gen <- subset_taxa(PHY_SP_LU_gen, taxa_sums(PHY_SP_LU_gen) > 16)

# Count correlations for each Genus and the MGE sums
for (i in seq(1, nrow(tax_table(PHY_SP_LU_gen)))) {
    genus <- c(genus, as.character(tax_table(PHY_SP_LU_gen)[i, 6]))
    class <- c(class, as.character(tax_table(PHY_SP_LU_gen)[i, 3]))
    df <- data.frame(SUM = round(mean16S_counts_fec * sample_sums(otu_table(MGE_PHY_LU))), 
        GSUM = sample_sums(otu_table(subset_taxa(PHY_SP_LU_gen, Genus == as.character(tax_table(PHY_SP_LU_gen)[i, 
            6])))))
    xnlb <- glm.nb(SUM ~ GSUM, data = df, link = log)
    xnlb0 = glm.nb(SUM ~ 1, data = df)
    pnlb <- c(pnlb, anova(xnlb, xnlb0)[2, 8])
    pnlb2 <- c(pnlb2, coef(summary(xnlb))[2, 4])
    estimate <- c(estimate, coef(summary(xnlb))[2, 1])
}


# Get how many samples have the Genus
otu_table(PHY_SP_LU_gen)[otu_table(PHY_SP_LU_gen) > 0] <- 1
n <- rowSums(otu_table(PHY_SP_LU_gen))

# Combine into a table
genus_correlations_mgeInf_6M <- data.frame(genus, class, n, estimate, pnlb, 
    pnlb2)

# Leave out taxa, which are seen in less than 5 samples
genus_correlations_mgeInf_6M <- genus_correlations_mgeInf_6M[which(genus_correlations_mgeInf_6M$n > 
    4), ]

# Adjust p-values
genus_correlations_mgeInf_6M$padj <- p.adjust(genus_correlations_mgeInf_6M$pnlb, 
    method = "fdr")
genus_correlations_mgeInf_6M$padj2 <- p.adjust(genus_correlations_mgeInf_6M$pnlb2, 
    method = "fdr")


genus_correlations_mgeInf_6M <- genus_correlations_mgeInf_6M[which(genus_correlations_mgeInf_6M$padj2 < 
    0.1), ]


kable(genus_correlations_mgeInf_6M, caption = "6M infants, MGE genus")
```

6M infants, MGE genus

|  | genus | class | n | estimate | pnlb | pnlb2 | padj | padj2 |
| --- | --- | --- | --- | --- | --- | --- | --- | --- |
| OTU118 | Lactobacillus | Bacilli | 11 | 0.3133498 | 0.0051523 | 0.0019657 | 0.0515227 | 0.0196567 |

# Pick best taxa for modeling the total relative sum abundace

```
############## GLM negative binomial############ ARGS, Test using GML, negative binomial
############## distribution

# Subset for using ARGs without efflux pumps and regulatory genes and check
# this also
ARG_PHY_noefl_noregl <- subset_taxa(ARG_PHY, !V2 %in% c("efflux pump", "regulatory gene"))

# Save mean 16S read count in fecal samples and use for to get the
# normalized integer counts for ARGs and MGEs
mean16S_counts_fec <- mean(SSU_counts[grep("RM", row.names(SSU_counts), invert = TRUE), 
    ])

# Fecal samples and effect of different taxa, run with ARG_PHY_noefl_noregl
# as well to get results for without efflux pumps and regulatory genes, run
# with Mot_32W and Mot_1M to get results for mothers.
ARG_PHY_Inf_6M <- subset_samples(ARG_PHY, TYPE %in% c("Inf_1M", "Inf_6M"))
PHY_SP_Inf_6M <- subset_samples(PHY_SP, TYPE %in% c("Inf_1M", "Inf_6M"))
MGE_PHY_Inf_6M <- subset_samples(MGE_PHY, TYPE %in% c("Inf_1M", "Inf_6M"))

df <- data.frame(SUM = round(mean16S_counts_fec * (sample_sums(otu_table(ARG_PHY_Inf_6M)))), 
    TYPE = sample_data(ARG_PHY_Inf_6M)$TYPE, GROUP = sample_data(ARG_PHY_Inf_6M)$GROUP, 
    BFAT6MO = sample_data(ARG_PHY_Inf_6M)$BFAT6MO, PROB = sample_data(ARG_PHY_Inf_6M)$PROB, 
    ECOLI = sample_sums(subset_taxa(PHY_SP_Inf_6M, Species == "Escherichia_coli")), 
    GAMMA = sample_sums(subset_taxa(PHY_SP_Inf_6M, Class == "Gammaproteobacteria")), 
    ESC = sample_sums(subset_taxa(PHY_SP_Inf_6M, Genus == "Escherichia")), BACTEROID = sample_sums(subset_taxa(PHY_SP_Inf_6M, 
        Genus == "Bacteroides")), EGG = sample_sums(subset_taxa(PHY_SP_Inf_6M, 
        Species == "Eggerthella_unclassified")), ACTINO = sample_sums(subset_taxa(PHY_SP_Inf_6M, 
        Class == "Actinobacteria")), CLOS = sample_sums(subset_taxa(PHY_SP_Inf_6M, 
        Class == "Clostridia")), BIFIDO = sample_sums(subset_taxa(PHY_SP_Inf_6M, 
        Genus == "Bifidobacterium")), LACT = sample_sums(subset_taxa(PHY_SP_Inf_6M, 
        Genus == "Lactobacillus")), KLEB = sample_sums(subset_taxa(PHY_SP_Inf_6M, 
        Genus == "Klebsiella")))

# Change to wanted taxa and test model
fit <- glm.nb(SUM ~ ECOLI, data = df, link = log)
fit3 <- glm.nb(SUM ~ ECOLI + EGG, data = df, link = log)

# Check is there improvement between two nested models
anova(fit, fit3, test = "Chisq")
```

```
## Likelihood ratio tests of Negative Binomial Models
## 
## Response: SUM
##         Model     theta Resid. df    2 x log-lik.   Test    df LR stat.
## 1       ECOLI 0.6433823        30       -649.7493                      
## 2 ECOLI + EGG 0.6911647        29       -646.5659 1 vs 2     1 3.183473
##      Pr(Chi)
## 1           
## 2 0.07438649
```

```
# Summary gives Wald test results
summary(fit)
```

```
## 
## Call:
## glm.nb(formula = SUM ~ ECOLI, data = df, link = log, init.theta = 0.6433822544)
## 
## Deviance Residuals: 
##     Min       1Q   Median       3Q      Max  
## -2.1409  -1.2296  -0.4438   0.2724   1.8898  
## 
## Coefficients:
##             Estimate Std. Error z value Pr(>|z|)    
## (Intercept)  8.59993    0.25426  33.823  < 2e-16 ***
## ECOLI        0.06188    0.01246   4.965 6.87e-07 ***
## ---
## Signif. codes:  0 '***' 0.001 '**' 0.01 '*' 0.05 '.' 0.1 ' ' 1
## 
## (Dispersion parameter for Negative Binomial(0.6434) family taken to be 1)
## 
##     Null deviance: 60.073  on 31  degrees of freedom
## Residual deviance: 39.128  on 30  degrees of freedom
## AIC: 655.75
## 
## Number of Fisher Scoring iterations: 1
## 
## 
##               Theta:  0.643 
##           Std. Err.:  0.136 
## 
##  2 x log-likelihood:  -649.749
```

```
summary(fit3)
```

```
## 
## Call:
## glm.nb(formula = SUM ~ ECOLI + EGG, data = df, link = log, init.theta = 0.6911646667)
## 
## Deviance Residuals: 
##     Min       1Q   Median       3Q      Max  
## -2.1475  -1.0800  -0.4618   0.3568   1.7110  
## 
## Coefficients:
##             Estimate Std. Error z value Pr(>|z|)    
## (Intercept)  8.37200    0.25810  32.437  < 2e-16 ***
## ECOLI        0.05795    0.01230   4.712 2.45e-06 ***
## EGG          0.22264    0.12270   1.815   0.0696 .  
## ---
## Signif. codes:  0 '***' 0.001 '**' 0.01 '*' 0.05 '.' 0.1 ' ' 1
## 
## (Dispersion parameter for Negative Binomial(0.6912) family taken to be 1)
## 
##     Null deviance: 64.533  on 31  degrees of freedom
## Residual deviance: 38.732  on 29  degrees of freedom
## AIC: 654.57
## 
## Number of Fisher Scoring iterations: 1
## 
## 
##               Theta:  0.691 
##           Std. Err.:  0.147 
## 
##  2 x log-likelihood:  -646.566
```

```
# MGEs, Test using GML, negative binomial distribution and a Tukey post hoc
# test

# Normalized counts to 16S
df <- data.frame(SUM = round(mean16S_counts_fec * sample_sums(otu_table(MGE_PHY_Inf_6M))), 
    TYPE = sample_data(MGE_PHY_Inf_6M)$TYPE, GROUP = sample_data(MGE_PHY_Inf_6M)$GROUP, 
    BFAT6MO = sample_data(MGE_PHY_Inf_6M)$BFAT6MO, ECOLI = sample_sums(subset_taxa(PHY_SP_Inf_6M, 
        Species == "Escherichia_coli")), ENTERO = sample_sums(subset_taxa(PHY_SP_Inf_6M, 
        Genus == "Enterococcus")), STREP = sample_sums(subset_taxa(PHY_SP_Inf_6M, 
        Genus == "Streptococcus")), BIFIDO = sample_sums(subset_taxa(PHY_SP_Inf_6M, 
        Genus == "Bifidobacterium")), LACTO = sample_sums(subset_taxa(PHY_SP_Inf_6M, 
        Genus == "Lactobacillus")), GAMMA = sample_sums(subset_taxa(PHY_SP_Inf_6M, 
        Class == "Gammaproteobacteria")))


# Using glm.nb, change to wanted treatment or taxa
fit <- glm.nb(SUM ~ ENTERO, data = df, link = log)
summary(fit)
```

```
## 
## Call:
## glm.nb(formula = SUM ~ ENTERO, data = df, link = log, init.theta = 0.7308572165)
## 
## Deviance Residuals: 
##      Min        1Q    Median        3Q       Max  
## -2.33501  -1.10282  -0.66811  -0.03096   2.25920  
## 
## Coefficients:
##             Estimate Std. Error z value Pr(>|z|)    
## (Intercept)  9.99410    0.21276  46.973   <2e-16 ***
## ENTERO       0.02568    0.02269   1.132    0.258    
## ---
## Signif. codes:  0 '***' 0.001 '**' 0.01 '*' 0.05 '.' 0.1 ' ' 1
## 
## (Dispersion parameter for Negative Binomial(0.7309) family taken to be 1)
## 
##     Null deviance: 40.168  on 31  degrees of freedom
## Residual deviance: 38.445  on 30  degrees of freedom
## AIC: 710.9
## 
## Number of Fisher Scoring iterations: 1
## 
## 
##               Theta:  0.731 
##           Std. Err.:  0.156 
## 
##  2 x log-likelihood:  -704.905
```

# ARG and MGE variation in samples due to IAP and breastmilk using DESEQ

```
# MGEs and breastfeeding Inf_6M
temp_mge <- MGE_SSU_length_norm[, ] * 10^5 + 1
MGE_DSQ <- phyloseq(otu_table(temp_mge, taxa_are_rows = T), sample_data(sample_data), 
    tax_table(as.matrix(MGE_tax)))
MGE_DSQ_LU <- subset_samples(MGE_DSQ, (TYPE == "Inf_6M"))
dds_mge_lu = phyloseq_to_deseq2(MGE_DSQ_LU, ~BFAT6MO)
```

```
## converting counts to integer mode
```

```
dds_mge_lu$BFAT6MO <- relevel(dds_mge_lu$BFAT6MO, "YES")
dds_mge_lu = DESeq(dds_mge_lu, fitType = "mean", test = "Wald", betaPrior = FALSE)
```

```
## estimating size factors
```

```
## estimating dispersions
```

```
## gene-wise dispersion estimates
```

```
## mean-dispersion relationship
```

```
## final dispersion estimates
```

```
## fitting model and testing
```

```
## -- replacing outliers and refitting for 396 genes
## -- DESeq argument 'minReplicatesForReplace' = 7 
## -- original counts are preserved in counts(dds)
```

```
## estimating dispersions
```

```
## fitting model and testing
```

```
res_mge_lu = results(dds_mge_lu, cooksCutoff = FALSE)
alpha = 0.05
sigtab_mge_Inf_6M_bf = res_mge_lu[which(res_mge_lu$padj < alpha), ]
sigtab_mge_Inf_6M_bf = cbind(as(sigtab_mge_Inf_6M_bf, "data.frame"), as(tax_table(MGE_DSQ_LU)[rownames(sigtab_mge_Inf_6M_bf), 
    ], "matrix"))


otu_table(MGE_DSQ_LU)[otu_table(MGE_DSQ_LU) == 1] <- 0
otu_table(MGE_DSQ_LU)[otu_table(MGE_DSQ_LU) > 0] <- 1
n <- rowSums(otu_table(MGE_DSQ_LU))

sigtab_mge_Inf_6M_bf = merge(sigtab_mge_Inf_6M_bf, as.data.frame(n), by = 0)


kable(sigtab_mge_Inf_6M_bf, caption = "MGEs different by breastfeeding 6 months, 145/168")
```

MGEs different by breastfeeding 6 months, 145/168

| Row.names | baseMean | log2FoldChange | lfcSE | stat | pvalue | padj | V2 | V3 | n |
| --- | --- | --- | --- | --- | --- | --- | --- | --- | --- |
| 1082\_tnpA\_CP015834.1 | 36.8125 | -5.607311 | 1.682554 | -3.332618 | 0.0008603 | 0.0039567 | transposase | tnpA | 4 |
| 1121\_tnpA\_JX486125.1 | 9.8750 | -3.681814 | 1.480929 | -2.486151 | 0.0129133 | 0.0339694 | transposase | tnpA | 4 |
| 1133\_tnpA\_KR822246.1 | 51.5000 | 5.995718 | 1.389546 | 4.314875 | 0.0000160 | 0.0001533 | transposase | tnpA | 3 |
| 1146\_tnpA\_KP826704.1 | 40.3125 | 4.979723 | 1.419124 | 3.509012 | 0.0004498 | 0.0023815 | transposase | tnpA | 4 |
| 1152\_ISBf10\_NC006347 | 2.6250 | 2.906887 | 1.125050 | 2.583785 | 0.0097723 | 0.0281421 | ISBf10 | ISBf10 | 2 |
| 1155\_tnpA\_FP236830.1 | 18.1250 | 5.122088 | 1.301375 | 3.935906 | 0.0000829 | 0.0005802 | transposase | tnpA | 3 |
| 1206\_tnpA\_CP002179.1 | 84.5000 | 8.387990 | 1.374544 | 6.102380 | 0.0000000 | 0.0000001 | transposase | tnpA | 2 |
| 1229\_tnpA\_AY545598.5 | 16.6875 | 5.994344 | 1.271505 | 4.714369 | 0.0000024 | 0.0000325 | transposase | tnpA | 2 |
| 1233\_tnpA\_JN208880.1 | 441.8750 | 4.097887 | 1.513197 | 2.708098 | 0.0067670 | 0.0211725 | transposase | tnpA | 11 |
| 1242\_tnpA\_KP826710.1 | 151.0000 | 4.359827 | 1.592858 | 2.737110 | 0.0061982 | 0.0198662 | transposase | tnpA | 6 |
| 124\_repUS2\_\_repA(pBI143)\_BFU30316 | 793.1250 | -10.045923 | 2.087295 | -4.812891 | 0.0000015 | 0.0000219 | plasmid | repUS2 | 3 |
| 1260\_tnpA\_AY351675.1 | 76.0000 | -3.534578 | 1.481236 | -2.386235 | 0.0170219 | 0.0426062 | transposase | tnpA | 9 |
| 1294\_IS91\_CU928162.2 | 58.7500 | 5.031041 | 1.509917 | 3.331999 | 0.0008622 | 0.0039567 | IS91 | IS91 | 4 |
| 1300\_IS91\_FN543503.1 | 2.3125 | 2.643853 | 1.114575 | 2.372072 | 0.0176887 | 0.0440085 | IS91 | IS91 | 1 |
| 1325\_Tn916-orf8\_KC414929 | 339.8125 | 7.544269 | 1.548986 | 4.870456 | 0.0000011 | 0.0000170 | Tn916 | Tn916-orf8 | 4 |
| 1341\_Tn916-orf9\_KC414929 | 195.6250 | 7.014042 | 1.555690 | 4.508638 | 0.0000065 | 0.0000749 | Tn916 | Tn916-orf9 | 4 |
| 1356\_Tn916-orf17\_KC414929 | 671.5625 | 6.342767 | 1.450258 | 4.373545 | 0.0000122 | 0.0001262 | Tn916 | Tn916-orf17 | 10 |
| 1366\_Tn916-orf5\_KC414929 | 397.1875 | 6.470177 | 1.573495 | 4.111979 | 0.0000392 | 0.0002946 | Tn916 | Tn916-orf5 | 6 |
| 1395\_Int-Tn916\_KC414929.1 | 377.6250 | 6.212014 | 1.348441 | 4.606812 | 0.0000041 | 0.0000497 | integrase | Int-Tn916 | 11 |
| 1399\_IS91\_DQ388534.1 | 14.7500 | 3.408079 | 1.368946 | 2.489564 | 0.0127900 | 0.0338607 | IS91 | IS91 | 3 |
| 1447\_tnpA\_CP002291.1 | 116.0000 | 7.839190 | 1.364664 | 5.744411 | 0.0000000 | 0.0000003 | transposase | tnpA | 3 |
| 146\_IncHI1B(R27)\_1\_R27\_AF250878 | 2.6250 | 2.906887 | 1.125050 | 2.583785 | 0.0097723 | 0.0281421 | plasmid | IncHI1B(R27) | 1 |
| 1485\_tnpA\_KU666846.1 | 161.3125 | 5.401917 | 1.654254 | 3.265470 | 0.0010928 | 0.0047640 | transposase | tnpA | 4 |
| 1510\_tnpA\_KJ933392.1 | 382.8750 | 5.742797 | 1.700608 | 3.376908 | 0.0007331 | 0.0034799 | transposase | tnpA | 5 |
| 1532\_Tn916-orf18\_KC414929 | 698.3750 | 6.745089 | 1.341632 | 5.027525 | 0.0000005 | 0.0000093 | Tn916 | Tn916-orf18 | 12 |
| 1535\_Tn916-orf20\_KC414929 | 559.5000 | 5.824614 | 1.411533 | 4.126447 | 0.0000368 | 0.0002818 | Tn916 | Tn916-orf20 | 12 |
| 1544\_tnpA\_AF408195.1 | 38.8125 | 4.000913 | 1.461928 | 2.736737 | 0.0062052 | 0.0198662 | transposase | tnpA | 5 |
| 1622\_tnpA\_JX077110.1 | 108.0000 | -7.166556 | 1.719840 | -4.166989 | 0.0000309 | 0.0002601 | transposase | tnpA | 5 |
| 1637\_tnpA\_HM204990.1 | 2.7500 | 2.999996 | 1.128990 | 2.657240 | 0.0078783 | 0.0242818 | transposase | tnpA | 1 |
| 1644\_tnpA\_KX029332.1 | 69.1875 | 4.932894 | 1.466155 | 3.364511 | 0.0007668 | 0.0035987 | transposase | tnpA | 6 |
| 1649\_tnpA\_KT225462.1 | 33.2500 | 5.166981 | 1.365261 | 3.784612 | 0.0001539 | 0.0009490 | transposase | tnpA | 5 |
| 1660\_int3\_CAPD01000005.1 | 31.7500 | 6.954184 | 1.314591 | 5.289999 | 0.0000001 | 0.0000034 | integrase | int3 | 2 |
| 1674\_tnpA7\_HE578057.1 | 77.8750 | 6.356740 | 1.459523 | 4.355356 | 0.0000133 | 0.0001338 | transposase | tnpA7 | 4 |
| 1685\_IS91\_CP000034.1 | 2.9375 | 3.129279 | 1.134623 | 2.757991 | 0.0058158 | 0.0192154 | IS91 | IS91 | 2 |
| 170\_IncFIB(AP001918)\_1\_\_AP001918 | 110.5000 | -5.101516 | 1.789349 | -2.851047 | 0.0043576 | 0.0152105 | plasmid | IncFIB(AP001918) | 4 |
| 172\_IncFIC(FII)\_1\_\_AP001918 | 12.8750 | -4.073238 | 1.535143 | -2.653328 | 0.0079702 | 0.0243831 | plasmid | IncFIC(FII) | 4 |
| 1754\_IS91\_CP000036.1 | 4.9375 | 3.145673 | 1.205426 | 2.609593 | 0.0090650 | 0.0271293 | IS91 | IS91 | 3 |
| 1777\_tnpA\_AH009976.2 | 204.9375 | 8.161497 | 1.400781 | 5.826391 | 0.0000000 | 0.0000002 | transposase | tnpA | 5 |
| 1779\_tnpA\_KR066794.1 | 1041.5000 | 5.139519 | 1.566250 | 3.281417 | 0.0010329 | 0.0046367 | transposase | tnpA | 10 |
| 1840\_tnpA\_EU219534.1 | 70.4375 | 6.989107 | 1.419165 | 4.924802 | 0.0000008 | 0.0000134 | transposase | tnpA | 2 |
| 184\_IncX4\_1\_\_CP002895 | 2.5000 | 2.807351 | 1.120963 | 2.504410 | 0.0122656 | 0.0326818 | plasmid | IncX4 | 1 |
| 1861\_tnpA\_FJ866609.1 | 3.0000 | 3.169921 | 1.136428 | 2.789372 | 0.0052810 | 0.0175893 | transposase | tnpA | 2 |
| 1878\_tnpA\_JX442974.1 | 22.3750 | 6.434619 | 1.210471 | 5.315798 | 0.0000001 | 0.0000031 | transposase | tnpA | 2 |
| 1897\_IS91\_MNRK01000014.1 | 9.3750 | 5.108517 | 1.229550 | 4.154787 | 0.0000326 | 0.0002689 | IS91 | IS91 | 1 |
| 1899\_IS91\_AQEM01000001.1 | 3.1875 | 3.285398 | 1.141637 | 2.877795 | 0.0040047 | 0.0141361 | IS91 | IS91 | 1 |
| 1902\_tnpA\_6\_KU665641.1 | 2.6875 | 2.954193 | 1.127038 | 2.621200 | 0.0087621 | 0.0264142 | transposase | tnpA | 1 |
| 1916\_tnpA\_KC170280.1 | 70.8125 | 5.992011 | 1.416356 | 4.230583 | 0.0000233 | 0.0002088 | transposase | tnpA | 4 |
| 1924\_Tn916-orf7\_KC414929 | 365.6875 | 6.290264 | 1.543292 | 4.075873 | 0.0000458 | 0.0003381 | Tn916 | Tn916-orf7 | 7 |
| 1947\_tnpA\_JN704639.1 | 100.0000 | 4.836871 | 1.556243 | 3.108043 | 0.0018833 | 0.0075515 | transposase | tnpA | 5 |
| 1974\_tnpA\_KF295829.1 | 220.5625 | 4.400270 | 1.412312 | 3.115650 | 0.0018354 | 0.0074316 | transposase | tnpA | 11 |
| 197\_p0111\_1\_\_AP010962 | 4.9375 | 4.066084 | 1.178762 | 3.449451 | 0.0005617 | 0.0029366 | plasmid | p0111 | 1 |
| 198\_IncFIB(pECLA)\_1\_pECLA\_CP001919 | 33.4375 | 7.030655 | 1.317906 | 5.334717 | 0.0000001 | 0.0000030 | plasmid | IncFIB(pECLA) | 1 |
| 1999\_Tn916-orf13\_KC414929 | 449.3125 | 6.220689 | 1.504622 | 4.134387 | 0.0000356 | 0.0002818 | Tn916 | Tn916-orf13 | 8 |
| 200\_IncFIB(pKPHS1)\_1\_pKPHS1\_CP003223 | 3.2500 | 3.321924 | 1.143307 | 2.905540 | 0.0036662 | 0.0133995 | plasmid | IncFIB(pKPHS1) | 1 |
| 2010\_tnpA\_IS5\_like\_KR822246.1 | 99.6250 | 4.329219 | 1.635591 | 2.646883 | 0.0081237 | 0.0246699 | transposase | tnpA | 4 |
| 2014\_tnpA\_4\_KU665641.1 | 4.0000 | 3.700435 | 1.161084 | 3.187050 | 0.0014373 | 0.0060573 | transposase | tnpA | 1 |
| 2089\_tnpA\_CYEY01000102.1 | 164.2500 | -5.959703 | 1.748613 | -3.408247 | 0.0006538 | 0.0033337 | transposase | tnpA | 5 |
| 210\_IncFII(pECLA)\_1\_pECLA\_CP001919 | 24.8125 | 5.146457 | 1.383611 | 3.719585 | 0.0001996 | 0.0011773 | plasmid | IncFII(pECLA) | 3 |
| 2116\_IS91\_FN649418.1 | 517.7500 | 5.848351 | 1.650024 | 3.544405 | 0.0003935 | 0.0021669 | IS91 | IS91 | 7 |
| 2126\_IS91\_FN822745.1 | 5.2500 | 4.169919 | 1.183822 | 3.522422 | 0.0004276 | 0.0023238 | IS91 | IS91 | 2 |
| 2137\_IS91\_CU928162.2 | 25.8125 | 6.647447 | 1.301125 | 5.109001 | 0.0000003 | 0.0000067 | IS91 | IS91 | 1 |
| 2150\_IS91\_CP000036.1 | 3.0000 | 3.169921 | 1.136428 | 2.789372 | 0.0052810 | 0.0175893 | IS91 | IS91 | 1 |
| 2161\_IS91\_LETO01000030.1 | 2.8750 | 3.087459 | 1.132782 | 2.725555 | 0.0064193 | 0.0202541 | IS91 | IS91 | 1 |
| 2163\_IS91\_HG530068.1 | 183.4375 | 9.513216 | 1.418122 | 6.708318 | 0.0000000 | 0.0000000 | IS91 | IS91 | 1 |
| 217\_IncFII(pCoo)\_1\_pCoo\_CR942285 | 3.1875 | 3.285398 | 1.141637 | 2.877795 | 0.0040047 | 0.0141361 | plasmid | IncFII(pCoo) | 1 |
| 2190\_tnpA\_JN412066.1 | 255.2500 | 4.942607 | 1.716405 | 2.879628 | 0.0039814 | 0.0141361 | transposase | tnpA | 5 |
| 2192\_tnpA\_DQ303459.3 | 115.7500 | 4.774021 | 1.589711 | 3.003075 | 0.0026727 | 0.0103000 | transposase | tnpA | 5 |
| 2193\_tnpA\_CXPB01000088.1 | 4.2500 | 3.807350 | 1.166220 | 3.264693 | 0.0010958 | 0.0047640 | transposase | tnpA | 2 |
| 2198\_tnpA\_KX710093.1 | 194.3750 | 9.597102 | 1.421217 | 6.752736 | 0.0000000 | 0.0000000 | transposase | tnpA | 2 |
| 2219\_tnpA\_KJ933392.1 | 12.1875 | 5.515691 | 1.249075 | 4.415819 | 0.0000101 | 0.0001094 | transposase | tnpA | 1 |
| 2243\_tnpA1\_AY208917.2 | 4.8125 | 4.022362 | 1.176635 | 3.418530 | 0.0006296 | 0.0032503 | transposase | tnpA1 | 1 |
| 2254\_tnpA\_JNQU01000129.1 | 4.7500 | 3.999995 | 1.175548 | 3.402665 | 0.0006673 | 0.0033610 | transposase | tnpA | 1 |
| 2281\_istB\_AM261760.1 | 41.2500 | 5.102587 | 1.324180 | 3.853394 | 0.0001165 | 0.0007637 | istB | istB | 5 |
| 2284\_tnpA\_KP900016.1 | 125.5625 | 6.631565 | 1.411831 | 4.697140 | 0.0000026 | 0.0000330 | transposase | tnpA | 6 |
| 2301\_IS91\_CYDC01000057.1 | 4.0625 | 3.727915 | 1.162401 | 3.207082 | 0.0013409 | 0.0057091 | IS91 | IS91 | 1 |
| 2305\_IS91\_CP005927.1 | 38.0625 | 7.221574 | 1.326204 | 5.445296 | 0.0000001 | 0.0000018 | IS91 | IS91 | 2 |
| 2334\_tnpA\_KF753688.1 | 28.0000 | 5.285393 | 1.395451 | 3.787588 | 0.0001521 | 0.0009490 | transposase | tnpA | 3 |
| 2368\_IS91\_MNRK01000081.1 | 298.6875 | 10.218845 | 1.444110 | 7.076224 | 0.0000000 | 0.0000000 | IS91 | IS91 | 2 |
| 2369\_tnpA\_AJ431260.1 | 2.2500 | 2.584959 | 1.112360 | 2.323851 | 0.0201335 | 0.0494948 | transposase | tnpA | 1 |
| 2372\_tnpA\_JX077110.1 | 199.1875 | 6.460405 | 1.536603 | 4.204343 | 0.0000262 | 0.0002253 | transposase | tnpA | 5 |
| 2374\_tnpA\_KX029332.1 | 108.0625 | 4.225184 | 1.626256 | 2.598105 | 0.0093740 | 0.0276532 | transposase | tnpA | 5 |
| 2412\_tnpA\_AY607094.1 | 7.9375 | 4.845483 | 1.216841 | 3.982017 | 0.0000683 | 0.0004866 | transposase | tnpA | 1 |
| 2422\_IS91\_CBZU010000001.1 | 57.8750 | -6.263640 | 1.635852 | -3.828978 | 0.0001287 | 0.0008176 | IS91 | IS91 | 5 |
| 2430\_tnpA\_KF601686.2 | 126.6250 | 4.607457 | 1.678740 | 2.744592 | 0.0060586 | 0.0197024 | transposase | tnpA | 4 |
| 2442\_tnpA\_12\_KU665641.1 | 147.7500 | 5.109568 | 1.616217 | 3.161437 | 0.0015699 | 0.0064838 | transposase | tnpA | 5 |
| 2444\_tnpA\_X87814.1 | 3.3125 | 3.357548 | 1.144945 | 2.932498 | 0.0033625 | 0.0123991 | transposase | tnpA | 1 |
| 2447\_tnpA\_KJ933392.1 | 9.3125 | 5.098024 | 1.229045 | 4.147954 | 0.0000335 | 0.0002717 | transposase | tnpA | 3 |
| 2457\_tnpA\_HM440049.2 | 25.1875 | 3.403845 | 1.424987 | 2.388684 | 0.0169088 | 0.0426062 | transposase | tnpA | 5 |
| 2471\_tnpA-1\_AJ877225.1 | 2.5000 | 2.807351 | 1.120963 | 2.504410 | 0.0122656 | 0.0326818 | transposase | tnpA-1 | 1 |
| 2474\_Xis-Tn916\_KC414929.1 | 346.4375 | 6.793066 | 1.563055 | 4.346018 | 0.0000139 | 0.0001363 | Tn916 | Xis-Tn916 | 5 |
| 2499\_tnpA\_13\_CP014358.1 | 22.1250 | -4.866233 | 1.621690 | -3.000716 | 0.0026935 | 0.0103000 | transposase | tnpA | 4 |
| 2508\_IS91\_ASVO01000034.1 | 2.5000 | 2.807351 | 1.120963 | 2.504410 | 0.0122656 | 0.0326818 | IS91 | IS91 | 2 |
| 2509\_tnpA\_GU371926.1 | 1204.6250 | -5.096227 | 1.869973 | -2.725294 | 0.0064244 | 0.0202541 | transposase | tnpA | 6 |
| 2537\_tnpAIS1\_KF719970.1 | 1008.6250 | 5.526728 | 1.680529 | 3.288684 | 0.0010066 | 0.0045683 | transposase | tnpAIS1 | 8 |
| 2543\_tnpA\_AY545598.5 | 69.3125 | 8.099333 | 1.362908 | 5.942686 | 0.0000000 | 0.0000001 | transposase | tnpA | 1 |
| 2548\_tnpA\_U75371.3 | 24.1250 | -4.992448 | 1.756123 | -2.842881 | 0.0044708 | 0.0153869 | transposase | tnpA | 2 |
| 2571\_tnpA\_AIGK01000078.1 | 13.2500 | -4.115463 | 1.668580 | -2.466446 | 0.0136461 | 0.0356699 | transposase | tnpA | 2 |
| 2591\_IS91\_CP005927.1 | 79.5625 | 8.300337 | 1.371032 | 6.054081 | 0.0000000 | 0.0000001 | IS91 | IS91 | 2 |
| 2633\_Tn916-orf19\_KC414929 | 281.4375 | 5.854268 | 1.501931 | 3.897828 | 0.0000971 | 0.0006571 | Tn916 | Tn916-orf19 | 7 |
| 2634\_Tn916-orf16\_KC414929 | 736.6875 | 7.027207 | 1.361478 | 5.161454 | 0.0000002 | 0.0000056 | Tn916 | Tn916-orf16 | 12 |
| 2640\_Tn916-orf14\_KC414929 | 759.7500 | 6.725998 | 1.430719 | 4.701130 | 0.0000026 | 0.0000330 | Tn916 | Tn916-orf14 | 11 |
| 2646\_Tn916-orf15\_KC414929 | 763.8750 | 6.902410 | 1.337201 | 5.161833 | 0.0000002 | 0.0000056 | Tn916 | Tn916-orf15 | 14 |
| 2649\_IS91\_MTKG01000140.1 | 7.5625 | 4.768177 | 1.213176 | 3.930327 | 0.0000848 | 0.0005839 | IS91 | IS91 | 2 |
| 2691\_tnpA\_KJ933392.1 | 23.1250 | 6.483805 | 1.293808 | 5.011412 | 0.0000005 | 0.0000097 | transposase | tnpA | 2 |
| 2694\_IS91\_APXN01000134.1 | 10.9375 | 5.348720 | 1.241115 | 4.309610 | 0.0000164 | 0.0001533 | IS91 | IS91 | 2 |
| 2704\_tnpA(IS5)\_AJ698325.1 | 121.9375 | 4.351336 | 1.541539 | 2.822721 | 0.0047618 | 0.0161198 | transposase | tnpA(IS5) | 7 |
| 2717\_tnpA\_KM877517.1 | 56.6875 | 4.785178 | 1.415018 | 3.381707 | 0.0007204 | 0.0034594 | transposase | tnpA | 6 |
| 2741\_tnpA\_CTKN01000016.1 | 63.4375 | 4.946409 | 1.527328 | 3.238603 | 0.0012012 | 0.0051675 | transposase | tnpA | 4 |
| 2752\_tnpA\_ALWR01000001.1 | 13.2500 | -4.115464 | 1.608330 | -2.558844 | 0.0105021 | 0.0297080 | transposase | tnpA | 3 |
| 2758\_tnpA\_JNNA01000051.1 | 9.1250 | 5.066082 | 1.227509 | 4.127125 | 0.0000367 | 0.0002818 | transposase | tnpA | 1 |
| 2763\_tnpA\_GQ919187.1 | 18.2500 | 6.129273 | 1.277716 | 4.797053 | 0.0000016 | 0.0000229 | transposase | tnpA | 2 |
| 2772\_tnpA\_KF295828.1 | 565.5625 | 7.249086 | 1.646886 | 4.401694 | 0.0000107 | 0.0001137 | transposase | tnpA | 5 |
| 2802\_tnpA2\_HE578058.1 | 19.0625 | 4.397724 | 1.345113 | 3.269408 | 0.0010777 | 0.0047640 | transposase | tnpA2 | 4 |
| 289\_tnpA\_KX029332.1 | 167.0000 | 4.177481 | 1.657017 | 2.521086 | 0.0116993 | 0.0319989 | transposase | tnpA | 5 |
| 314\_tnpA\_CP002474.1 | 3.3125 | 3.357548 | 1.144945 | 2.932498 | 0.0033625 | 0.0123991 | transposase | tnpA | 1 |
| 333\_tnpA\_KP893385.1 | 727.8750 | 3.927667 | 1.596115 | 2.460767 | 0.0138640 | 0.0359065 | transposase | tnpA | 10 |
| 338\_IS91\_CP000034.1 | 108.0625 | 3.907887 | 1.636185 | 2.388414 | 0.0169213 | 0.0426062 | IS91 | IS91 | 5 |
| 397\_tnpA\_FN568063.1 | 5.4375 | 3.061397 | 1.177879 | 2.599077 | 0.0093475 | 0.0276532 | transposase | tnpA | 3 |
| 406\_IS91\_CP018710.1 | 5.6250 | 4.285396 | 1.189502 | 3.602682 | 0.0003150 | 0.0018320 | IS91 | IS91 | 1 |
| 434\_tnpA\_KC170280.1 | 52.3125 | -6.117192 | 1.577497 | -3.877783 | 0.0001054 | 0.0007022 | transposase | tnpA | 7 |
| 452\_tnpA\_AF052751.1 | 3.1250 | 3.247923 | 1.139935 | 2.849219 | 0.0043827 | 0.0152105 | transposase | tnpA | 1 |
| 466\_IS91\_CP001918.1 | 21.9375 | 6.405131 | 1.290266 | 4.964194 | 0.0000007 | 0.0000119 | IS91 | IS91 | 3 |
| 481\_tnpA\_CBTV010000136.1 | 33.0000 | 4.224853 | 1.460834 | 2.892082 | 0.0038270 | 0.0138644 | transposase | tnpA | 4 |
| 49\_rep9\_4\_repA2(pTEF2)\_AE016831 | 41.3125 | 6.445191 | 1.367570 | 4.712879 | 0.0000024 | 0.0000325 | plasmid | rep9 | 2 |
| 505\_tnpA4\_AF550679.1 | 1092.0000 | -5.615348 | 1.847333 | -3.039706 | 0.0023681 | 0.0092266 | transposase | tnpA4 | 6 |
| 524\_tnpA\_JNMB01000144.1 | 134.6250 | 9.064725 | 1.401057 | 6.469919 | 0.0000000 | 0.0000000 | transposase | tnpA | 1 |
| 530\_tnpA\_KT225462.1 | 23.0625 | -4.926775 | 1.611353 | -3.057539 | 0.0022316 | 0.0088621 | transposase | tnpA | 5 |
| 536\_tnpA\_GU371926.1 | 12.7500 | 5.584954 | 1.252346 | 4.459593 | 0.0000082 | 0.0000917 | transposase | tnpA | 2 |
| 562\_tnpA\_HE577112.1 | 390.1875 | 5.976059 | 1.584533 | 3.771496 | 0.0001623 | 0.0009856 | transposase | tnpA | 7 |
| 571\_tnpA\_KX710093.1 | 167.2500 | 3.994027 | 1.546648 | 2.582377 | 0.0098122 | 0.0281421 | transposase | tnpA | 8 |
| 574\_tnpA\_KT225462.1 | 138.1250 | 4.206035 | 1.622285 | 2.592661 | 0.0095237 | 0.0278955 | transposase | tnpA | 6 |
| 576\_ISRgn1\_AF320327 | 2.5000 | 2.807351 | 1.120963 | 2.504410 | 0.0122656 | 0.0326818 | ISRgn1 | ISRgn1 | 1 |
| 591\_tnpA03\_KJ721803.1 | 53.4375 | 3.760043 | 1.528742 | 2.459566 | 0.0139105 | 0.0359065 | transposase | tnpA03 | 5 |
| 607\_tnpA\_KM107842.1 | 3.9375 | 3.672420 | 1.159745 | 3.166575 | 0.0015425 | 0.0064347 | transposase | tnpA | 1 |
| 625\_IS91\_CP001383.1 | 198.3125 | 7.233948 | 1.587829 | 4.555875 | 0.0000052 | 0.0000616 | IS91 | IS91 | 3 |
| 626\_int2\_KF719972.1 | 35.3750 | -5.549446 | 1.638195 | -3.387536 | 0.0007052 | 0.0034394 | integrase | int2 | 4 |
| 627\_tnpA\_JN983042.1 | 380.3125 | 5.969662 | 1.684209 | 3.544491 | 0.0003934 | 0.0021669 | transposase | tnpA | 5 |
| 635\_int2\_AY509004.1 | 2.7500 | 2.999996 | 1.128990 | 2.657240 | 0.0078783 | 0.0242818 | integrase | int2 | 1 |
| 636\_tnpA\_KP893385.1 | 147.6250 | 4.004348 | 1.582675 | 2.530113 | 0.0114026 | 0.0313951 | transposase | tnpA | 7 |
| 639\_tnpA\_JF785549.1 | 79.2500 | 6.723625 | 1.363899 | 4.929710 | 0.0000008 | 0.0000134 | transposase | tnpA | 5 |
| 642\_tnpA-2\_AJ877225.1 | 197.8750 | 7.606389 | 1.506004 | 5.050711 | 0.0000004 | 0.0000087 | transposase | tnpA-2 | 4 |
| 655\_tnpA\_DQ393661.1 | 123.8750 | 7.560651 | 1.471896 | 5.136676 | 0.0000003 | 0.0000061 | transposase | tnpA | 2 |
| 691\_tnpA\_CTIT01000004.1 | 279.6875 | 5.784410 | 1.647373 | 3.511294 | 0.0004459 | 0.0023815 | transposase | tnpA | 5 |
| 698\_tnpA\_AJ311891.3 | 4.6875 | 3.977274 | 1.174444 | 3.386517 | 0.0007079 | 0.0034394 | transposase | tnpA | 2 |
| 699\_tnpA\_CYAM01000068.1 | 80.8125 | 5.488280 | 1.539487 | 3.565005 | 0.0003638 | 0.0020585 | transposase | tnpA | 3 |
| 6\_rep1\_6\_repE(pTEF1)\_AE016833 | 5.0000 | 3.075284 | 1.212991 | 2.535290 | 0.0112354 | 0.0311425 | plasmid | rep1 | 2 |
| 702\_tnpA\_DQ517526.1 | 4.5625 | 3.930733 | 1.099779 | 3.574112 | 0.0003514 | 0.0020158 | transposase | tnpA | 3 |
| 710\_tnpA\_AY894752.1 | 19.0000 | 5.574900 | 1.294984 | 4.304994 | 0.0000167 | 0.0001533 | transposase | tnpA | 2 |
| 713\_tnpA\_JN887338.1 | 3.8750 | 3.643851 | 1.158383 | 3.145637 | 0.0016573 | 0.0067767 | transposase | tnpA | 1 |
| 716\_tnpA\_KT988018.1 | 29.9375 | -5.306802 | 1.743312 | -3.044091 | 0.0023338 | 0.0091798 | transposase | tnpA | 3 |
| 747\_IS91\_HE970764.1 | 78.3125 | -6.701573 | 1.659596 | -4.038075 | 0.0000539 | 0.0003905 | IS91 | IS91 | 5 |
| 750\_tnpA\_KX276209.1 | 172.1250 | 5.448825 | 1.416296 | 3.847237 | 0.0001195 | 0.0007709 | transposase | tnpA | 9 |
| 765\_tnpA\_HE613569.1 | 10.0625 | 5.219161 | 1.234936 | 4.226261 | 0.0000238 | 0.0002088 | transposase | tnpA | 1 |
| 769\_tnpA\_KX029332.1 | 590.3750 | 4.804080 | 1.632511 | 2.942756 | 0.0032530 | 0.0122137 | transposase | tnpA | 9 |
| 772\_IS91\_CP002616.1 | 14.0000 | -4.196384 | 1.637611 | -2.562503 | 0.0103921 | 0.0295995 | IS91 | IS91 | 3 |
| 773\_IS91\_CP001918.1 | 58.4375 | 7.624653 | 1.279786 | 5.957757 | 0.0000000 | 0.0000001 | IS91 | IS91 | 4 |
| 789\_tnpA\_CP002474.1 | 4.6875 | 3.977274 | 1.174444 | 3.386517 | 0.0007079 | 0.0034394 | transposase | tnpA | 1 |
| 799\_tnpA\_FJ628167.2 | 340.3750 | 4.135595 | 1.762241 | 2.346782 | 0.0189363 | 0.0468306 | transposase | tnpA | 5 |
| 830\_tnpA\_KF537631.1 | 47.5000 | 4.194427 | 1.477840 | 2.838215 | 0.0045367 | 0.0154847 | transposase | tnpA | 5 |
| 831\_tnpA\_KF705206.1 | 110.2500 | 5.752288 | 1.542803 | 3.728466 | 0.0001926 | 0.0011531 | transposase | tnpA | 4 |
| 868\_tnpA\_JX077110.1 | 102.5000 | 3.896353 | 1.626510 | 2.395529 | 0.0165964 | 0.0423106 | transposase | tnpA | 5 |
| 872\_Tn916-orf6\_KC414929 | 132.8750 | 9.045761 | 1.400328 | 6.459745 | 0.0000000 | 0.0000000 | Tn916 | Tn916-orf6 | 2 |
| 887\_IS91\_AVWB01000057.1 | 20.6875 | -4.768167 | 1.734587 | -2.748877 | 0.0059800 | 0.0196011 | IS91 | IS91 | 3 |
| 912\_IS91\_CU928162.2 | 11.0000 | 3.258729 | 1.334774 | 2.441409 | 0.0146301 | 0.0375294 | IS91 | IS91 | 4 |
| 915\_tnpA\_AY607844.2 | 3.3750 | 3.392313 | 1.146551 | 2.958710 | 0.0030893 | 0.0117053 | transposase | tnpA | 2 |
| 930\_tnpA\_AAMK02000010.1 | 14.8750 | -4.285387 | 1.686205 | -2.541439 | 0.0110397 | 0.0308068 | transposase | tnpA | 2 |
| 973\_IS91\_CP002730.1 | 2.5625 | 2.857977 | 1.123025 | 2.544893 | 0.0109311 | 0.0307113 | IS91 | IS91 | 1 |
| 997\_tnpA\_AF342826.1 | 254.0000 | 7.720366 | 1.486817 | 5.192545 | 0.0000002 | 0.0000054 | transposase | tnpA | 5 |

```
# Make a figure with ggplot Resistance class order
x = tapply(sigtab_mge_Inf_6M_bf$log2FoldChange, sigtab_mge_Inf_6M_bf$V2, function(x) max(x))
x = sort(x, TRUE)
sigtab_mge_Inf_6M_bf$V2 = factor(as.character(sigtab_mge_Inf_6M_bf$V2), levels = names(x))
# Gene order
x = tapply(sigtab_mge_Inf_6M_bf$log2FoldChange, sigtab_mge_Inf_6M_bf$V3, function(x) max(x))
x = sort(x, TRUE)
sigtab_mge_Inf_6M_bf$V3 = factor(as.character(sigtab_mge_Inf_6M_bf$V3), levels = names(x))

# Plot
a <- ggplot(sigtab_mge_Inf_6M_bf, aes(x = V3, y = log2FoldChange, color = V2, 
    size = n)) + geom_point(alpha = 0.9) + theme(axis.text.x = element_text(angle = 90, 
    hjust = 0.95, vjust = 0.5, size = rel(0.7)), axis.text.y = element_text(size = rel(0.7)), 
    axis.title.x = element_blank(), axis.title.y = element_text(size = rel(0.6)), 
    legend.text = element_text(size = rel(0.55)), legend.title = element_text(size = rel(0.6)), 
    legend.position = c(0.15, 0.35), legend.key.width = unit(0.1, "lines"), 
    legend.key.height = unit(0.5, "lines")) + scale_color_brewer(palette = "Set3") + 
    scale_size(range = c(1, 5), breaks = c(1, 2, 3, 4, 5, 6, 7, 8, 9, 10)) + 
    labs(color = "") + guides(size = FALSE) + ylim(-10, 10) + geom_hline(yintercept = 0, 
    linetype = 2, color = "grey", alpha = 0.7) + ylab("log2 fold change\nbreastfed     not breastfed") + 
    ggtitle("Breastfeeding, six-month-old infants")

# Breastfeeding and ARGs Inf_6M Use length and 16S rRNA normalized relative
# abundances
temp_arg <- ARG_SSU_length_norm[, ] * 10^5 + 1
ARG_DSQ <- phyloseq(otu_table(temp_arg, taxa_are_rows = T), sample_data(sample_data), 
    tax_table(as.matrix(ARG_tax)))
ARG_DSQ_LU <- subset_samples(ARG_DSQ, (TYPE == "Inf_6M"))
dds_arg_lu = phyloseq_to_deseq2(ARG_DSQ_LU, ~BFAT6MO)
```

```
## converting counts to integer mode
```

```
dds_arg_lu$BFAT6MO <- relevel(dds_arg_lu$BFAT6MO, "YES")
dds_arg_lu = DESeq(dds_arg_lu, fitType = "mean", test = "Wald", betaPrior = FALSE)
```

```
## estimating size factors
```

```
## estimating dispersions
```

```
## gene-wise dispersion estimates
```

```
## mean-dispersion relationship
```

```
## final dispersion estimates
```

```
## fitting model and testing
```

```
## -- replacing outliers and refitting for 307 genes
## -- DESeq argument 'minReplicatesForReplace' = 7 
## -- original counts are preserved in counts(dds)
```

```
## estimating dispersions
```

```
## fitting model and testing
```

```
res_arg_lu = results(dds_arg_lu, cooksCutoff = FALSE)
alpha = 0.05
sigtab_arg_Inf_6M_bf = res_arg_lu[which(res_arg_lu$padj < alpha), ]

otu_table(ARG_DSQ_LU)[otu_table(ARG_DSQ_LU) == 1] <- 0
otu_table(ARG_DSQ_LU)[otu_table(ARG_DSQ_LU) > 0] <- 1
n <- rowSums(otu_table(ARG_DSQ_LU))

sigtab_arg_Inf_6M_bf = cbind(as(sigtab_arg_Inf_6M_bf, "data.frame"), as(tax_table(ARG_DSQ_LU)[rownames(sigtab_arg_Inf_6M_bf), 
    ], "matrix"))
sigtab_arg_Inf_6M_bf = merge(sigtab_arg_Inf_6M_bf, as.data.frame(n), by = 0)

kable(sigtab_arg_Inf_6M_bf, caption = "ARGs different with breastfeeding at 6 months, 104/112")
```

ARGs different with breastfeeding at 6 months, 104/112

| Row.names | baseMean | log2FoldChange | lfcSE | stat | pvalue | padj | V2 | V3 | n |
| --- | --- | --- | --- | --- | --- | --- | --- | --- | --- |
| ACT-12\_JX440355 | 19.0625 | 6.194747 | 1.241232 | 4.990804 | 0.0000006 | 0.0000104 | betalactam | ACT-12 | 1 |
| ACT-22\_KF992027 | 5.8125 | 4.339844 | 1.151702 | 3.768201 | 0.0001644 | 0.0010884 | betalactam | ACT-22 | 1 |
| ANT(6)-Ib\_KF864551.1 | 103.1875 | 7.667986 | 1.282063 | 5.980976 | 0.0000000 | 0.0000002 | aminoglycoside | ANT(6)-Ib | 4 |
| APH(3)-Ia\_BX664015 | 37.6875 | 7.207002 | 1.286366 | 5.602606 | 0.0000000 | 0.0000008 | aminoglycoside | APH(3’)-Ia | 2 |
| APH(6)-Id\_AF024602 | 4.1250 | 3.754883 | 1.122767 | 3.344313 | 0.0008249 | 0.0040949 | aminoglycoside | APH(6)-Id | 2 |
| CblA-1\_GQ343019 | 53.3750 | -6.146334 | 1.743039 | -3.526217 | 0.0004215 | 0.0022939 | betalactam | CblA-1 | 4 |
| FosA2\_EU487198 | 4.9375 | 4.066084 | 1.138068 | 3.572795 | 0.0003532 | 0.0020281 | fosfomycin | FosA2 | 1 |
| OXY-1-1\_Z30177 | 18.4375 | 3.809330 | 1.323958 | 2.877228 | 0.0040119 | 0.0137691 | betalactam | OXY-1-1 | 4 |
| OXY-1-2\_AJ871864 | 18.2500 | 4.198948 | 1.323736 | 3.172043 | 0.0015137 | 0.0063759 | betalactam | OXY-1-2 | 3 |
| OXY-1-3\_AY077482 | 14.2500 | 4.252811 | 1.306709 | 3.254598 | 0.0011355 | 0.0051750 | betalactam | OXY-1-3 | 3 |
| OXY-1-4\_AY077483 | 48.0000 | 5.171953 | 1.393037 | 3.712717 | 0.0002050 | 0.0012955 | betalactam | OXY-1-4 | 4 |
| OXY-1-5\_AY077486 | 9.2500 | 5.087456 | 1.188501 | 4.280563 | 0.0000186 | 0.0001787 | betalactam | OXY-1-5 | 2 |
| OXY-1-6\_Y17715 | 12.6250 | 4.220545 | 1.283300 | 3.288821 | 0.0010061 | 0.0048223 | betalactam | OXY-1-6 | 2 |
| OXY-1-7\_M27459 | 16.0000 | 4.196391 | 1.302378 | 3.222099 | 0.0012726 | 0.0057060 | betalactam | OXY-1-7 | 3 |
| OXY-5-1\_AJ871868 | 3.4375 | 3.426261 | 1.106934 | 3.095272 | 0.0019663 | 0.0080388 | betalactam | OXY-5-1 | 1 |
| OXY-5-2\_AJ871871 | 5.3750 | 4.209448 | 1.145213 | 3.675690 | 0.0002372 | 0.0014336 | betalactam | OXY-5-2 | 2 |
| OXY-6-3\_AJ871876 | 2.4375 | 2.754884 | 1.077156 | 2.557554 | 0.0105411 | 0.0344756 | betalactam | OXY-6-3 | 1 |
| VanXY-C\_EU151754 | 2.5000 | 2.807352 | 1.079288 | 2.601115 | 0.0092921 | 0.0307525 | vancomycin | VanXY-C | 1 |
| aadA12\_AY665771 | 4.7500 | 3.999995 | 1.134806 | 3.524828 | 0.0004238 | 0.0022939 | aminoglycoside | aadA12 | 1 |
| aadA12\_FJ381668 | 3.1250 | 3.247924 | 1.098595 | 2.956434 | 0.0031122 | 0.0113841 | aminoglycoside | aadA12 | 1 |
| aadA15\_DQ393783 | 3.1250 | 3.247924 | 1.098595 | 2.956434 | 0.0031122 | 0.0113841 | aminoglycoside | aadA15 | 1 |
| aadA17\_FJ460181 | 4.1875 | 3.781355 | 1.124060 | 3.364015 | 0.0007682 | 0.0038828 | aminoglycoside | aadA17 | 1 |
| aadA1\_FJ591054 | 7.9375 | 4.845483 | 1.176678 | 4.117936 | 0.0000382 | 0.0003428 | aminoglycoside | aadA1 | 1 |
| aadA1\_JN815078 | 11.1250 | 5.375032 | 1.202528 | 4.469776 | 0.0000078 | 0.0000989 | aminoglycoside | aadA1 | 1 |
| aadA1\_JQ414041 | 10.6250 | 5.303773 | 1.199092 | 4.423156 | 0.0000097 | 0.0001127 | aminoglycoside | aadA1 | 1 |
| aadA1\_JQ480156 | 35.8125 | 7.131845 | 1.283116 | 5.558225 | 0.0000000 | 0.0000008 | aminoglycoside | aadA1 | 1 |
| aadA1\_JX185132 | 10.6250 | 5.303773 | 1.199092 | 4.423156 | 0.0000097 | 0.0001127 | aminoglycoside | aadA1 | 1 |
| aadA1\_X02340 | 34.9375 | 5.885348 | 1.342890 | 4.382598 | 0.0000117 | 0.0001215 | aminoglycoside | aadA1 | 2 |
| aadA21\_AY171244 | 4.1875 | 3.781355 | 1.124060 | 3.364015 | 0.0007682 | 0.0038828 | aminoglycoside | aadA21 | 1 |
| aadA22\_AM261837 | 7.9375 | 4.845483 | 1.176678 | 4.117936 | 0.0000382 | 0.0003428 | aminoglycoside | aadA22 | 1 |
| aadA24\_DQ677333 | 11.8125 | 5.467598 | 1.206973 | 4.530008 | 0.0000059 | 0.0000781 | aminoglycoside | aadA24 | 1 |
| aadA\_NC010410 | 26.0625 | 6.661767 | 1.262374 | 5.277175 | 0.0000001 | 0.0000028 | aminoglycoside | aadA | 1 |
| acrA\_DQ679966 | 12.3125 | 3.557650 | 1.305650 | 2.724811 | 0.0064338 | 0.0218122 | efflux pump | acrA | 4 |
| acrA\_NC016845.1 | 29.8125 | 3.611569 | 1.364446 | 2.646913 | 0.0081230 | 0.0272073 | efflux pump | acrA | 6 |
| adeC\_CP003583 | 36.3125 | 6.394883 | 1.144076 | 5.589562 | 0.0000000 | 0.0000008 | efflux pump | adeC | 5 |
| aph(3)-Ia\_V00359 | 12.9375 | 4.202708 | 1.290194 | 3.257424 | 0.0011243 | 0.0051750 | aminoglycoside | aph(3’)-Ia | 3 |
| aph(3)-Ic\_X62115 | 39.1250 | 6.113331 | 1.344503 | 4.546908 | 0.0000054 | 0.0000762 | aminoglycoside | aph(3’)-Ic | 3 |
| cat\_M35190 | 6.6250 | 4.554583 | 1.162395 | 3.918274 | 0.0000892 | 0.0006887 | betalactam | cat | 1 |
| cepA\_FR688022 | 2.2500 | 2.584960 | 1.070548 | 2.414614 | 0.0157519 | 0.0497617 | betalactam | cepA | 1 |
| dfrA1\_AJ238350 | 5.4375 | 4.228813 | 1.146176 | 3.689497 | 0.0002247 | 0.0013881 | trimethoprim | dfrA1 | 1 |
| dfrA1\_AJ400733.1 | 9.2500 | 5.087456 | 1.188501 | 4.280563 | 0.0000186 | 0.0001787 | trimethoprim | dfrA1 | 1 |
| dfrA1\_AJ844287 | 24.1875 | 6.550736 | 1.257406 | 5.209721 | 0.0000002 | 0.0000035 | trimethoprim | dfrA1 | 1 |
| dfrA1\_JQ690541 | 7.2500 | 4.700433 | 1.169536 | 4.019058 | 0.0000584 | 0.0004778 | trimethoprim | dfrA1 | 1 |
| dfrA1\_NC010410 | 31.2500 | 6.930726 | 1.274340 | 5.438681 | 0.0000001 | 0.0000014 | trimethoprim | dfrA1 | 1 |
| dfrA1\_X00926 | 27.7500 | 6.754877 | 1.266511 | 5.333452 | 0.0000001 | 0.0000022 | trimethoprim | dfrA1 | 1 |
| dfrE\_AF028811 | 364.0000 | 6.106445 | 1.572504 | 3.883261 | 0.0001031 | 0.0007540 | trimethoprim | dfrE | 6 |
| efmA\_AB467372.1 | 5.2500 | 3.672421 | 1.144039 | 3.210048 | 0.0013271 | 0.0058562 | betalactam | efmA | 2 |
| efrA\_HG970100.1 | 396.6875 | 6.867322 | 1.567450 | 4.381206 | 0.0000118 | 0.0001215 | efflux pump | efrA | 6 |
| efrB\_HG970103.1 | 567.1875 | 9.973444 | 1.409947 | 7.073628 | 0.0000000 | 0.0000000 | efflux pump | efrB | 5 |
| emeA\_AB091338 | 213.0625 | 6.822919 | 1.501039 | 4.545464 | 0.0000055 | 0.0000762 | efflux pump | emeA | 5 |
| erm(B)\_AF368302 | 2.2500 | 2.584960 | 1.070548 | 2.414614 | 0.0157519 | 0.0497617 | MLSB | erm(B) | 1 |
| fosA\_ACZD01000244 | 3.0000 | 3.169921 | 1.095026 | 2.894837 | 0.0037936 | 0.0131826 | fosfomycin | fosA | 2 |
| fosA\_AY692231 | 195.8750 | 9.608237 | 1.382671 | 6.949039 | 0.0000000 | 0.0000000 | fosfomycin | fosA | 1 |
| fosA\_CP002889 | 8.1875 | 3.893710 | 1.217144 | 3.199054 | 0.0013788 | 0.0059891 | fosfomycin | fosA | 2 |
| fosA\_EU487198 | 5.9375 | 4.375034 | 1.153454 | 3.792984 | 0.0001488 | 0.0010160 | fosfomycin | fosA | 1 |
| lsa(A)\_AY225127 | 157.0625 | 6.607621 | 1.434538 | 4.606096 | 0.0000041 | 0.0000634 | MLSB | lsa(A) | 6 |
| lsa(A)\_AY58982 | 37.9375 | 7.216734 | 1.286786 | 5.608342 | 0.0000000 | 0.0000008 | MLSB | lsa(A) | 1 |
| lsa(A)\_AY737526 | 135.5625 | 7.790361 | 1.397906 | 5.572880 | 0.0000000 | 0.0000008 | MLSB | lsa(A) | 4 |
| lsaA\_AY587982 | 42.7500 | 6.246456 | 1.320672 | 4.729756 | 0.0000022 | 0.0000368 | betalactam | lsaA | 3 |
| mdtM\_U00096 | 114.0625 | 7.487829 | 1.261229 | 5.936930 | 0.0000000 | 0.0000002 | efflux pump | mdtM | 6 |
| mexB\_L11616 | 2.3750 | 2.700437 | 1.074991 | 2.512055 | 0.0120030 | 0.0388005 | efflux pump | mexB | 1 |
| oqxA\_EU370913 | 72.0000 | 4.623372 | 1.408725 | 3.281955 | 0.0010309 | 0.0048575 | efflux pump | oqxA | 7 |
| sat-1\_AB211124 | 197.3125 | 9.618826 | 1.383066 | 6.954714 | 0.0000000 | 0.0000000 | streptothricin | sat-1 | 1 |
| strA\_AF024602 | 4.2500 | 3.807350 | 1.125332 | 3.383312 | 0.0007162 | 0.0037565 | aminoglycoside | strA | 2 |
| strA\_AF321551 | 3.1875 | 3.285398 | 1.100328 | 2.985835 | 0.0028280 | 0.0110732 | aminoglycoside | strA | 2 |
| strB\_M96392 | 3.1250 | 3.247924 | 1.098595 | 2.956434 | 0.0031122 | 0.0113841 | aminoglycoside | strB | 2 |
| sul2\_AJ877041 | 3.0625 | 3.209449 | 1.096828 | 2.926119 | 0.0034322 | 0.0120778 | sulphonamide | sul2 | 2 |
| sul2\_FJ197818 | 3.0625 | 3.209449 | 1.096828 | 2.926119 | 0.0034322 | 0.0120778 | sulphonamide | sul2 | 2 |
| sul2\_FJ968160 | 3.1250 | 3.247924 | 1.098595 | 2.956434 | 0.0031122 | 0.0113841 | sulphonamide | sul2 | 2 |
| sul2\_GQ421466 | 3.1250 | 3.247924 | 1.098595 | 2.956434 | 0.0031122 | 0.0113841 | sulphonamide | sul2 | 2 |
| tet(40)\_AM419751 | 6.5625 | 4.539153 | 1.161627 | 3.907582 | 0.0000932 | 0.0007004 | tetracycline | tet(40) | 2 |
| tet(M)\_AM990992 | 40.3750 | 5.359142 | 1.312811 | 4.082189 | 0.0000446 | 0.0003876 | tetracycline | tet(M) | 6 |
| tet(M)\_EU182585 | 6.5000 | 3.746421 | 1.179818 | 3.175423 | 0.0014962 | 0.0063759 | tetracycline | tet(M) | 3 |
| tet(M)\_FN433596 | 28.0625 | 3.333085 | 1.383438 | 2.409278 | 0.0159841 | 0.0499279 | tetracycline | tet(M) | 5 |
| tet(M)\_FR671418 | 27.3125 | 4.603620 | 1.214248 | 3.791334 | 0.0001498 | 0.0010160 | tetracycline | tet(M) | 6 |
| tet(M)\_U08812 | 16.7500 | 4.684492 | 1.257669 | 3.724742 | 0.0001955 | 0.0012640 | tetracycline | tet(M) | 3 |
| tet(M)\_U58985 | 56.0625 | 5.336390 | 1.385701 | 3.851039 | 0.0001176 | 0.0008384 | tetracycline | tet(M) | 5 |
| tet(M)\_X04388 | 15.0625 | 3.555212 | 1.070901 | 3.319833 | 0.0009007 | 0.0043929 | tetracycline | tet(M) | 8 |
| tet(M)\_X56353 | 18.8750 | 5.185514 | 1.282822 | 4.042269 | 0.0000529 | 0.0004459 | tetracycline | tet(M) | 2 |
| tet(M)\_X90939 | 31.5000 | 4.900596 | 1.340102 | 3.656884 | 0.0002553 | 0.0015101 | tetracycline | tet(M) | 4 |
| tet(M)\_X92947 | 53.0000 | 6.956510 | 1.330531 | 5.228373 | 0.0000002 | 0.0000034 | tetracycline | tet(M) | 3 |
| tet(Q)\_L33696 | 590.5625 | -7.448299 | 1.697264 | -4.388414 | 0.0000114 | 0.0001215 | tetracycline | tet(Q) | 7 |
| tet(Q)\_U73497 | 107.3750 | -5.984032 | 1.501824 | -3.984508 | 0.0000676 | 0.0005371 | tetracycline | tet(Q) | 9 |
| tet(Q)\_Z21523 | 37.3750 | -5.629340 | 1.577006 | -3.569638 | 0.0003575 | 0.0020281 | tetracycline | tet(Q) | 6 |
| tet(W)\_FN396364 | 27.1875 | 3.824870 | 1.220302 | 3.134363 | 0.0017223 | 0.0071462 | tetracycline | tet(W) | 8 |
| tetA(P)\_L20800 | 4.7500 | 3.491849 | 1.135332 | 3.075620 | 0.0021007 | 0.0084635 | tetracycline | tetA(P) | 2 |
| tetB(P)\_010937 | 3.0000 | 3.169922 | 1.055118 | 3.004328 | 0.0026617 | 0.0105707 | tetracycline | tetB(P) | 2 |
| tetB(P)\_L20800 | 4.3125 | 3.832886 | 1.088417 | 3.521524 | 0.0004291 | 0.0022939 | tetracycline | tetB(P) | 2 |
| vgaC\_KU302801 | 92.4375 | 4.648594 | 1.579501 | 2.943077 | 0.0032497 | 0.0117326 | MLSB | vgaC | 4 |

```
# Make a figure with ggplot Resistance class order
x = tapply(sigtab_arg_Inf_6M_bf$log2FoldChange, sigtab_arg_Inf_6M_bf$V2, function(x) max(x))
x = sort(x, TRUE)
sigtab_arg_Inf_6M_bf$V2 = factor(as.character(sigtab_arg_Inf_6M_bf$V2), levels = names(x))
# Gene order
x = tapply(sigtab_arg_Inf_6M_bf$log2FoldChange, sigtab_arg_Inf_6M_bf$V3, function(x) max(x))
x = sort(x, TRUE)
sigtab_arg_Inf_6M_bf$V3 = factor(as.character(sigtab_arg_Inf_6M_bf$V3), levels = names(x))

# Plot
b <- ggplot(sigtab_arg_Inf_6M_bf, aes(x = V3, y = log2FoldChange, color = V2, 
    size = n)) + geom_point(alpha = 0.9) + theme(axis.text.x = element_text(angle = 90, 
    hjust = 0.95, vjust = 0.5, size = rel(0.7)), axis.text.y = element_text(size = rel(0.7)), 
    axis.title.x = element_blank(), axis.title.y = element_text(size = rel(0.6)), 
    legend.text = element_text(size = rel(0.55)), legend.title = element_text(size = rel(0.6)), 
    legend.position = c(0.15, 0.35), legend.key.width = unit(0.1, "lines"), 
    legend.key.height = unit(0.5, "lines")) + scale_color_brewer(palette = "Set3") + 
    scale_size(range = c(1, 5), breaks = c(1, 2, 3, 4, 5, 6, 7, 8, 9, 10)) + 
    labs(color = "") + guides(size = FALSE) + ylim(-10, 10) + geom_hline(yintercept = 0, 
    linetype = 2, color = "grey", alpha = 0.7) + ylab("log2 fold change\n      breastfed         not breastfed") + 
    ggtitle("Breastfeeding, six-month-old infants")


# Antibiotics and ARGs Inf_1M Use lenght and 16S rRNA normalized relative
# abundances
temp_arg <- ARG_SSU_length_norm[, ] * 10^5 + 1
ARG_DSQ <- phyloseq(otu_table(temp_arg, taxa_are_rows = T), sample_data(sample_data), 
    tax_table(as.matrix(ARG_tax)))
ARG_DSQ_LU <- subset_samples(ARG_DSQ, (TYPE == "Inf_1M"))
dds_arg_lu = phyloseq_to_deseq2(ARG_DSQ_LU, ~GROUP)
```

```
## converting counts to integer mode
```

```
dds_arg_lu$GROUP = relevel(dds_arg_lu$GROUP, "CON")
dds_arg_lu = DESeq(dds_arg_lu, fitType = "mean", test = "Wald", betaPrior = FALSE)
```

```
## estimating size factors
```

```
## estimating dispersions
```

```
## gene-wise dispersion estimates
```

```
## mean-dispersion relationship
```

```
## final dispersion estimates
```

```
## fitting model and testing
```

```
## -- replacing outliers and refitting for 268 genes
## -- DESeq argument 'minReplicatesForReplace' = 7 
## -- original counts are preserved in counts(dds)
```

```
## estimating dispersions
```

```
## fitting model and testing
```

```
res_arg_lu = results(dds_arg_lu, cooksCutoff = FALSE)
alpha = 0.05
sigtab_arg_Inf_1M_iap = res_arg_lu[which(res_arg_lu$padj < alpha), ]
sigtab_arg_Inf_1M_iap = cbind(as(sigtab_arg_Inf_1M_iap, "data.frame"), as(tax_table(ARG_DSQ_LU)[rownames(sigtab_arg_Inf_1M_iap), 
    ], "matrix"))

otu_table(ARG_DSQ_LU)[otu_table(ARG_DSQ_LU) == 1] <- 0
otu_table(ARG_DSQ_LU)[otu_table(ARG_DSQ_LU) > 0] <- 1
n <- rowSums(otu_table(ARG_DSQ_LU))

sigtab_arg_Inf_1M_iap = merge(sigtab_arg_Inf_1M_iap, as.data.frame(n), by = 0)
kable(sigtab_arg_Inf_1M_iap, caption = "ARGs different by IAP at 1 months, 23/32")
```

ARGs different by IAP at 1 months, 23/32

| Row.names | baseMean | log2FoldChange | lfcSE | stat | pvalue | padj | V2 | V3 | n |
| --- | --- | --- | --- | --- | --- | --- | --- | --- | --- |
| ANT(4)-Ib\_NC013342.1 | 19.3125 | 3.015823 | 1.097318 | 2.748357 | 0.0059895 | 0.0461953 | aminoglycoside | ANT(4’)-Ib | 7 |
| CblA-1\_GQ343019 | 7.0000 | -3.198774 | 1.099563 | -2.909132 | 0.0036243 | 0.0355638 | betalactam | CblA-1 | 4 |
| FosA5\_KP143090 | 5.1250 | 3.209449 | 1.169917 | 2.743314 | 0.0060822 | 0.0461953 | fosfomycin | FosA5 | 2 |
| aadD\_AF181950 | 8.7500 | 4.044388 | 1.232779 | 3.280707 | 0.0010355 | 0.0134455 | aminoglycoside | aadD | 3 |
| cfxA\_U38243 | 7.3125 | 3.768179 | 1.153204 | 3.267574 | 0.0010847 | 0.0134455 | betalactam | cfxA | 3 |
| dfrE\_AF028811 | 135.0625 | 8.072119 | 1.290076 | 6.257087 | 0.0000000 | 0.0000001 | trimethoprim | dfrE | 4 |
| dfrF\_AF028812 | 86.7500 | -7.430430 | 1.445168 | -5.141567 | 0.0000003 | 0.0000086 | trimethoprim | dfrF | 3 |
| efrA\_HG970100.1 | 133.5000 | 7.049839 | 1.203228 | 5.859103 | 0.0000000 | 0.0000004 | efflux pump | efrA | 6 |
| efrB\_HG970103.1 | 179.6875 | 5.126698 | 1.316690 | 3.893627 | 0.0000988 | 0.0022150 | efflux pump | efrB | 6 |
| emeA\_AB091338 | 97.8125 | 6.508719 | 1.179837 | 5.516627 | 0.0000000 | 0.0000018 | efflux pump | emeA | 6 |
| fosA\_EU195449 | 5.1250 | 3.209449 | 1.169917 | 2.743314 | 0.0060822 | 0.0461953 | fosfomycin | fosA | 2 |
| lsa(A)\_AY225127 | 28.0625 | 3.803912 | 1.125959 | 3.378374 | 0.0007292 | 0.0122475 | MLSB | lsa(A) | 6 |
| lsa(A)\_AY737526 | 55.3125 | 6.776422 | 1.276087 | 5.310312 | 0.0000001 | 0.0000043 | MLSB | lsa(A) | 3 |
| lsaA\_AY587982 | 4.9375 | 3.149743 | 1.121302 | 2.809006 | 0.0049695 | 0.0458946 | betalactam | lsaA | 3 |
| oqxA\_EU370913 | 139.9375 | 4.754214 | 1.458977 | 3.258593 | 0.0011197 | 0.0134455 | efflux pump | oqxA | 5 |
| oqxB\_EU370913 | 91.4375 | 3.897433 | 1.431409 | 2.722794 | 0.0064732 | 0.0461953 | efflux pump | oqxB | 5 |
| oqxB\_NC010378.1 | 109.9375 | 4.335758 | 1.473890 | 2.941711 | 0.0032640 | 0.0341637 | efflux pump | oqxB | 4 |
| tet(Q)\_Z21523 | 73.5000 | 3.396351 | 1.246125 | 2.725531 | 0.0064198 | 0.0461953 | tetracycline | tet(Q) | 8 |
| tet(W)\_AJ427421 | 8.3750 | -3.977272 | 1.157256 | -3.436811 | 0.0005886 | 0.0115514 | tetracycline | tet(W) | 4 |
| tet(W)\_DQ060146 | 8.3750 | -3.977271 | 1.227884 | -3.239127 | 0.0011990 | 0.0134455 | tetracycline | tet(W) | 3 |
| tet(W)\_FN396364 | 153.0000 | -6.149731 | 1.282157 | -4.796396 | 0.0000016 | 0.0000423 | tetracycline | tet(W) | 6 |
| tetW\_EU434751 | 6.9375 | -3.686494 | 1.097248 | -3.359763 | 0.0007801 | 0.0122475 | tetracycline | tetW | 4 |

```
# Make a figure with ggplot Resistance class order
x = tapply(sigtab_arg_Inf_1M_iap$log2FoldChange, sigtab_arg_Inf_1M_iap$V2, function(x) max(x))
x = sort(x, TRUE)
sigtab_arg_Inf_1M_iap$V2 = factor(as.character(sigtab_arg_Inf_1M_iap$V2), levels = names(x))
# Gene order
x = tapply(sigtab_arg_Inf_1M_iap$log2FoldChange, sigtab_arg_Inf_1M_iap$V3, function(x) max(x))
x = sort(x, TRUE)
sigtab_arg_Inf_1M_iap$V3 = factor(as.character(sigtab_arg_Inf_1M_iap$V3), levels = names(x))


# Plot
c <- ggplot(sigtab_arg_Inf_1M_iap, aes(x = V3, y = log2FoldChange, color = V2, 
    size = n)) + geom_point(alpha = 0.9) + theme(axis.text.x = element_text(angle = 90, 
    hjust = 0.95, vjust = 0.5, size = rel(0.7)), axis.text.y = element_text(size = rel(0.8)), 
    axis.title.x = element_blank(), axis.title.y = element_text(size = rel(0.6)), 
    legend.text = element_text(size = rel(0.55)), legend.title = element_text(size = rel(0.6)), 
    legend.position = c(0.95, 0.8), legend.key.width = unit(0.1, "lines"), legend.key.height = unit(0.5, 
        "lines")) + scale_color_brewer(palette = "Set3") + scale_size(range = c(1, 
    5), breaks = c(1, 2, 3, 4, 5, 6, 7, 8, 9, 10)) + labs(color = "") + guides(size = FALSE) + 
    ylim(-10, 10) + geom_hline(yintercept = 0, linetype = 2, color = "grey", 
    alpha = 0.7) + ylab("log2 fold change\ncontrol        IAP") + ggtitle("IAP, one-month-old infants")

# Antibiotics and ARGs Inf_6M Use length and 16S rRNA normalized relative
# abundances

temp_arg <- ARG_SSU_length_norm[, ] * 10^5 + 1
ARG_DSQ <- phyloseq(otu_table(temp_arg, taxa_are_rows = T), sample_data(sample_data), 
    tax_table(as.matrix(ARG_tax)))
ARG_DSQ_LU <- subset_samples(ARG_DSQ, (TYPE == "Inf_6M"))
dds_arg_lu = phyloseq_to_deseq2(ARG_DSQ_LU, ~GROUP)
```

```
## converting counts to integer mode
```

```
dds_arg_lu$GROUP = relevel(dds_arg_lu$GROUP, "CON")
dds_arg_lu = DESeq(dds_arg_lu, fitType = "mean", test = "Wald", betaPrior = FALSE)
```

```
## estimating size factors
```

```
## estimating dispersions
```

```
## gene-wise dispersion estimates
```

```
## mean-dispersion relationship
```

```
## final dispersion estimates
```

```
## fitting model and testing
```

```
## -- replacing outliers and refitting for 309 genes
## -- DESeq argument 'minReplicatesForReplace' = 7 
## -- original counts are preserved in counts(dds)
```

```
## estimating dispersions
```

```
## fitting model and testing
```

```
res_arg_lu = results(dds_arg_lu, cooksCutoff = FALSE)
alpha = 0.05
sigtab_arg_Inf_6M_iap = res_arg_lu[which(res_arg_lu$padj < alpha), ]
sigtab_arg_Inf_6M_iap = cbind(as(sigtab_arg_Inf_6M_iap, "data.frame"), as(tax_table(ARG_DSQ_LU)[rownames(sigtab_arg_Inf_6M_iap), 
    ], "matrix"))

otu_table(ARG_DSQ_LU)[otu_table(ARG_DSQ_LU) == 1] <- 0
otu_table(ARG_DSQ_LU)[otu_table(ARG_DSQ_LU) > 0] <- 1
n <- rowSums(otu_table(ARG_DSQ_LU))

sigtab_arg_Inf_6M_iap = merge(sigtab_arg_Inf_6M_iap, as.data.frame(n), by = 0)

kable(sigtab_arg_Inf_6M_iap, caption = "ARGs different with antibiotics at delivery at 6 months, 21/24")
```

ARGs different with antibiotics at delivery at 6 months, 21/24

| Row.names | baseMean | log2FoldChange | lfcSE | stat | pvalue | padj | V2 | V3 | n |
| --- | --- | --- | --- | --- | --- | --- | --- | --- | --- |
| AAC(6)-Ii\_L12710 | 9.3125 | 4.139545 | 1.206986 | 3.429655 | 0.0006043 | 0.0143747 | aminoglycoside | AAC(6’)-Ii | 3 |
| acrA\_NC016845.1 | 46.8125 | 4.073245 | 1.203428 | 3.384701 | 0.0007126 | 0.0143747 | efflux pump | acrA | 6 |
| dfrE\_AF028811 | 24.1875 | 4.123379 | 1.065551 | 3.869715 | 0.0001090 | 0.0058139 | trimethoprim | dfrE | 6 |
| efrA\_HG970100.1 | 23.2500 | 3.794932 | 1.122820 | 3.379821 | 0.0007253 | 0.0143747 | efflux pump | efrA | 6 |
| efrB\_HG970103.1 | 25.5000 | 4.355694 | 1.168050 | 3.729030 | 0.0001922 | 0.0069839 | efflux pump | efrB | 5 |
| emeA\_AB091338 | 13.4375 | 4.212989 | 1.102820 | 3.820195 | 0.0001333 | 0.0058139 | efflux pump | emeA | 5 |
| fosA\_ACZD01000244 | 5.3125 | 3.266783 | 1.084818 | 3.011365 | 0.0026008 | 0.0338655 | fosfomycin | fosA | 2 |
| fosA\_DQ396803 | 8.1875 | 3.942509 | 1.192692 | 3.305556 | 0.0009479 | 0.0172198 | fosfomycin | fosA | 2 |
| lsa(A)\_AY737526 | 4.9375 | 3.149744 | 1.047570 | 3.006713 | 0.0026409 | 0.0338655 | MLSB | lsa(A) | 4 |
| msr(C)\_AF313494 | 6.3125 | 3.539154 | 1.162542 | 3.044325 | 0.0023320 | 0.0338655 | efflux pump | msr(C) | 3 |
| tet(40)\_AM419751 | 6.5625 | -3.599906 | 1.167154 | -3.084344 | 0.0020400 | 0.0338655 | tetracycline | tet(40) | 2 |
| tet(40)\_FJ158002 | 12.2500 | -4.376962 | 1.118299 | -3.913947 | 0.0000908 | 0.0058139 | tetracycline | tet(40) | 4 |
| tet(M)\_FN433596 | 28.0625 | 4.146352 | 1.162397 | 3.567071 | 0.0003610 | 0.0101096 | tetracycline | tet(M) | 5 |
| tet(M)\_JN846696 | 12.8125 | 4.622046 | 1.129201 | 4.093201 | 0.0000425 | 0.0046375 | tetracycline | tet(M) | 3 |
| tet(M)\_M21136 | 16.5000 | 3.389324 | 1.121050 | 3.023348 | 0.0024999 | 0.0338655 | tetracycline | tet(M) | 5 |
| tet(O)\_Y07780 | 123.2500 | -4.403071 | 1.236852 | -3.559902 | 0.0003710 | 0.0101096 | tetracycline | tet(O) | 8 |
| vgaC\_KU302801 | 109.3125 | 5.745666 | 1.310478 | 4.384406 | 0.0000116 | 0.0025354 | MLSB | vgaC | 4 |

```
# Make a figure with ggplot Resistance class order
x = tapply(sigtab_arg_Inf_6M_iap$log2FoldChange, sigtab_arg_Inf_6M_iap$V2, function(x) max(x))
x = sort(x, TRUE)
sigtab_arg_Inf_6M_iap$V2 = factor(as.character(sigtab_arg_Inf_6M_iap$V2), levels = names(x))
# Gene order
x = tapply(sigtab_arg_Inf_6M_iap$log2FoldChange, sigtab_arg_Inf_6M_iap$V3, function(x) max(x))
x = sort(x, TRUE)
sigtab_arg_Inf_6M_iap$V3 = factor(as.character(sigtab_arg_Inf_6M_iap$V3), levels = names(x))


# Plot
d <- ggplot(sigtab_arg_Inf_6M_iap, aes(x = V3, y = log2FoldChange, color = V2, 
    size = n)) + geom_point(alpha = 0.9) + theme(axis.text.x = element_text(angle = 90, 
    hjust = 0.95, vjust = 0.5, size = rel(0.7)), axis.text.y = element_text(size = rel(0.7)), 
    axis.title.x = element_blank(), axis.title.y = element_text(size = rel(0.6)), 
    legend.text = element_text(size = rel(0.55)), legend.title = element_text(size = rel(0.6)), 
    legend.position = c(0.95, 0.8), legend.key.width = unit(0.1, "lines"), legend.key.height = unit(0.5, 
        "lines")) + scale_color_brewer(palette = "Set3") + scale_size(range = c(1, 
    5), breaks = c(1, 2, 3, 4, 5, 6, 7, 8, 9, 10)) + labs(color = "") + guides(size = FALSE) + 
    ylim(-10, 10) + geom_hline(yintercept = 0, linetype = 2, color = "grey", 
    alpha = 0.7) + ylab("log2 fold change\ncontrol        IAP") + ggtitle("IAP, six-month-old infants")

# MGEs and antibiotics Inf_1M Use lenght and 16S rRNA normalized relative
# abundances
temp_mge <- MGE_SSU_length_norm[, ] * 10^5 + 1
MGE_DSQ <- phyloseq(otu_table(temp_mge, taxa_are_rows = T), sample_data(sample_data), 
    tax_table(as.matrix(MGE_tax)))
MGE_DSQ_LU <- subset_samples(MGE_DSQ, (TYPE == "Inf_1M"))
dds_mge_lu = phyloseq_to_deseq2(MGE_DSQ_LU, ~GROUP)
```

```
## converting counts to integer mode
```

```
dds_mge_lu$GROUP <- relevel(dds_mge_lu$GROUP, "CON")
dds_mge_lu = DESeq(dds_mge_lu, fitType = "mean", test = "Wald", betaPrior = FALSE)
```

```
## estimating size factors
```

```
## estimating dispersions
```

```
## gene-wise dispersion estimates
```

```
## mean-dispersion relationship
```

```
## final dispersion estimates
```

```
## fitting model and testing
```

```
## -- replacing outliers and refitting for 454 genes
## -- DESeq argument 'minReplicatesForReplace' = 7 
## -- original counts are preserved in counts(dds)
```

```
## estimating dispersions
```

```
## fitting model and testing
```

```
res_mge_lu = results(dds_mge_lu, cooksCutoff = FALSE)
alpha = 0.05
sigtab_mge_Inf_1M_iap = res_mge_lu[which(res_mge_lu$padj < alpha), ]
sigtab_mge_Inf_1M_iap = cbind(as(sigtab_mge_Inf_1M_iap, "data.frame"), as(tax_table(MGE_DSQ_LU)[rownames(sigtab_mge_Inf_1M_iap), 
    ], "matrix"))

otu_table(MGE_DSQ_LU)[otu_table(MGE_DSQ_LU) == 1] <- 0
otu_table(MGE_DSQ_LU)[otu_table(MGE_DSQ_LU) > 0] <- 1
n <- rowSums(otu_table(MGE_DSQ_LU))

sigtab_mge_Inf_1M_iap = merge(sigtab_mge_Inf_1M_iap, as.data.frame(n), by = 0)

kable(sigtab_mge_Inf_1M_iap, caption = "ARGs different by IAP at 1 months, 22/32")
```

ARGs different by IAP at 1 months, 22/32

| Row.names | baseMean | log2FoldChange | lfcSE | stat | pvalue | padj | V2 | V3 | n |
| --- | --- | --- | --- | --- | --- | --- | --- | --- | --- |
| 1016\_tnpA\_AJ971344.1 | 56.0000 | 5.977270 | 1.347613 | 4.435452 | 0.0000092 | 0.0000953 | transposase | tnpA | 4 |
| 1017\_tnpA\_FKLR01000027.1 | 23.6875 | -5.535260 | 1.371054 | -4.037231 | 0.0000541 | 0.0003848 | transposase | tnpA | 2 |
| 1037\_tnpA\_AY849557.2 | 4.8125 | 3.108520 | 1.146412 | 2.711521 | 0.0066975 | 0.0205887 | transposase | tnpA | 3 |
| 1083\_int2\_CP002967.1 | 58.7500 | 6.864171 | 1.451099 | 4.730326 | 0.0000022 | 0.0000433 | integrase | int2 | 2 |
| 1090\_tnpA\_13\_KU665641.1 | 4.3125 | -2.930732 | 1.130869 | -2.591575 | 0.0095538 | 0.0279869 | transposase | tnpA | 3 |
| 1110\_tnpA\_GU371928.1 | 17.1250 | 4.722459 | 1.258034 | 3.753840 | 0.0001741 | 0.0010084 | transposase | tnpA | 4 |
| 1120\_tnpA\_AF535086.1 | 23.3125 | 3.799082 | 1.296227 | 2.930878 | 0.0033801 | 0.0126144 | transposase | tnpA | 5 |
| 1132\_tnpA\_AY509004.1 | 58.0625 | 5.834461 | 1.346530 | 4.332961 | 0.0000147 | 0.0001308 | transposase | tnpA | 4 |
| 1138\_tnpA\_KJ588779.1 | 11.3750 | 2.862493 | 1.196375 | 2.392639 | 0.0167277 | 0.0452738 | transposase | tnpA | 4 |
| 117\_rep22\_1\_repB(pUB110)\_X03408.1 | 7.3750 | 3.781354 | 1.250511 | 3.023847 | 0.0024958 | 0.0100236 | plasmid | rep22 | 3 |
| 118\_rep22\_2\_repU(pKKS825)\_FN377602.2 | 14.4375 | 3.479989 | 1.189752 | 2.924969 | 0.0034449 | 0.0126144 | plasmid | rep22 | 4 |
| 1193\_tnpA\_JX424614.1 | 58.6250 | 6.861072 | 1.450922 | 4.728766 | 0.0000023 | 0.0000433 | transposase | tnpA | 2 |
| 1229\_tnpA\_AY545598.5 | 397.8125 | 9.634090 | 1.595198 | 6.039431 | 0.0000000 | 0.0000001 | transposase | tnpA | 3 |
| 1233\_tnpA\_JN208880.1 | 43.6250 | 6.430443 | 1.192514 | 5.392343 | 0.0000001 | 0.0000022 | transposase | tnpA | 5 |
| 1238\_tnpA\_JX077110.1 | 44.5625 | 6.461468 | 1.427729 | 4.525696 | 0.0000060 | 0.0000681 | transposase | tnpA | 3 |
| 1242\_tnpA\_KP826710.1 | 207.9375 | 6.780045 | 1.449280 | 4.678215 | 0.0000029 | 0.0000450 | transposase | tnpA | 4 |
| 124\_repUS2\_\_repA(pBI143)\_BFU30316 | 1881.1875 | 11.555016 | 1.598695 | 7.227781 | 0.0000000 | 0.0000000 | plasmid | repUS2 | 4 |
| 1433\_tnpA\_KJ488943.1 | 36.1875 | 5.334744 | 1.305547 | 4.086214 | 0.0000438 | 0.0003211 | transposase | tnpA | 4 |
| 1478\_tnpA\_JOQR01000001.1 | 5.4375 | 3.303776 | 1.214432 | 2.720429 | 0.0065197 | 0.0203801 | transposase | tnpA | 2 |
| 1485\_tnpA\_KU666846.1 | 6.6250 | -3.614702 | 1.238083 | -2.919595 | 0.0035049 | 0.0126480 | transposase | tnpA | 2 |
| 1661\_IS91\_CP007037.1 | 7.0625 | 3.212990 | 1.139124 | 2.820580 | 0.0047937 | 0.0161301 | IS91 | IS91 | 4 |
| 1670\_IS91\_CP000034.1 | 18.1875 | 5.144649 | 1.345815 | 3.822701 | 0.0001320 | 0.0008428 | IS91 | IS91 | 3 |
| 1674\_tnpA7\_HE578057.1 | 6.3750 | -3.554581 | 1.233555 | -2.881575 | 0.0039569 | 0.0138771 | transposase | tnpA7 | 3 |
| 1700\_tnpA\_HF545435.1 | 46.8125 | 5.823112 | 1.335164 | 4.361345 | 0.0000129 | 0.0001192 | transposase | tnpA | 4 |
| 172\_IncFIC(FII)\_1\_\_AP001918 | 9.1875 | 3.776099 | 1.181804 | 3.195199 | 0.0013973 | 0.0062132 | plasmid | IncFIC(FII) | 4 |
| 1744\_ISCR1\_HG003695.1 | 32.6250 | 5.409382 | 1.304799 | 4.145758 | 0.0000339 | 0.0002811 | ISCR | ISCR1 | 4 |
| 1768\_tnpA\_FQ482074.1 | 73.1250 | 5.199348 | 1.374256 | 3.783391 | 0.0001547 | 0.0009396 | transposase | tnpA | 5 |
| 1771\_tnpA\_KR066794.1 | 23.8125 | 5.543024 | 1.155668 | 4.796383 | 0.0000016 | 0.0000366 | transposase | tnpA | 5 |
| 1779\_tnpA\_KR066794.1 | 331.5000 | 9.370672 | 1.301177 | 7.201690 | 0.0000000 | 0.0000000 | transposase | tnpA | 5 |
| 1878\_tnpA\_JX442974.1 | 41.4375 | -6.355315 | 1.421438 | -4.471046 | 0.0000078 | 0.0000843 | transposase | tnpA | 3 |
| 1972\_tnpA\_EU935738.1 | 13.2500 | 4.672417 | 1.314065 | 3.555696 | 0.0003770 | 0.0019157 | transposase | tnpA | 2 |
| 2010\_tnpA\_IS5\_like\_KR822246.1 | 5.6875 | -3.375033 | 1.166684 | -2.892842 | 0.0038177 | 0.0135802 | transposase | tnpA | 3 |
| 2012\_IS91\_CU928162.2 | 27.2500 | 5.024066 | 1.280402 | 3.923819 | 0.0000872 | 0.0006028 | IS91 | IS91 | 4 |
| 2109\_IS91\_FM179322.1 | 6.6875 | 3.629351 | 1.239183 | 2.928825 | 0.0034025 | 0.0126144 | IS91 | IS91 | 3 |
| 2116\_IS91\_FN649418.1 | 15.8125 | -4.602874 | 1.249874 | -3.682671 | 0.0002308 | 0.0012668 | IS91 | IS91 | 4 |
| 2128\_IS91\_ABKY02000010.1 | 12.7500 | 4.614702 | 1.310095 | 3.522419 | 0.0004276 | 0.0021296 | IS91 | IS91 | 2 |
| 2163\_IS91\_HG530068.1 | 8.8750 | -4.066080 | 1.271411 | -3.198084 | 0.0013834 | 0.0062132 | IS91 | IS91 | 3 |
| 2192\_tnpA\_DQ303459.3 | 7.3750 | -3.781352 | 1.192623 | -3.170619 | 0.0015211 | 0.0064197 | transposase | tnpA | 3 |
| 2204\_tnpA\_AB646744.1 | 8.6875 | 4.033417 | 1.269038 | 3.178326 | 0.0014813 | 0.0063593 | transposase | tnpA | 3 |
| 2213\_tnpA\_AF535087.1 | 17.8125 | 4.507789 | 1.195471 | 3.770722 | 0.0001628 | 0.0009650 | transposase | tnpA | 5 |
| 2235\_tnpA\_FR718867.1 | 145.5625 | -8.180507 | 1.457326 | -5.613368 | 0.0000000 | 0.0000008 | transposase | tnpA | 3 |
| 2372\_tnpA\_JX077110.1 | 72.3750 | -5.562226 | 1.357025 | -4.098838 | 0.0000415 | 0.0003211 | transposase | tnpA | 4 |
| 2374\_tnpA\_KX029332.1 | 21.5000 | -5.392305 | 1.248387 | -4.319417 | 0.0000156 | 0.0001343 | transposase | tnpA | 3 |
| 2422\_IS91\_CBZU010000001.1 | 297.5625 | 5.470622 | 1.470784 | 3.719529 | 0.0001996 | 0.0011295 | IS91 | IS91 | 5 |
| 2436\_tnpA\_X92946.1 | 4.5000 | 2.999996 | 1.136833 | 2.638908 | 0.0083174 | 0.0246919 | transposase | tnpA | 2 |
| 2452\_tnpA\_CCAE010000033.1 | 4.6250 | 3.044390 | 1.194316 | 2.549066 | 0.0108012 | 0.0309137 | transposase | tnpA | 2 |
| 2455\_tnpA\_AY509004.1 | 15.3750 | 4.417079 | 1.231347 | 3.587193 | 0.0003343 | 0.0017340 | transposase | tnpA | 4 |
| 2499\_tnpA\_13\_CP014358.1 | 128.5625 | 8.000689 | 1.370558 | 5.837541 | 0.0000000 | 0.0000003 | transposase | tnpA | 3 |
| 2508\_IS91\_ASVO01000034.1 | 7.3750 | 3.781354 | 1.250511 | 3.023847 | 0.0024958 | 0.0100236 | IS91 | IS91 | 2 |
| 2529\_tnpA\_EU402605.1 | 5.9375 | 3.442938 | 1.225087 | 2.810362 | 0.0049486 | 0.0162221 | transposase | tnpA | 3 |
| 2537\_tnpAIS1\_KF719970.1 | 7.8750 | -3.882634 | 1.258002 | -3.086350 | 0.0020263 | 0.0084091 | transposase | tnpAIS1 | 2 |
| 2548\_tnpA\_U75371.3 | 12.1875 | 4.546887 | 1.305450 | 3.483003 | 0.0004958 | 0.0024208 | transposase | tnpA | 3 |
| 2614\_tnpA\_AJ251743.1 | 3.7500 | 2.700436 | 1.114970 | 2.421981 | 0.0154362 | 0.0422374 | transposase | tnpA | 2 |
| 2633\_Tn916-orf19\_KC414929 | 23.0625 | -4.775283 | 1.220488 | -3.912602 | 0.0000913 | 0.0006145 | Tn916 | Tn916-orf19 | 5 |
| 2659\_IS91\_FM179322.1 | 9.5625 | 4.179902 | 1.279566 | 3.266656 | 0.0010883 | 0.0052111 | IS91 | IS91 | 3 |
| 2669\_tnpA\_JX442974.1 | 133.1875 | -4.066788 | 1.541384 | -2.638401 | 0.0083298 | 0.0246919 | transposase | tnpA | 4 |
| 2691\_tnpA\_KJ933392.1 | 4.3750 | 2.954192 | 1.187266 | 2.488230 | 0.0128381 | 0.0355186 | transposase | tnpA | 3 |
| 26\_rep7\_3\_CDS1(pCW7)\_J03323 | 4.6250 | 2.852439 | 1.121259 | 2.543961 | 0.0109603 | 0.0310127 | plasmid | rep7 | 4 |
| 275\_tnpA-IS26\_AJ851089.1 | 119.6250 | 6.182194 | 1.357473 | 4.554191 | 0.0000053 | 0.0000624 | transposase | tnpA-IS26 | 6 |
| 2772\_tnpA\_KF295828.1 | 15.3125 | -4.888731 | 1.328772 | -3.679136 | 0.0002340 | 0.0012668 | transposase | tnpA | 2 |
| 301\_tnpA\_AB362339.1 | 30.8125 | 3.501436 | 1.245981 | 2.810184 | 0.0049513 | 0.0162221 | transposase | tnpA | 5 |
| 305\_IS91\_CP001063.1 | 25.7500 | 5.658201 | 1.378832 | 4.103618 | 0.0000407 | 0.0003211 | IS91 | IS91 | 3 |
| 326\_IS91\_CP000034.1 | 18.8125 | 5.194747 | 1.349107 | 3.850507 | 0.0001179 | 0.0007724 | IS91 | IS91 | 3 |
| 336\_IS26\_LN794248.1 | 77.1875 | 6.936618 | 1.401897 | 4.948023 | 0.0000007 | 0.0000207 | IS26 | IS26 | 4 |
| 338\_IS91\_CP000034.1 | 4.2500 | -2.906885 | 1.129563 | -2.573461 | 0.0100687 | 0.0291524 | IS91 | IS91 | 3 |
| 397\_tnpA\_FN568063.1 | 4.6875 | 3.066085 | 1.101396 | 2.783817 | 0.0053723 | 0.0173729 | transposase | tnpA | 3 |
| 421\_tnpA\_KU167609.1 | 44.5000 | -4.230940 | 1.412911 | -2.994484 | 0.0027491 | 0.0106957 | transposase | tnpA | 4 |
| 427\_tnpA\_JF785549.1 | 47.3750 | 6.550735 | 1.432975 | 4.571423 | 0.0000048 | 0.0000603 | transposase | tnpA | 2 |
| 482\_tnpA\_FN649418.1 | 67.2500 | 7.060681 | 1.462041 | 4.829330 | 0.0000014 | 0.0000341 | transposase | tnpA | 2 |
| 505\_tnpA4\_AF550679.1 | 1586.2500 | 5.312457 | 1.648388 | 3.222820 | 0.0012694 | 0.0059636 | transposase | tnpA4 | 5 |
| 553\_tnpA\_KP826703.1 | 55.4375 | 6.779707 | 1.446260 | 4.687750 | 0.0000028 | 0.0000450 | transposase | tnpA | 2 |
| 563\_IS91\_CP001918.1 | 54.3125 | 3.944499 | 1.439091 | 2.740967 | 0.0061259 | 0.0195557 | IS91 | IS91 | 4 |
| 571\_tnpA\_KX710093.1 | 308.7500 | 4.604856 | 1.619994 | 2.842515 | 0.0044759 | 0.0152672 | transposase | tnpA | 4 |
| 574\_tnpA\_KT225462.1 | 4.5625 | -3.022362 | 1.192588 | -2.534289 | 0.0112676 | 0.0315239 | transposase | tnpA | 3 |
| 627\_tnpA\_JN983042.1 | 131.3750 | -8.032024 | 1.381797 | -5.812738 | 0.0000000 | 0.0000003 | transposase | tnpA | 3 |
| 639\_tnpA\_JF785549.1 | 91.6875 | 4.385137 | 1.491186 | 2.940704 | 0.0032747 | 0.0125445 | transposase | tnpA | 4 |
| 653\_tnpA\_KJ933392.1 | 73.5000 | 6.007485 | 1.365624 | 4.399077 | 0.0000109 | 0.0001083 | transposase | tnpA | 4 |
| 655\_tnpA\_DQ393661.1 | 7.1875 | 3.741461 | 1.247555 | 2.999036 | 0.0027084 | 0.0106957 | transposase | tnpA | 2 |
| 689\_tnpA\_KT601170.1 | 51.1250 | 6.490059 | 1.380048 | 4.702779 | 0.0000026 | 0.0000450 | transposase | tnpA | 4 |
| 691\_tnpA\_CTIT01000004.1 | 196.5625 | 8.028363 | 1.463327 | 5.486377 | 0.0000000 | 0.0000015 | transposase | tnpA | 4 |
| 696\_IS91\_AF074613.1 | 49.1250 | 6.603613 | 1.436065 | 4.598408 | 0.0000043 | 0.0000570 | IS91 | IS91 | 3 |
| 706\_tnpA\_AOEK01000039.1 | 97.4375 | 6.194512 | 1.348355 | 4.594126 | 0.0000043 | 0.0000570 | transposase | tnpA | 4 |
| 716\_tnpA\_KT988018.1 | 37.2500 | 6.199660 | 1.412154 | 4.390215 | 0.0000113 | 0.0001084 | transposase | tnpA | 2 |
| 729\_tnpA\_LC155908.1 | 49.8750 | 6.300112 | 1.363145 | 4.621748 | 0.0000038 | 0.0000557 | transposase | tnpA | 4 |
| 750\_tnpA\_KX276209.1 | 25.3750 | -5.636609 | 1.377473 | -4.091994 | 0.0000428 | 0.0003211 | transposase | tnpA | 2 |
| 769\_tnpA\_KX029332.1 | 263.3125 | 5.845831 | 1.537215 | 3.802872 | 0.0001430 | 0.0008904 | transposase | tnpA | 5 |
| 773\_IS91\_CP001918.1 | 10.2500 | -3.797948 | 1.185550 | -3.203533 | 0.0013575 | 0.0062132 | IS91 | IS91 | 4 |
| 799\_tnpA\_FJ628167.2 | 24.8750 | -4.027174 | 1.263022 | -3.188523 | 0.0014300 | 0.0062469 | transposase | tnpA | 4 |
| 868\_tnpA\_JX077110.1 | 13.8125 | 4.734701 | 1.318328 | 3.591444 | 0.0003289 | 0.0017340 | transposase | tnpA | 3 |
| 912\_IS91\_CU928162.2 | 6.1250 | 2.983509 | 1.120417 | 2.662855 | 0.0077481 | 0.0235277 | IS91 | IS91 | 4 |
| 922\_tnpA\_KP987215.1 | 219.8125 | 4.029018 | 1.481798 | 2.719007 | 0.0065478 | 0.0203801 | transposase | tnpA | 5 |
| 978\_tnpA\_AF141323.1 | 1111.2500 | 4.743146 | 1.653209 | 2.869053 | 0.0041170 | 0.0142380 | transposase | tnpA | 5 |

```
# Make a figure with ggplot Resistance class order
x = tapply(sigtab_mge_Inf_1M_iap$log2FoldChange, sigtab_mge_Inf_1M_iap$V2, function(x) max(x))
x = sort(x, TRUE)
sigtab_mge_Inf_1M_iap$V2 = factor(as.character(sigtab_mge_Inf_1M_iap$V2), levels = names(x))
# Gene order
x = tapply(sigtab_mge_Inf_1M_iap$log2FoldChange, sigtab_mge_Inf_1M_iap$V3, function(x) max(x))
x = sort(x, TRUE)
sigtab_mge_Inf_1M_iap$V3 = factor(as.character(sigtab_mge_Inf_1M_iap$V3), levels = names(x))

kable(sigtab_mge_Inf_1M_iap, caption = "ARGs different by IAP at 1 months, ")
```

ARGs different by IAP at 1 months,

| Row.names | baseMean | log2FoldChange | lfcSE | stat | pvalue | padj | V2 | V3 | n |
| --- | --- | --- | --- | --- | --- | --- | --- | --- | --- |
| 1016\_tnpA\_AJ971344.1 | 56.0000 | 5.977270 | 1.347613 | 4.435452 | 0.0000092 | 0.0000953 | transposase | tnpA | 4 |
| 1017\_tnpA\_FKLR01000027.1 | 23.6875 | -5.535260 | 1.371054 | -4.037231 | 0.0000541 | 0.0003848 | transposase | tnpA | 2 |
| 1037\_tnpA\_AY849557.2 | 4.8125 | 3.108520 | 1.146412 | 2.711521 | 0.0066975 | 0.0205887 | transposase | tnpA | 3 |
| 1083\_int2\_CP002967.1 | 58.7500 | 6.864171 | 1.451099 | 4.730326 | 0.0000022 | 0.0000433 | integrase | int2 | 2 |
| 1090\_tnpA\_13\_KU665641.1 | 4.3125 | -2.930732 | 1.130869 | -2.591575 | 0.0095538 | 0.0279869 | transposase | tnpA | 3 |
| 1110\_tnpA\_GU371928.1 | 17.1250 | 4.722459 | 1.258034 | 3.753840 | 0.0001741 | 0.0010084 | transposase | tnpA | 4 |
| 1120\_tnpA\_AF535086.1 | 23.3125 | 3.799082 | 1.296227 | 2.930878 | 0.0033801 | 0.0126144 | transposase | tnpA | 5 |
| 1132\_tnpA\_AY509004.1 | 58.0625 | 5.834461 | 1.346530 | 4.332961 | 0.0000147 | 0.0001308 | transposase | tnpA | 4 |
| 1138\_tnpA\_KJ588779.1 | 11.3750 | 2.862493 | 1.196375 | 2.392639 | 0.0167277 | 0.0452738 | transposase | tnpA | 4 |
| 117\_rep22\_1\_repB(pUB110)\_X03408.1 | 7.3750 | 3.781354 | 1.250511 | 3.023847 | 0.0024958 | 0.0100236 | plasmid | rep22 | 3 |
| 118\_rep22\_2\_repU(pKKS825)\_FN377602.2 | 14.4375 | 3.479989 | 1.189752 | 2.924969 | 0.0034449 | 0.0126144 | plasmid | rep22 | 4 |
| 1193\_tnpA\_JX424614.1 | 58.6250 | 6.861072 | 1.450922 | 4.728766 | 0.0000023 | 0.0000433 | transposase | tnpA | 2 |
| 1229\_tnpA\_AY545598.5 | 397.8125 | 9.634090 | 1.595198 | 6.039431 | 0.0000000 | 0.0000001 | transposase | tnpA | 3 |
| 1233\_tnpA\_JN208880.1 | 43.6250 | 6.430443 | 1.192514 | 5.392343 | 0.0000001 | 0.0000022 | transposase | tnpA | 5 |
| 1238\_tnpA\_JX077110.1 | 44.5625 | 6.461468 | 1.427729 | 4.525696 | 0.0000060 | 0.0000681 | transposase | tnpA | 3 |
| 1242\_tnpA\_KP826710.1 | 207.9375 | 6.780045 | 1.449280 | 4.678215 | 0.0000029 | 0.0000450 | transposase | tnpA | 4 |
| 124\_repUS2\_\_repA(pBI143)\_BFU30316 | 1881.1875 | 11.555016 | 1.598695 | 7.227781 | 0.0000000 | 0.0000000 | plasmid | repUS2 | 4 |
| 1433\_tnpA\_KJ488943.1 | 36.1875 | 5.334744 | 1.305547 | 4.086214 | 0.0000438 | 0.0003211 | transposase | tnpA | 4 |
| 1478\_tnpA\_JOQR01000001.1 | 5.4375 | 3.303776 | 1.214432 | 2.720429 | 0.0065197 | 0.0203801 | transposase | tnpA | 2 |
| 1485\_tnpA\_KU666846.1 | 6.6250 | -3.614702 | 1.238083 | -2.919595 | 0.0035049 | 0.0126480 | transposase | tnpA | 2 |
| 1661\_IS91\_CP007037.1 | 7.0625 | 3.212990 | 1.139124 | 2.820580 | 0.0047937 | 0.0161301 | IS91 | IS91 | 4 |
| 1670\_IS91\_CP000034.1 | 18.1875 | 5.144649 | 1.345815 | 3.822701 | 0.0001320 | 0.0008428 | IS91 | IS91 | 3 |
| 1674\_tnpA7\_HE578057.1 | 6.3750 | -3.554581 | 1.233555 | -2.881575 | 0.0039569 | 0.0138771 | transposase | tnpA7 | 3 |
| 1700\_tnpA\_HF545435.1 | 46.8125 | 5.823112 | 1.335164 | 4.361345 | 0.0000129 | 0.0001192 | transposase | tnpA | 4 |
| 172\_IncFIC(FII)\_1\_\_AP001918 | 9.1875 | 3.776099 | 1.181804 | 3.195199 | 0.0013973 | 0.0062132 | plasmid | IncFIC(FII) | 4 |
| 1744\_ISCR1\_HG003695.1 | 32.6250 | 5.409382 | 1.304799 | 4.145758 | 0.0000339 | 0.0002811 | ISCR | ISCR1 | 4 |
| 1768\_tnpA\_FQ482074.1 | 73.1250 | 5.199348 | 1.374256 | 3.783391 | 0.0001547 | 0.0009396 | transposase | tnpA | 5 |
| 1771\_tnpA\_KR066794.1 | 23.8125 | 5.543024 | 1.155668 | 4.796383 | 0.0000016 | 0.0000366 | transposase | tnpA | 5 |
| 1779\_tnpA\_KR066794.1 | 331.5000 | 9.370672 | 1.301177 | 7.201690 | 0.0000000 | 0.0000000 | transposase | tnpA | 5 |
| 1878\_tnpA\_JX442974.1 | 41.4375 | -6.355315 | 1.421438 | -4.471046 | 0.0000078 | 0.0000843 | transposase | tnpA | 3 |
| 1972\_tnpA\_EU935738.1 | 13.2500 | 4.672417 | 1.314065 | 3.555696 | 0.0003770 | 0.0019157 | transposase | tnpA | 2 |
| 2010\_tnpA\_IS5\_like\_KR822246.1 | 5.6875 | -3.375033 | 1.166684 | -2.892842 | 0.0038177 | 0.0135802 | transposase | tnpA | 3 |
| 2012\_IS91\_CU928162.2 | 27.2500 | 5.024066 | 1.280402 | 3.923819 | 0.0000872 | 0.0006028 | IS91 | IS91 | 4 |
| 2109\_IS91\_FM179322.1 | 6.6875 | 3.629351 | 1.239183 | 2.928825 | 0.0034025 | 0.0126144 | IS91 | IS91 | 3 |
| 2116\_IS91\_FN649418.1 | 15.8125 | -4.602874 | 1.249874 | -3.682671 | 0.0002308 | 0.0012668 | IS91 | IS91 | 4 |
| 2128\_IS91\_ABKY02000010.1 | 12.7500 | 4.614702 | 1.310095 | 3.522419 | 0.0004276 | 0.0021296 | IS91 | IS91 | 2 |
| 2163\_IS91\_HG530068.1 | 8.8750 | -4.066080 | 1.271411 | -3.198084 | 0.0013834 | 0.0062132 | IS91 | IS91 | 3 |
| 2192\_tnpA\_DQ303459.3 | 7.3750 | -3.781352 | 1.192623 | -3.170619 | 0.0015211 | 0.0064197 | transposase | tnpA | 3 |
| 2204\_tnpA\_AB646744.1 | 8.6875 | 4.033417 | 1.269038 | 3.178326 | 0.0014813 | 0.0063593 | transposase | tnpA | 3 |
| 2213\_tnpA\_AF535087.1 | 17.8125 | 4.507789 | 1.195471 | 3.770722 | 0.0001628 | 0.0009650 | transposase | tnpA | 5 |
| 2235\_tnpA\_FR718867.1 | 145.5625 | -8.180507 | 1.457326 | -5.613368 | 0.0000000 | 0.0000008 | transposase | tnpA | 3 |
| 2372\_tnpA\_JX077110.1 | 72.3750 | -5.562226 | 1.357025 | -4.098838 | 0.0000415 | 0.0003211 | transposase | tnpA | 4 |
| 2374\_tnpA\_KX029332.1 | 21.5000 | -5.392305 | 1.248387 | -4.319417 | 0.0000156 | 0.0001343 | transposase | tnpA | 3 |
| 2422\_IS91\_CBZU010000001.1 | 297.5625 | 5.470622 | 1.470784 | 3.719529 | 0.0001996 | 0.0011295 | IS91 | IS91 | 5 |
| 2436\_tnpA\_X92946.1 | 4.5000 | 2.999996 | 1.136833 | 2.638908 | 0.0083174 | 0.0246919 | transposase | tnpA | 2 |
| 2452\_tnpA\_CCAE010000033.1 | 4.6250 | 3.044390 | 1.194316 | 2.549066 | 0.0108012 | 0.0309137 | transposase | tnpA | 2 |
| 2455\_tnpA\_AY509004.1 | 15.3750 | 4.417079 | 1.231347 | 3.587193 | 0.0003343 | 0.0017340 | transposase | tnpA | 4 |
| 2499\_tnpA\_13\_CP014358.1 | 128.5625 | 8.000689 | 1.370558 | 5.837541 | 0.0000000 | 0.0000003 | transposase | tnpA | 3 |
| 2508\_IS91\_ASVO01000034.1 | 7.3750 | 3.781354 | 1.250511 | 3.023847 | 0.0024958 | 0.0100236 | IS91 | IS91 | 2 |
| 2529\_tnpA\_EU402605.1 | 5.9375 | 3.442938 | 1.225087 | 2.810362 | 0.0049486 | 0.0162221 | transposase | tnpA | 3 |
| 2537\_tnpAIS1\_KF719970.1 | 7.8750 | -3.882634 | 1.258002 | -3.086350 | 0.0020263 | 0.0084091 | transposase | tnpAIS1 | 2 |
| 2548\_tnpA\_U75371.3 | 12.1875 | 4.546887 | 1.305450 | 3.483003 | 0.0004958 | 0.0024208 | transposase | tnpA | 3 |
| 2614\_tnpA\_AJ251743.1 | 3.7500 | 2.700436 | 1.114970 | 2.421981 | 0.0154362 | 0.0422374 | transposase | tnpA | 2 |
| 2633\_Tn916-orf19\_KC414929 | 23.0625 | -4.775283 | 1.220488 | -3.912602 | 0.0000913 | 0.0006145 | Tn916 | Tn916-orf19 | 5 |
| 2659\_IS91\_FM179322.1 | 9.5625 | 4.179902 | 1.279566 | 3.266656 | 0.0010883 | 0.0052111 | IS91 | IS91 | 3 |
| 2669\_tnpA\_JX442974.1 | 133.1875 | -4.066788 | 1.541384 | -2.638401 | 0.0083298 | 0.0246919 | transposase | tnpA | 4 |
| 2691\_tnpA\_KJ933392.1 | 4.3750 | 2.954192 | 1.187266 | 2.488230 | 0.0128381 | 0.0355186 | transposase | tnpA | 3 |
| 26\_rep7\_3\_CDS1(pCW7)\_J03323 | 4.6250 | 2.852439 | 1.121259 | 2.543961 | 0.0109603 | 0.0310127 | plasmid | rep7 | 4 |
| 275\_tnpA-IS26\_AJ851089.1 | 119.6250 | 6.182194 | 1.357473 | 4.554191 | 0.0000053 | 0.0000624 | transposase | tnpA-IS26 | 6 |
| 2772\_tnpA\_KF295828.1 | 15.3125 | -4.888731 | 1.328772 | -3.679136 | 0.0002340 | 0.0012668 | transposase | tnpA | 2 |
| 301\_tnpA\_AB362339.1 | 30.8125 | 3.501436 | 1.245981 | 2.810184 | 0.0049513 | 0.0162221 | transposase | tnpA | 5 |
| 305\_IS91\_CP001063.1 | 25.7500 | 5.658201 | 1.378832 | 4.103618 | 0.0000407 | 0.0003211 | IS91 | IS91 | 3 |
| 326\_IS91\_CP000034.1 | 18.8125 | 5.194747 | 1.349107 | 3.850507 | 0.0001179 | 0.0007724 | IS91 | IS91 | 3 |
| 336\_IS26\_LN794248.1 | 77.1875 | 6.936618 | 1.401897 | 4.948023 | 0.0000007 | 0.0000207 | IS26 | IS26 | 4 |
| 338\_IS91\_CP000034.1 | 4.2500 | -2.906885 | 1.129563 | -2.573461 | 0.0100687 | 0.0291524 | IS91 | IS91 | 3 |
| 397\_tnpA\_FN568063.1 | 4.6875 | 3.066085 | 1.101396 | 2.783817 | 0.0053723 | 0.0173729 | transposase | tnpA | 3 |
| 421\_tnpA\_KU167609.1 | 44.5000 | -4.230940 | 1.412911 | -2.994484 | 0.0027491 | 0.0106957 | transposase | tnpA | 4 |
| 427\_tnpA\_JF785549.1 | 47.3750 | 6.550735 | 1.432975 | 4.571423 | 0.0000048 | 0.0000603 | transposase | tnpA | 2 |
| 482\_tnpA\_FN649418.1 | 67.2500 | 7.060681 | 1.462041 | 4.829330 | 0.0000014 | 0.0000341 | transposase | tnpA | 2 |
| 505\_tnpA4\_AF550679.1 | 1586.2500 | 5.312457 | 1.648388 | 3.222820 | 0.0012694 | 0.0059636 | transposase | tnpA4 | 5 |
| 553\_tnpA\_KP826703.1 | 55.4375 | 6.779707 | 1.446260 | 4.687750 | 0.0000028 | 0.0000450 | transposase | tnpA | 2 |
| 563\_IS91\_CP001918.1 | 54.3125 | 3.944499 | 1.439091 | 2.740967 | 0.0061259 | 0.0195557 | IS91 | IS91 | 4 |
| 571\_tnpA\_KX710093.1 | 308.7500 | 4.604856 | 1.619994 | 2.842515 | 0.0044759 | 0.0152672 | transposase | tnpA | 4 |
| 574\_tnpA\_KT225462.1 | 4.5625 | -3.022362 | 1.192588 | -2.534289 | 0.0112676 | 0.0315239 | transposase | tnpA | 3 |
| 627\_tnpA\_JN983042.1 | 131.3750 | -8.032024 | 1.381797 | -5.812738 | 0.0000000 | 0.0000003 | transposase | tnpA | 3 |
| 639\_tnpA\_JF785549.1 | 91.6875 | 4.385137 | 1.491186 | 2.940704 | 0.0032747 | 0.0125445 | transposase | tnpA | 4 |
| 653\_tnpA\_KJ933392.1 | 73.5000 | 6.007485 | 1.365624 | 4.399077 | 0.0000109 | 0.0001083 | transposase | tnpA | 4 |
| 655\_tnpA\_DQ393661.1 | 7.1875 | 3.741461 | 1.247555 | 2.999036 | 0.0027084 | 0.0106957 | transposase | tnpA | 2 |
| 689\_tnpA\_KT601170.1 | 51.1250 | 6.490059 | 1.380048 | 4.702779 | 0.0000026 | 0.0000450 | transposase | tnpA | 4 |
| 691\_tnpA\_CTIT01000004.1 | 196.5625 | 8.028363 | 1.463327 | 5.486377 | 0.0000000 | 0.0000015 | transposase | tnpA | 4 |
| 696\_IS91\_AF074613.1 | 49.1250 | 6.603613 | 1.436065 | 4.598408 | 0.0000043 | 0.0000570 | IS91 | IS91 | 3 |
| 706\_tnpA\_AOEK01000039.1 | 97.4375 | 6.194512 | 1.348355 | 4.594126 | 0.0000043 | 0.0000570 | transposase | tnpA | 4 |
| 716\_tnpA\_KT988018.1 | 37.2500 | 6.199660 | 1.412154 | 4.390215 | 0.0000113 | 0.0001084 | transposase | tnpA | 2 |
| 729\_tnpA\_LC155908.1 | 49.8750 | 6.300112 | 1.363145 | 4.621748 | 0.0000038 | 0.0000557 | transposase | tnpA | 4 |
| 750\_tnpA\_KX276209.1 | 25.3750 | -5.636609 | 1.377473 | -4.091994 | 0.0000428 | 0.0003211 | transposase | tnpA | 2 |
| 769\_tnpA\_KX029332.1 | 263.3125 | 5.845831 | 1.537215 | 3.802872 | 0.0001430 | 0.0008904 | transposase | tnpA | 5 |
| 773\_IS91\_CP001918.1 | 10.2500 | -3.797948 | 1.185550 | -3.203533 | 0.0013575 | 0.0062132 | IS91 | IS91 | 4 |
| 799\_tnpA\_FJ628167.2 | 24.8750 | -4.027174 | 1.263022 | -3.188523 | 0.0014300 | 0.0062469 | transposase | tnpA | 4 |
| 868\_tnpA\_JX077110.1 | 13.8125 | 4.734701 | 1.318328 | 3.591444 | 0.0003289 | 0.0017340 | transposase | tnpA | 3 |
| 912\_IS91\_CU928162.2 | 6.1250 | 2.983509 | 1.120417 | 2.662855 | 0.0077481 | 0.0235277 | IS91 | IS91 | 4 |
| 922\_tnpA\_KP987215.1 | 219.8125 | 4.029018 | 1.481798 | 2.719007 | 0.0065478 | 0.0203801 | transposase | tnpA | 5 |
| 978\_tnpA\_AF141323.1 | 1111.2500 | 4.743146 | 1.653209 | 2.869053 | 0.0041170 | 0.0142380 | transposase | tnpA | 5 |

```
# Plot
e <- ggplot(sigtab_mge_Inf_1M_iap, aes(x = V3, y = log2FoldChange, color = V2, 
    size = n)) + geom_point(alpha = 0.9) + theme(axis.text.x = element_text(angle = 90, 
    hjust = 0.95, vjust = 0.5, size = rel(0.7)), axis.text.y = element_text(size = rel(0.8)), 
    axis.title.x = element_blank(), axis.title.y = element_text(size = rel(0.6)), 
    legend.text = element_text(size = rel(0.55)), legend.title = element_text(size = rel(0.6)), 
    legend.position = c(0.95, 0.8), legend.key.width = unit(0.1, "lines"), legend.key.height = unit(0.5, 
        "lines")) + scale_color_brewer(palette = "Set3") + scale_size(range = c(1, 
    5), breaks = c(1, 2, 3, 4, 5, 6, 7, 8, 9, 10)) + labs(color = "") + guides(size = FALSE) + 
    ylim(-10, 10) + geom_hline(yintercept = 0, linetype = 2, color = "grey", 
    alpha = 0.7) + ylab("log2 fold change\ncontrol    IAP") + ggtitle("IAP, one-month-old infants")

# MGEs and antibiotics Inf_6M Use lenght and 16S rRNA normalized relative
# abundances
temp_mge <- MGE_SSU_length_norm[, ] * 10^5 + 1
MGE_DSQ <- phyloseq(otu_table(temp_mge, taxa_are_rows = T), sample_data(sample_data), 
    tax_table(as.matrix(MGE_tax)))
MGE_DSQ_LU <- subset_samples(MGE_DSQ, (TYPE == "Inf_6M"))
dds_mge_lu = phyloseq_to_deseq2(MGE_DSQ_LU, ~GROUP)
```

```
## converting counts to integer mode
```

```
dds_mge_lu$GROUP <- relevel(dds_mge_lu$GROUP, "CON")
dds_mge_lu = DESeq(dds_mge_lu, fitType = "mean", test = "Wald", betaPrior = FALSE)
```

```
## estimating size factors
```

```
## estimating dispersions
```

```
## gene-wise dispersion estimates
```

```
## mean-dispersion relationship
```

```
## final dispersion estimates
```

```
## fitting model and testing
```

```
## -- replacing outliers and refitting for 380 genes
## -- DESeq argument 'minReplicatesForReplace' = 7 
## -- original counts are preserved in counts(dds)
```

```
## estimating dispersions
```

```
## fitting model and testing
```

```
res_mge_lu = results(dds_mge_lu, cooksCutoff = FALSE)
alpha = 0.05
sigtab_mge_Inf_6M_iap = res_mge_lu[which(res_mge_lu$padj < alpha), ]
sigtab_mge_Inf_6M_iap = cbind(as(sigtab_mge_Inf_6M_iap, "data.frame"), as(tax_table(MGE_DSQ_LU)[rownames(sigtab_mge_Inf_6M_iap), 
    ], "matrix"))

otu_table(MGE_DSQ_LU)[otu_table(MGE_DSQ_LU) == 1] <- 0
otu_table(MGE_DSQ_LU)[otu_table(MGE_DSQ_LU) > 0] <- 1
n <- rowSums(otu_table(MGE_DSQ_LU))

sigtab_mge_Inf_6M_iap = merge(sigtab_mge_Inf_6M_iap, as.data.frame(n), by = 0)
kable(sigtab_mge_Inf_6M_iap, caption = "MGEs different by IAP at 6 months, 85/103")
```

MGEs different by IAP at 6 months, 85/103

| Row.names | baseMean | log2FoldChange | lfcSE | stat | pvalue | padj | V2 | V3 | n |
| --- | --- | --- | --- | --- | --- | --- | --- | --- | --- |
| 1020\_IS91\_LFAQ01000019.1 | 5.2500 | 3.247923 | 1.120729 | 2.898046 | 0.0037550 | 0.0248154 | IS91 | IS91 | 2 |
| 1082\_tnpA\_CP015834.1 | 79.1250 | 4.696887 | 1.341145 | 3.502148 | 0.0004615 | 0.0051633 | transposase | tnpA | 4 |
| 1098\_tnpA\_KX756453.1 | 4.1250 | 2.857978 | 1.092326 | 2.616416 | 0.0088858 | 0.0439545 | transposase | tnpA | 3 |
| 1193\_tnpA\_JX424614.1 | 897.4375 | 5.252673 | 1.448298 | 3.626792 | 0.0002870 | 0.0035425 | transposase | tnpA | 8 |
| 1238\_tnpA\_JX077110.1 | 364.3125 | 9.507034 | 1.339011 | 7.100041 | 0.0000000 | 0.0000000 | transposase | tnpA | 4 |
| 1242\_tnpA\_KP826710.1 | 14.6250 | 3.419536 | 1.055352 | 3.240185 | 0.0011945 | 0.0109651 | transposase | tnpA | 6 |
| 124\_repUS2\_\_repA(pBI143)\_BFU30316 | 58.1875 | 6.850173 | 1.417472 | 4.832670 | 0.0000013 | 0.0000965 | plasmid | repUS2 | 3 |
| 1325\_Tn916-orf8\_KC414929 | 25.1250 | 4.792551 | 1.234068 | 3.883540 | 0.0001029 | 0.0018427 | Tn916 | Tn916-orf8 | 4 |
| 1341\_Tn916-orf9\_KC414929 | 23.5000 | 3.811466 | 1.312623 | 2.903702 | 0.0036878 | 0.0248154 | Tn916 | Tn916-orf9 | 4 |
| 1399\_IS91\_DQ388534.1 | 5.1250 | -3.209448 | 1.118772 | -2.868724 | 0.0041213 | 0.0257224 | IS91 | IS91 | 3 |
| 1431\_IS91\_AIEX01000012.1 | 181.8750 | 4.683537 | 1.351402 | 3.465688 | 0.0005289 | 0.0054096 | IS91 | IS91 | 6 |
| 1447\_tnpA\_CP002291.1 | 4.4375 | 2.977276 | 1.108794 | 2.685147 | 0.0072498 | 0.0387377 | transposase | tnpA | 3 |
| 1478\_tnpA\_JOQR01000001.1 | 13.1250 | 3.807350 | 1.153876 | 3.299618 | 0.0009682 | 0.0091211 | transposase | tnpA | 4 |
| 1485\_tnpA\_KU666846.1 | 11.0625 | -4.061768 | 1.171545 | -3.467019 | 0.0005263 | 0.0054096 | transposase | tnpA | 4 |
| 1533\_tnpA\_FAVL01000073.1 | 70.3750 | -4.586282 | 1.421493 | -3.226384 | 0.0012536 | 0.0112202 | transposase | tnpA | 4 |
| 1670\_IS91\_CP000034.1 | 8.0625 | 3.918857 | 1.228187 | 3.190765 | 0.0014190 | 0.0120950 | IS91 | IS91 | 3 |
| 1700\_tnpA\_HF545435.1 | 4.7500 | 2.562934 | 1.004201 | 2.552211 | 0.0107042 | 0.0497674 | transposase | tnpA | 5 |
| 1716\_tnpA\_AY821803.1 | 5.9375 | 3.442939 | 1.134841 | 3.033852 | 0.0024145 | 0.0180083 | transposase | tnpA | 3 |
| 1803\_int3\_FR775240.1 | 4.4375 | -2.977275 | 1.102611 | -2.700205 | 0.0069297 | 0.0375882 | integrase | int3 | 2 |
| 1874\_tnpA\_JX843238.1 | 85.7500 | 3.849569 | 1.267626 | 3.036834 | 0.0023908 | 0.0180083 | transposase | tnpA | 8 |
| 189\_IncFIB(K)\_1\_Kpn3\_JN233704 | 9.8125 | 3.877739 | 1.159035 | 3.345661 | 0.0008209 | 0.0079424 | plasmid | IncFIB(K) | 4 |
| 1974\_tnpA\_KF295829.1 | 220.5625 | 4.415177 | 1.243356 | 3.551016 | 0.0003837 | 0.0044317 | transposase | tnpA | 11 |
| 1979\_tnpA\_JX077110.1 | 64.3125 | 3.122264 | 1.220918 | 2.557308 | 0.0105486 | 0.0496893 | transposase | tnpA | 9 |
| 1982\_tnpA\_KP987218.1 | 6.2500 | -3.337864 | 1.080129 | -3.090247 | 0.0019999 | 0.0159103 | transposase | tnpA | 4 |
| 2010\_tnpA\_IS5\_like\_KR822246.1 | 21.8125 | -4.379369 | 1.183256 | -3.701118 | 0.0002147 | 0.0030738 | transposase | tnpA | 4 |
| 2081\_tnpA\_JX077110.1 | 65.3125 | 4.408303 | 1.208473 | 3.647828 | 0.0002645 | 0.0034091 | transposase | tnpA | 7 |
| 2089\_tnpA\_CYEY01000102.1 | 145.0000 | 6.071137 | 1.364741 | 4.448565 | 0.0000086 | 0.0002579 | transposase | tnpA | 5 |
| 2128\_IS91\_ABKY02000010.1 | 10.6250 | 4.339845 | 1.083431 | 4.005651 | 0.0000618 | 0.0012301 | IS91 | IS91 | 4 |
| 2204\_tnpA\_AB646744.1 | 4.5625 | 3.022364 | 1.159867 | 2.605786 | 0.0091664 | 0.0443455 | transposase | tnpA | 3 |
| 2257\_tnpA-IS683\_KP347127.1 | 6.8125 | -3.658204 | 1.208802 | -3.026305 | 0.0024756 | 0.0180872 | transposase | tnpA-IS683 | 3 |
| 2281\_istB\_AM261760.1 | 39.5000 | 5.464100 | 1.235398 | 4.422947 | 0.0000097 | 0.0002681 | istB | istB | 5 |
| 2408\_int2\_AE017042.1 | 589.7500 | 3.558991 | 1.353583 | 2.629310 | 0.0085558 | 0.0437569 | integrase | int2 | 10 |
| 2415\_tnpA\_7\_KU665642.1 | 4.8125 | 3.108521 | 1.111493 | 2.796707 | 0.0051626 | 0.0308037 | transposase | tnpA | 3 |
| 2430\_tnpA\_KF601686.2 | 125.0625 | 7.637360 | 1.388347 | 5.501047 | 0.0000000 | 0.0000045 | transposase | tnpA | 4 |
| 2447\_tnpA\_KJ933392.1 | 5.8125 | 3.409386 | 1.189925 | 2.865211 | 0.0041673 | 0.0257224 | transposase | tnpA | 3 |
| 2486\_IS91\_FKHA01000014.1 | 39.8125 | 6.296905 | 1.335980 | 4.713323 | 0.0000024 | 0.0001246 | IS91 | IS91 | 3 |
| 2499\_tnpA\_13\_CP014358.1 | 20.3125 | 4.835182 | 1.228146 | 3.936976 | 0.0000825 | 0.0015548 | transposase | tnpA | 4 |
| 2508\_IS91\_ASVO01000034.1 | 4.8750 | 3.129279 | 1.113114 | 2.811284 | 0.0049344 | 0.0299411 | IS91 | IS91 | 2 |
| 2529\_tnpA\_EU402605.1 | 19.1875 | 4.619602 | 1.215986 | 3.799059 | 0.0001452 | 0.0023742 | transposase | tnpA | 4 |
| 2549\_tnpA\_AIGS01000013.1 | 73.1250 | 4.677553 | 1.346145 | 3.474778 | 0.0005113 | 0.0054096 | transposase | tnpA | 4 |
| 2562\_tnpA\_JX448550.1 | 6.3125 | 3.539154 | 1.199815 | 2.949750 | 0.0031803 | 0.0223246 | transposase | tnpA | 3 |
| 2571\_tnpA\_AIGK01000078.1 | 13.2500 | 4.672418 | 1.281613 | 3.645733 | 0.0002666 | 0.0034091 | transposase | tnpA | 2 |
| 2603\_tnpA\_JX077110.1 | 40.1250 | 5.842055 | 1.271484 | 4.594674 | 0.0000043 | 0.0001724 | transposase | tnpA | 5 |
| 2649\_IS91\_MTKG01000140.1 | 7.5625 | 3.820173 | 1.220899 | 3.128984 | 0.0017541 | 0.0146040 | IS91 | IS91 | 2 |
| 2668\_tnpA\_KP893385.1 | 31.1875 | 5.939570 | 1.252668 | 4.741537 | 0.0000021 | 0.0001246 | transposase | tnpA | 3 |
| 2691\_tnpA\_KJ933392.1 | 23.1250 | 5.499836 | 1.336221 | 4.115963 | 0.0000386 | 0.0008627 | transposase | tnpA | 2 |
| 2704\_tnpA(IS5)\_AJ698325.1 | 16.5000 | 2.909988 | 1.070471 | 2.718419 | 0.0065595 | 0.0366921 | transposase | tnpA(IS5) | 7 |
| 2709\_tnpA\_AGBU01000010.1 | 5.6250 | 2.844346 | 1.075830 | 2.643861 | 0.0081966 | 0.0431529 | transposase | tnpA | 4 |
| 2752\_tnpA\_ALWR01000001.1 | 23.1250 | -5.499835 | 1.207754 | -4.553771 | 0.0000053 | 0.0001886 | transposase | tnpA | 3 |
| 336\_IS26\_LN794248.1 | 7.3750 | 3.781355 | 1.100225 | 3.436894 | 0.0005884 | 0.0058516 | IS26 | IS26 | 4 |
| 338\_IS91\_CP000034.1 | 150.8125 | 5.449065 | 1.347645 | 4.043397 | 0.0000527 | 0.0011094 | IS91 | IS91 | 5 |
| 386\_tnpA\_JX077110.1 | 692.8125 | 7.875591 | 1.297481 | 6.069909 | 0.0000000 | 0.0000002 | transposase | tnpA | 8 |
| 419\_intI1\_FJ790886.1 | 4.1250 | 2.857978 | 1.093557 | 2.613470 | 0.0089628 | 0.0439545 | integrase | intI1 | 2 |
| 427\_tnpA\_JF785549.1 | 52.9375 | 3.637426 | 1.332398 | 2.729985 | 0.0063337 | 0.0365721 | transposase | tnpA | 4 |
| 428\_tnpA\_JX077110.1 | 292.1875 | 4.233373 | 1.365989 | 3.099127 | 0.0019409 | 0.0157920 | transposase | tnpA | 7 |
| 482\_tnpA\_FN649418.1 | 5.8750 | 3.426260 | 1.191217 | 2.876269 | 0.0040241 | 0.0257224 | transposase | tnpA | 3 |
| 550\_tnpA\_11\_KU665641.1 | 12.1250 | 3.289503 | 1.129970 | 2.911142 | 0.0036011 | 0.0247922 | transposase | tnpA | 5 |
| 612\_IS91\_CP000036.1 | 4.4375 | -2.977275 | 1.101174 | -2.703728 | 0.0068566 | 0.0375882 | IS91 | IS91 | 3 |
| 636\_tnpA\_KP893385.1 | 136.9375 | 4.200797 | 1.381818 | 3.040050 | 0.0023654 | 0.0180083 | transposase | tnpA | 7 |
| 639\_tnpA\_JF785549.1 | 11.7500 | 4.008169 | 1.126736 | 3.557328 | 0.0003746 | 0.0044317 | transposase | tnpA | 5 |
| 653\_tnpA\_KJ933392.1 | 6.5625 | 3.599908 | 1.204408 | 2.988944 | 0.0027994 | 0.0200439 | transposase | tnpA | 3 |
| 665\_tnpA\_AKND01000034.1 | 45.4375 | -5.779327 | 1.250063 | -4.623229 | 0.0000038 | 0.0001691 | transposase | tnpA | 4 |
| 696\_IS91\_AF074613.1 | 13.7500 | 4.727913 | 1.244861 | 3.797944 | 0.0001459 | 0.0023742 | IS91 | IS91 | 3 |
| 702\_tnpA\_DQ517526.1 | 4.5625 | 3.022364 | 1.111404 | 2.719412 | 0.0065398 | 0.0366921 | transposase | tnpA | 3 |
| 706\_tnpA\_AOEK01000039.1 | 31.1875 | 5.939569 | 1.315390 | 4.515444 | 0.0000063 | 0.0002056 | transposase | tnpA | 3 |
| 716\_tnpA\_KT988018.1 | 28.4375 | 5.804120 | 1.355363 | 4.282336 | 0.0000185 | 0.0004729 | transposase | tnpA | 3 |
| 725\_istA2\_AM261760.1 | 118.3125 | 5.861855 | 1.196527 | 4.899060 | 0.0000010 | 0.0000862 | istA2 | istA2 | 7 |
| 764\_int2\_LN614756.1 | 4.2500 | -2.906886 | 1.051830 | -2.763647 | 0.0057159 | 0.0335459 | integrase | int2 | 3 |
| 772\_IS91\_CP002616.1 | 14.0000 | 3.273014 | 1.273472 | 2.570151 | 0.0101654 | 0.0485230 | IS91 | IS91 | 3 |
| 773\_IS91\_CP001918.1 | 19.8125 | 3.425018 | 1.299025 | 2.636606 | 0.0083740 | 0.0434478 | IS91 | IS91 | 4 |
| 851\_tnpA\_CTJA01000058.1 | 7.0000 | -3.700433 | 1.153100 | -3.209118 | 0.0013314 | 0.0116256 | transposase | tnpA | 3 |
| 868\_tnpA\_JX077110.1 | 194.9375 | 5.671946 | 1.372571 | 4.132351 | 0.0000359 | 0.0008570 | transposase | tnpA | 5 |
| 872\_Tn916-orf6\_KC414929 | 14.3750 | 4.794408 | 1.289933 | 3.716788 | 0.0002018 | 0.0030098 | Tn916 | Tn916-orf6 | 2 |
| 921\_tnpAB\_EU402605.1 | 139.6875 | 3.453104 | 1.318721 | 2.618524 | 0.0088311 | 0.0439545 | transposase | tnpAB | 7 |
| 922\_tnpA\_KP987215.1 | 76.6875 | 4.386166 | 1.201461 | 3.650694 | 0.0002615 | 0.0034091 | transposase | tnpA | 7 |
| 930\_tnpA\_AAMK02000010.1 | 14.8750 | 4.845482 | 1.293389 | 3.746345 | 0.0001794 | 0.0027929 | transposase | tnpA | 2 |
| 978\_tnpA\_AF141323.1 | 842.3125 | 3.885674 | 1.343000 | 2.893279 | 0.0038124 | 0.0248154 | transposase | tnpA | 10 |

```
# Make a figure with ggplot Resistance class order
x = tapply(sigtab_mge_Inf_6M_iap$log2FoldChange, sigtab_mge_Inf_6M_iap$V2, function(x) max(x))
x = sort(x, TRUE)
sigtab_mge_Inf_6M_iap$V2 = factor(as.character(sigtab_mge_Inf_6M_iap$V2), levels = names(x))
# Gene order
x = tapply(sigtab_mge_Inf_6M_iap$log2FoldChange, sigtab_mge_Inf_6M_iap$V3, function(x) max(x))
x = sort(x, TRUE)
sigtab_mge_Inf_6M_iap$V3 = factor(as.character(sigtab_mge_Inf_6M_iap$V3), levels = names(x))


# Plot
f <- ggplot(sigtab_mge_Inf_6M_iap, aes(x = V3, y = log2FoldChange, color = V2, 
    size = n)) + geom_point(alpha = 0.9) + theme(axis.text.x = element_text(angle = 90, 
    hjust = 0.95, vjust = 0.5, size = rel(0.7)), axis.text.y = element_text(size = rel(0.7)), 
    axis.title.x = element_blank(), axis.title.y = element_text(size = rel(0.6)), 
    legend.text = element_text(size = rel(0.55)), legend.title = element_text(size = rel(0.6)), 
    legend.position = c(0.95, 0.8), legend.key.width = unit(0.1, "lines"), legend.key.height = unit(0.5, 
        "lines")) + scale_color_brewer(palette = "Set3") + scale_size(range = c(1, 
    5), breaks = c(1, 2, 3, 4, 5, 6, 7, 8, 9, 10)) + labs(color = "") + guides(size = FALSE) + 
    ylim(-10, 10) + geom_hline(yintercept = 0, linetype = 2, color = "grey", 
    alpha = 0.7) + ylab("log2 fold change\ncontrol          IAP") + ggtitle("IAP, six-month-old infants")


# Cowplot
plot_grid(a, b, c, d, e, f, ncol = 2, align = "h", labels = "auto")
```

# Venn diagrams

```
#### Venn diagrams of shared species between mothers and infants###

# Metaphlan mothers and infants
PHY_SP_Inf_1M_temp <- otu_table(subset_samples(PHY_SP, TYPE %in% c("Inf_1M", 
    "Inf_6M")))[rowSums(otu_table(subset_samples(PHY_SP, TYPE %in% c("Inf_1M", 
    "Inf_6M")))) > 0]
nrow(PHY_SP_Inf_1M_temp)
```

```
## [1] 151
```

```
PHY_SP_Mot_1M_temp <- otu_table(subset_samples(PHY_SP, TYPE %in% c("Mot_1M", 
    "Mot_32W")))[rowSums(otu_table(subset_samples(PHY_SP, TYPE %in% c("Mot_1M", 
    "Mot_32W")))) > 0]

length(intersect(row.names(PHY_SP_Mot_1M_temp), (row.names(PHY_SP_Inf_1M_temp))))
```

```
## [1] 97
```

```
# Draw Venn
grid.newpage()
draw.pairwise.venn(nrow(PHY_SP_Inf_1M_temp), nrow(PHY_SP_Mot_1M_temp), length(intersect(row.names(PHY_SP_Mot_1M_temp), 
    (row.names(PHY_SP_Inf_1M_temp)))), category = c("Infants", "Mothers"), lty = rep("blank", 
    2), fill = c("forestgreen", "dodgerblue4"), fontfamily = "sans", cat.fontfamily = "sans", 
    alpha = rep(0.5, 2), cat.pos = c(0, 0), cat.dist = rep(0.025, 2))
```

```
## (polygon[GRID.polygon.1703], polygon[GRID.polygon.1704], polygon[GRID.polygon.1705], polygon[GRID.polygon.1706], text[GRID.text.1707], text[GRID.text.1708], text[GRID.text.1709], text[GRID.text.1710], text[GRID.text.1711])
```

```
# Metaphlan breastmilk and infants

PHY_SP_Milk_CL_temp <- otu_table(subset_samples(PHY_SP, TYPE %in% c("Milk_CL", 
    "Milk_1M")))[rowSums(otu_table(subset_samples(PHY_SP, TYPE %in% c("Milk_CL", 
    "Milk_1M")))) > 0]

length(intersect(row.names(PHY_SP_Milk_CL_temp), (row.names(PHY_SP_Inf_1M_temp))))
```

```
## [1] 26
```

```
grid.newpage()
draw.pairwise.venn(nrow(PHY_SP_Inf_1M_temp), nrow(PHY_SP_Milk_CL_temp), length(intersect(row.names(PHY_SP_Milk_CL_temp), 
    (row.names(PHY_SP_Inf_1M_temp)))), category = c("Infants", "Breastmilk"), 
    lty = rep("blank", 2), fill = c("forestgreen", "red"), fontfamily = "sans", 
    cat.fontfamily = "sans", alpha = rep(0.5, 2), cat.pos = c(0, 0), cat.dist = rep(0.025, 
        2))
```

```
## (polygon[GRID.polygon.1712], polygon[GRID.polygon.1713], polygon[GRID.polygon.1714], polygon[GRID.polygon.1715], text[GRID.text.1716], text[GRID.text.1717], text[GRID.text.1718], text[GRID.text.1719], text[GRID.text.1720])
```

```
# Metaphlan mothers and breastmilk

grid.newpage()
draw.pairwise.venn(nrow(PHY_SP_Mot_1M_temp), nrow(PHY_SP_Milk_CL_temp), length(intersect(row.names(PHY_SP_Milk_CL_temp), 
    (row.names(PHY_SP_Mot_1M_temp)))), category = c("Mothers", "Breastmilk"), 
    lty = rep("blank", 2), fill = c("dodgerblue4", "red"), fontfamily = "sans", 
    cat.fontfamily = "sans", alpha = rep(0.5, 2), cat.pos = c(0, 0), cat.dist = rep(0.025, 
        2))
```

```
## (polygon[GRID.polygon.1721], polygon[GRID.polygon.1722], polygon[GRID.polygon.1723], polygon[GRID.polygon.1724], text[GRID.text.1725], text[GRID.text.1726], text[GRID.text.1727], text[GRID.text.1728], text[GRID.text.1729])
```

```
# ARGs Mothers and infants

PHY_ARG_Inf_1M_temp <- otu_table(subset_samples(ARG_PHY, TYPE %in% c("Inf_1M", 
    "Inf_6M")))[rowSums(otu_table(subset_samples(ARG_PHY, TYPE %in% c("Inf_1M", 
    "Inf_6M")))) > 0]

PHY_ARG_Mot_1M_temp <- otu_table(subset_samples(ARG_PHY, TYPE %in% c("Mot_32W", 
    "Mot_1M")))[rowSums(otu_table(subset_samples(ARG_PHY, TYPE %in% c("Mot_32W", 
    "Mot_1M")))) > 0]

length(intersect(row.names(PHY_ARG_Mot_1M_temp), (row.names(PHY_ARG_Inf_1M_temp))))
```

```
## [1] 264
```

```
grid.newpage()
draw.pairwise.venn(nrow(PHY_ARG_Inf_1M_temp), nrow(PHY_ARG_Mot_1M_temp), length(intersect(row.names(PHY_ARG_Mot_1M_temp), 
    (row.names(PHY_ARG_Inf_1M_temp)))), fontfamily = "sans", cat.fontfamily = "sans", 
    category = c("Infants", "Mothers"), lty = rep("blank", 2), fill = c("forestgreen", 
        "dodgerblue4"), alpha = rep(0.5, 2), cat.pos = c(0, 0), cat.dist = rep(0.025, 
        2))
```

```
## (polygon[GRID.polygon.1730], polygon[GRID.polygon.1731], polygon[GRID.polygon.1732], polygon[GRID.polygon.1733], text[GRID.text.1734], text[GRID.text.1735], text[GRID.text.1736], text[GRID.text.1737], text[GRID.text.1738])
```

```
# Breastmilk and infants

PHY_ARG_Milk_CL_temp <- otu_table(subset_samples(ARG_PHY, TYPE %in% c("Milk_CL", 
    "Milk_CL")))[rowSums(otu_table(subset_samples(ARG_PHY, TYPE %in% c("Milk_CL", 
    "Milk_1M")))) > 0]

length(intersect(row.names(PHY_ARG_Milk_CL_temp), (row.names(PHY_ARG_Inf_1M_temp))))
```

```
## [1] 68
```

```
grid.newpage()
draw.pairwise.venn(nrow(PHY_ARG_Inf_1M_temp), nrow(PHY_ARG_Milk_CL_temp), length(intersect(row.names(PHY_ARG_Milk_CL_temp), 
    (row.names(PHY_ARG_Inf_1M_temp)))), fontfamily = "sans", cat.fontfamily = "sans", 
    category = c("Infants", "Breastmilk"), lty = rep("blank", 2), fill = c("forestgreen", 
        "red"), alpha = rep(0.5, 2), cat.pos = c(0, 0), cat.dist = rep(0.025, 
        2))
```

```
## (polygon[GRID.polygon.1739], polygon[GRID.polygon.1740], polygon[GRID.polygon.1741], polygon[GRID.polygon.1742], text[GRID.text.1743], text[GRID.text.1744], text[GRID.text.1745], text[GRID.text.1746], text[GRID.text.1747])
```

```
# Mothers and breastmilk
grid.newpage()
draw.pairwise.venn(nrow(PHY_ARG_Mot_1M_temp), nrow(PHY_ARG_Milk_CL_temp), length(intersect(row.names(PHY_ARG_Milk_CL_temp), 
    (row.names(PHY_ARG_Mot_1M_temp)))), fontfamily = "sans", cat.fontfamily = "sans", 
    category = c("Mothers", "Breastmilk"), lty = rep("blank", 2), fill = c("dodgerblue4", 
        "red"), alpha = rep(0.5, 2), cat.pos = c(0, 0), cat.dist = rep(0.025, 
        2))
```

```
## (polygon[GRID.polygon.1748], polygon[GRID.polygon.1749], polygon[GRID.polygon.1750], polygon[GRID.polygon.1751], text[GRID.text.1752], text[GRID.text.1753], text[GRID.text.1754], text[GRID.text.1755], text[GRID.text.1756])
```

```
########## MGEs###########

# Mothers and infants

PHY_MGE_Inf_1M_temp <- otu_table(subset_samples(MGE_PHY, TYPE %in% c("Inf_1M", 
    "Inf_6M")))[rowSums(otu_table(subset_samples(MGE_PHY, TYPE %in% c("Inf_1M", 
    "Inf_6M")))) > 0]

PHY_MGE_Mot_1M_temp <- otu_table(subset_samples(MGE_PHY, TYPE %in% c("Mot_32W", 
    "Mot_1M")))[rowSums(otu_table(subset_samples(MGE_PHY, TYPE %in% c("Mot_32W", 
    "Mot_1M")))) > 0]

length(intersect(row.names(PHY_MGE_Mot_1M_temp), (row.names(PHY_MGE_Inf_1M_temp))))
```

```
## [1] 287
```

```
grid.newpage()
draw.pairwise.venn(nrow(PHY_MGE_Inf_1M_temp), nrow(PHY_MGE_Mot_1M_temp), length(intersect(row.names(PHY_MGE_Mot_1M_temp), 
    (row.names(PHY_MGE_Inf_1M_temp)))), fontfamily = "sans", cat.fontfamily = "sans", 
    category = c("Infants", "Mothers"), lty = rep("blank", 2), fill = c("forestgreen", 
        "dodgerblue4"), alpha = rep(0.5, 2), cat.pos = c(0, 0), cat.dist = rep(0.025, 
        2))
```

```
## (polygon[GRID.polygon.1757], polygon[GRID.polygon.1758], polygon[GRID.polygon.1759], polygon[GRID.polygon.1760], text[GRID.text.1761], text[GRID.text.1762], text[GRID.text.1763], text[GRID.text.1764], text[GRID.text.1765])
```

```
# Breastmilk and infants

PHY_MGE_Milk_CL_temp <- otu_table(subset_samples(MGE_PHY, TYPE %in% c("Milk_CL", 
    "Milk_CL")))[rowSums(otu_table(subset_samples(MGE_PHY, TYPE %in% c("Milk_CL", 
    "Milk_1M")))) > 0]

grid.newpage()
draw.pairwise.venn(nrow(PHY_MGE_Inf_1M_temp), nrow(PHY_MGE_Milk_CL_temp), length(intersect(row.names(PHY_MGE_Milk_CL_temp), 
    (row.names(PHY_MGE_Inf_1M_temp)))), fontfamily = "sans", cat.fontfamily = "sans", 
    category = c("Infants", "Breastmilk"), lty = rep("blank", 2), fill = c("forestgreen", 
        "red"), alpha = rep(0.5, 2), cat.pos = c(0, 0), cat.dist = rep(0.025, 
        2))
```

```
## (polygon[GRID.polygon.1766], polygon[GRID.polygon.1767], polygon[GRID.polygon.1768], polygon[GRID.polygon.1769], text[GRID.text.1770], text[GRID.text.1771], text[GRID.text.1772], text[GRID.text.1773], text[GRID.text.1774])
```

```
# Mothers and breastmilk
grid.newpage()
draw.pairwise.venn(nrow(PHY_MGE_Mot_1M_temp), nrow(PHY_MGE_Milk_CL_temp), length(intersect(row.names(PHY_MGE_Milk_CL_temp), 
    (row.names(PHY_MGE_Mot_1M_temp)))), fontfamily = "sans", cat.fontfamily = "sans", 
    category = c("Mothers", "Breastmilk"), lty = rep("blank", 2), fill = c("dodgerblue4", 
        "red"), alpha = rep(0.5, 2), cat.pos = c(0, 0), cat.dist = rep(0.025, 
        2))
```

```
## (polygon[GRID.polygon.1775], polygon[GRID.polygon.1776], polygon[GRID.polygon.1777], polygon[GRID.polygon.1778], text[GRID.text.1779], text[GRID.text.1780], text[GRID.text.1781], text[GRID.text.1782], text[GRID.text.1783])
```

```
grid.newpage()
draw.triple.venn(area1 = nrow(PHY_ARG_Inf_1M_temp), area2 = nrow(PHY_ARG_Milk_CL_temp), 
    area3 = nrow(PHY_ARG_Mot_1M_temp), n12 = length(intersect(row.names(PHY_ARG_Milk_CL_temp), 
        (row.names(PHY_ARG_Inf_1M_temp)))), n23 = length(intersect(row.names(PHY_ARG_Mot_1M_temp), 
        (row.names(PHY_ARG_Milk_CL_temp)))), n13 = length(intersect(row.names(PHY_ARG_Inf_1M_temp), 
        (row.names(PHY_ARG_Mot_1M_temp)))), n123 = length(intersect(intersect(row.names(PHY_ARG_Mot_1M_temp), 
        (row.names(PHY_ARG_Inf_1M_temp))), row.names(PHY_ARG_Milk_CL_temp))), 
    fontfamily = "sans", cat.fontfamily = "sans", category = c("Infants", "Breastmilk", 
        "Mothers"), lty = rep("blank", 3), fill = c("forestgreen", "red", "dodgerblue4"), 
    alpha = rep(0.5, 3))
```

```
## (polygon[GRID.polygon.1784], polygon[GRID.polygon.1785], polygon[GRID.polygon.1786], polygon[GRID.polygon.1787], polygon[GRID.polygon.1788], polygon[GRID.polygon.1789], text[GRID.text.1790], text[GRID.text.1791], text[GRID.text.1792], text[GRID.text.1793], text[GRID.text.1794], text[GRID.text.1795], text[GRID.text.1796], text[GRID.text.1797], text[GRID.text.1798], text[GRID.text.1799])
```

# Checking similarity between mothers and infants from the same family using vegdist Horn-Morisita and density plots

This is done on relative abundance data, but for the manuscript presence absence data was used. Similar comparisons were done for Jaccard similarity matrices produced by Sourmash and for effects of the sampling time and IAP on similarity. Similar comparisons were also done for breast milk.

```
# Similarity between mothers and children using vegdist

# Read in function for testing significances
lmp = function(y, x, n.perms = 9999) {
    test = numeric(n.perms)
    test[1] = abs(summary(lm(y ~ x))$coefficients[2, 3])
    for (ii in 2:n.perms) {
        test[ii] = abs(summary(lm(sample(y) ~ x))$coefficients[2, 3])
    }
    p.value = sum(test >= test[1])/n.perms
    return(p.value)
}

# Species Comparing mothers and their own children make a distance matrix
# for samples
PHY_SP_AUInf_1M <- subset_samples(PHY_SP, TYPE2 %in% c("MOT", "INF"))
otu <- sqrt(t(otu_table(PHY_SP_AUInf_1M)))
y <- vegdist(otu, upper = TRUE, diag = TRUE, method = "horn")
distance <- as.vector(y)

# make a matrix for family aka PAIR
x = matrix(NA, 64, 64)
Fvec <- sample_data(PHY_SP_AUInf_1M)$PAIR
for (n in 1:(ncol(x) - 1)) {
    for (m in (n + 1):ncol(x)) {
        f <- eval(Fvec[n] == Fvec[m])
        x[n, m] = f
    }
}
x <- t(x)
family_vec <- x[lower.tri(x, diag = F)]

# and the same for TYPE
x = matrix(NA, 64, 64)
Yvec <- sample_data(PHY_SP_AUInf_1M)$TYPE2
for (n in 1:(ncol(x) - 1)) {
    for (m in (n + 1):ncol(x)) {
        f <- eval(Yvec[n] == Yvec[m])
        x[n, m] = f
    }
}
x <- t(x)
type_vec <- x[lower.tri(x, diag = F)]

# First is for family and second is for type.
zz <- data.frame(distance, family_vec, type_vec)
zz$comb <- paste(zz$family_vec, zz$type_vec, sep = "-")


# Density plot
a1 <- ggplot(zz, aes(x = distance, fill = factor(comb))) + geom_density(alpha = 0.5) + 
    theme(legend.justification = c(0.05, 1), legend.position = c(0.05, 1), legend.text = element_text(size = rel(0.8))) + 
    scale_fill_manual(values = c("#999999", "#E69F00", "#56B4E9"), labels = c("Different family, mother and infant", 
        "Differen family, same type", "Same family, mother and infant"), "Species") + 
    annotate("text", x = 0.65, y = 2.2, label = "type, p=0.0001") + xlab("Between-sample dissimilarity") + 
    ggtitle("Mothers and infants") + ylim(0, 8)


# Remove the FALSE-TRUE, or different family same type
zz_temp <- zz[grep("FALSE-TRUE", zz$comb, invert = TRUE), ]

# Test significance
lmp(zz_temp$distance, zz_temp$comb)
```

```
## [1] 0.01760176
```

```
# Remove the FALSE-FALSE, or different family different type
zz_temp <- zz[grep("FALSE-FALSE", zz$comb, invert = TRUE), ]

# Test significance
lmp(zz_temp$distance, zz_temp$comb)
```

```
## [1] 0.00010001
```

```
# Remove TRUE-FALSE, or same family different type, this is significant
# p=0.0001, dif. fam dif type vs. dif fam same type
zz_temp <- zz[grep("TRUE-FALSE", zz$comb, invert = TRUE), ]

# Test significance
lmp(zz_temp$distance, zz_temp$comb)
```

```
## [1] 0.00010001
```

```
# Species Comparing infants to their own samples at two time points make a
# distance matrix for samples
PHY_SP_Inf_6MInf_1M <- subset_samples(PHY_SP, TYPE %in% c("Inf_1M", "Inf_6M"))
otu <- t(otu_table(PHY_SP_Inf_6MInf_1M))
y <- vegdist(otu, upper = TRUE, diag = TRUE, method = "horn")
distance <- as.vector(y)

# make a matrix for family aka PAIR
x = matrix(NA, 32, 32)
Fvec <- sample_data(PHY_SP_Inf_6MInf_1M)$PAIR
for (n in 1:(ncol(x) - 1)) {
    for (m in (n + 1):ncol(x)) {
        f <- eval(Fvec[n] == Fvec[m])
        x[n, m] = f
    }
}
x <- t(x)
family_vec <- x[lower.tri(x, diag = F)]

# and the same for TYPE
x = matrix(NA, 32, 32)
Yvec <- sample_data(PHY_SP_Inf_6MInf_1M)$TYPE
for (n in 1:(ncol(x) - 1)) {
    for (m in (n + 1):ncol(x)) {
        f <- eval(Yvec[n] == Yvec[m])
        x[n, m] = f
    }
}
x <- t(x)
type_vec <- x[lower.tri(x, diag = F)]

# First is for family and second is for type.
zz <- data.frame(distance, family_vec, type_vec)
zz$comb <- paste(zz$family_vec, zz$type_vec, sep = "-")


# Density plot

b1 <- ggplot(zz, aes(x = distance, fill = factor(comb))) + geom_density(alpha = 0.5) + 
    theme(legend.justification = c(0.05, 1), legend.position = c(0.05, 1), legend.text = element_text(size = rel(0.8))) + 
    scale_fill_manual(values = c("#999999", "#E69F00", "#56B4E9"), labels = c("Different infant, different age", 
        "Different infant, same age", "Same infant, different age"), "Species") + 
    annotate("text", x = 0.45, y = 2.5, label = "") + xlab("Between-sample dissimilarity") + 
    ggtitle("One- and six-month-old infants") + ylim(0, 8)


# Remove the FALSE-TRUE, or different family same type, this is weakly
# signifcant with Bray p=0.07, individual is significant
zz_temp <- zz[grep("FALSE-TRUE", zz$comb, invert = TRUE), ]

# Test significance
lmp(zz_temp$distance, zz_temp$comb)
```

```
## [1] 0.1741174
```

```
# Remove the FALSE-FALSE, or different family different type, this is weakly
# significant p=0.09, dif fam same age vs. same-infant different age
zz_temp <- zz[grep("FALSE-FALSE", zz$comb, invert = TRUE), ]

# Test significance
lmp(zz_temp$distance, zz_temp$comb)
```

```
## [1] 0.08960896
```

```
# Remove TRUE-FALSE, or same family different type
zz_temp <- zz[grep("TRUE-FALSE", zz$comb, invert = TRUE), ]

# Test significance
lmp(zz_temp$distance, zz_temp$comb)
```

```
## [1] 0.4425443
```

```
# ARGs

# Comparing mothers and their own children make a distance matrix for
# samples
PHY_ARG_AUInf_1M <- subset_samples(ARG_PHY, TYPE2 %in% c("MOT", "INF"))
otu <- (t(otu_table(PHY_ARG_AUInf_1M)))
y <- vegdist(otu, upper = TRUE, diag = TRUE, method = "horn")
distance <- as.vector(y)

# make a matrix for family aka PAIR
x = matrix(NA, 64, 64)
Fvec <- sample_data(PHY_ARG_AUInf_1M)$PAIR
for (n in 1:(ncol(x) - 1)) {
    for (m in (n + 1):ncol(x)) {
        f <- eval(Fvec[n] == Fvec[m])
        x[n, m] = f
    }
}
x <- t(x)
family_vec <- x[lower.tri(x, diag = F)]

# and the same for TYPE
x = matrix(NA, 64, 64)
Yvec <- sample_data(PHY_ARG_AUInf_1M)$TYPE2
for (n in 1:(ncol(x) - 1)) {
    for (m in (n + 1):ncol(x)) {
        f <- eval(Yvec[n] == Yvec[m])
        x[n, m] = f
    }
}
x <- t(x)
type_vec <- x[lower.tri(x, diag = F)]

# First is for family and second is for type.
zz <- data.frame(distance, family_vec, type_vec)
zz$comb <- paste(zz$family_vec, zz$type_vec, sep = "-")

# Density plot
c <- ggplot(zz, aes(x = distance, fill = factor(comb))) + geom_density(alpha = 0.5) + 
    theme(legend.justification = c(0.05, 1), legend.position = c(0.05, 1), legend.text = element_text(size = rel(0.8))) + 
    scale_fill_manual(values = c("#999999", "#E69F00", "#56B4E9"), labels = c("Different family, mother and infant", 
        "Differen family, same type", "Same family, mother and infant"), "ARGs") + 
    annotate("text", x = 0.7, y = 3, label = "type, p=0.0001    \nsame type vs. same family, p=0.02                                    ") + 
    xlab("Between-sample dissimilarity") + ggtitle("Mothers and infants") + 
    ylim(0, 8)

# Remove the FALSE-TRUE, or different family same type
zz_temp <- zz[grep("FALSE-TRUE", zz$comb, invert = TRUE), ]

# Test significance, this is weakly significant p=0.09 dif fam dif type vs.
# same fam dif type
lmp(zz_temp$distance, zz_temp$comb)
```

```
## [1] 0.3228323
```

```
# Remove the FALSE-FALSE, or different family different type
zz_temp <- zz[grep("FALSE-FALSE", zz$comb, invert = TRUE), ]
boxplot(zz_temp$distance ~ zz_temp$comb)
```

```
# Test significance, this is significant p=0.02, own mother vs. other child,
# other child is more similar than own mother
lmp(zz_temp$distance, zz_temp$comb)
```

```
## [1] 0.00010001
```

```
# Remove TRUE-FALSE, or same family different type, this is significant
# dif-fam-dif-type vs. dif-fam-same-type TYPE p 0.0001
zz_temp <- zz[grep("TRUE-FALSE", zz$comb, invert = TRUE), ]

# Test significance
lmp(zz_temp$distance, zz_temp$comb)
```

```
## [1] 0.00010001
```

```
# ARGs Comparing infants to themselves make a distance matrix for samples
PHY_ARG_Inf_6MInf_1M <- subset_samples(ARG_PHY, TYPE %in% c("Inf_6M", "Inf_1M"))
otu <- t(otu_table(PHY_ARG_Inf_6MInf_1M))
y <- vegdist(otu, upper = TRUE, diag = TRUE, method = "horn")
distance <- as.vector(y)

# make a matrix for family aka PAIR
x = matrix(NA, 32, 32)
Fvec <- sample_data(PHY_ARG_Inf_6MInf_1M)$PAIR
for (n in 1:(ncol(x) - 1)) {
    for (m in (n + 1):ncol(x)) {
        f <- eval(Fvec[n] == Fvec[m])
        x[n, m] = f
    }
}
x <- t(x)
family_vec <- x[lower.tri(x, diag = F)]

# and the same for TYPE
x = matrix(NA, 32, 32)
Yvec <- sample_data(PHY_ARG_Inf_6MInf_1M)$TYPE
for (n in 1:(ncol(x) - 1)) {
    for (m in (n + 1):ncol(x)) {
        f <- eval(Yvec[n] == Yvec[m])
        x[n, m] = f
    }
}
x <- t(x)
type_vec <- x[lower.tri(x, diag = F)]

# First is for family and second is for type.
zz <- data.frame(distance, family_vec, type_vec)
zz$comb <- paste(zz$family_vec, zz$type_vec, sep = "-")


# Density plot
d <- ggplot(zz, aes(x = distance, fill = factor(comb))) + geom_density(alpha = 0.5) + 
    theme(legend.justification = c(0.05, 1), legend.position = c(0.05, 1), legend.text = element_text(size = rel(0.8))) + 
    scale_fill_manual(values = c("#999999", "#E69F00", "#56B4E9"), labels = c("Different infant, different age", 
        "Different infant, same age", "Same infant, different age"), "ARGs") + 
    xlab("Between-sample dissimilarity") + ggtitle("One- and six-month-old infants") + 
    ylim(0, 8)

# Remove the FALSE-TRUE, or different family same type, this is weakly
# significant with Bray p=0.08, Individual is significant with Bray
zz_temp <- zz[grep("FALSE-TRUE", zz$comb, invert = TRUE), ]

# Test significance
lmp(zz_temp$distance, zz_temp$comb)
```

```
## [1] 0.1843184
```

```
# Remove the FALSE-FALSE, or different family different type
zz_temp <- zz[grep("FALSE-FALSE", zz$comb, invert = TRUE), ]

# Test significance
lmp(zz_temp$distance, zz_temp$comb)
```

```
## [1] 0.1631163
```

```
# Remove TRUE-FALSE, or same family different type
zz_temp <- zz[grep("TRUE-FALSE", zz$comb, invert = TRUE), ]

# Test significance
lmp(zz_temp$distance, zz_temp$comb)
```

```
## [1] 0.9120912
```

```
# MGEs Comparing mothers and their own children make a distance matrix for
# samples
PHY_MGE_AUInf_1M <- subset_samples(MGE_PHY, TYPE2 %in% c("MOT", "INF"))
otu <- t(otu_table(PHY_MGE_AUInf_1M))
y <- vegdist(otu, upper = TRUE, diag = TRUE, method = "bray")
distance <- as.vector(y)

# make a matrix for family aka PAIR
x = matrix(NA, 64, 64)
Fvec <- sample_data(PHY_MGE_AUInf_1M)$PAIR
for (n in 1:(ncol(x) - 1)) {
    for (m in (n + 1):ncol(x)) {
        f <- eval(Fvec[n] == Fvec[m])
        x[n, m] = f
    }
}
x <- t(x)
family_vec <- x[lower.tri(x, diag = F)]

# and the same for TYPE
x = matrix(NA, 64, 64)
Yvec <- sample_data(PHY_MGE_AUInf_1M)$TYPE2
for (n in 1:(ncol(x) - 1)) {
    for (m in (n + 1):ncol(x)) {
        f <- eval(Yvec[n] == Yvec[m])
        x[n, m] = f
    }
}
x <- t(x)
type_vec <- x[lower.tri(x, diag = F)]

# First is for family and second is for type.
zz <- data.frame(distance, family_vec, type_vec)
zz$comb <- paste(zz$family_vec, zz$type_vec, sep = "-")

# Density plot
e <- ggplot(zz, aes(x = distance, fill = factor(comb))) + geom_density(alpha = 0.5) + 
    theme(legend.justification = c(0.05, 1), legend.position = c(0.05, 1), legend.text = element_text(size = rel(0.8))) + 
    scale_fill_manual(values = c("#999999", "#E69F00", "#56B4E9"), labels = c("Different family, mother and infant", 
        "Differen family, same type", "Same family, mother and infant"), "MGEs") + 
    annotate("text", x = 0.75, y = 3.5, label = "family, p=0.03\ntype, p=0.0001") + 
    xlab("Between-sample dissimilarity") + ggtitle("Mothers and infants") + 
    ylim(0, 8)


# Remove the FALSE-TRUE, or different family same type
zz_temp <- zz[grep("FALSE-TRUE", zz$comb, invert = TRUE), ]

# Test significance, this is significant dif fam dif type vs. same fam dif
# type p=0.0259, this is NOT significant with BRAY
lmp(zz_temp$distance, zz_temp$comb)
```

```
## [1] 0.00980098
```

```
# Remove the FALSE-FALSE, or different family different type, this is
# significant with BRAY 0.04, or TYPE over family
zz_temp <- zz[grep("FALSE-FALSE", zz$comb, invert = TRUE), ]

# Test significance
lmp(zz_temp$distance, zz_temp$comb)
```

```
## [1] 0.01270127
```

```
# Remove TRUE-FALSE, or same family different type
zz_temp <- zz[grep("TRUE-FALSE", zz$comb, invert = TRUE), ]

# Test significance, this is significant p=0.0001, dif fam dif type vc dif
# fam same type
lmp(zz_temp$distance, zz_temp$comb)
```

```
## [1] 0.00010001
```

```
# Comparing infants to themselves

# make a distance matrix for samples
PHY_MGE_Inf_6MInf_1M <- subset_samples(MGE_PHY, TYPE %in% c("Inf_6M", "Inf_1M"))
otu <- t(otu_table(PHY_MGE_Inf_6MInf_1M))
y <- vegdist(otu, upper = TRUE, diag = TRUE, method = "horn")
distance <- as.vector(y)

# make a matrix for family aka PAIR
x = matrix(NA, 32, 32)
Fvec <- sample_data(PHY_MGE_Inf_6MInf_1M)$PAIR
for (n in 1:(ncol(x) - 1)) {
    for (m in (n + 1):ncol(x)) {
        f <- eval(Fvec[n] == Fvec[m])
        x[n, m] = f
    }
}
x <- t(x)
family_vec <- x[lower.tri(x, diag = F)]

# and the same for TYPE
x = matrix(NA, 32, 32)
Yvec <- sample_data(PHY_MGE_Inf_6MInf_1M)$TYPE
for (n in 1:(ncol(x) - 1)) {
    for (m in (n + 1):ncol(x)) {
        f <- eval(Yvec[n] == Yvec[m])
        x[n, m] = f
    }
}
x <- t(x)
type_vec <- x[lower.tri(x, diag = F)]

# First is for family and second is for type.
zz <- data.frame(distance, family_vec, type_vec)
zz$comb <- paste(zz$family_vec, zz$type_vec, sep = "-")

# Density plot
f <- ggplot(zz, aes(x = distance, fill = factor(comb))) + geom_density(alpha = 0.5) + 
    theme(legend.justification = c(0.05, 1), legend.position = c(0.05, 1), legend.text = element_text(size = rel(0.8))) + 
    scale_fill_manual(values = c("#999999", "#E69F00", "#56B4E9"), labels = c("Different infant, different age", 
        "Different infant, same age", "Same infant, different age"), "MGEs") + 
    annotate("text", x = 0.55, y = 2.5, label = "individual, p=0.0008\nsame infant vs. same age, p=0.0015                         ") + 
    xlab("Between-sample dissimilarity") + ylim(0, 8) + ggtitle("One- and six-month-old infants")

# Remove the FALSE-TRUE, or different family same type
zz_temp <- zz[grep("FALSE-TRUE", zz$comb, invert = TRUE), ]

# Test significance, this is significant dif fam dif type vs. same fam dif
# type p=0.0008
lmp(zz_temp$distance, zz_temp$comb)
```

```
## [1] 0.00020002
```

```
# Remove the FALSE-FALSE, or different family different type
zz_temp <- zz[grep("FALSE-FALSE", zz$comb, invert = TRUE), ]

# Test significance, this is significant p=0.001, dif in same type vs. same
# in dif type, infant more similar to himself than to an other infant of the
# same age
lmp(zz_temp$distance, zz_temp$comb)
```

```
## [1] 0.00140014
```

```
# Remove TRUE-FALSE, or same family different type
zz_temp <- zz[grep("TRUE-FALSE", zz$comb, invert = TRUE), ]

# Test significance
lmp(zz_temp$distance, zz_temp$comb)
```

```
## [1] 0.7324732
```

# Next we move onto analyzing the changes in different taxa between different sample types or treatments using DeSEQ

Even though the whole community looks similar with these sample sizes, there might be some taxa that change significantly.

## Check which genera are different between the 1 month and 6 month old infants using DeSEQ and mothers and infants

```
############## DESEQ################ 1 month old vs. 6 month old infants
temp <- metaphlan_sp[, ] * 10^5 + 1
MTX_DSQ <- phyloseq(otu_table(temp, taxa_are_rows = T), sample_data(sample_data), 
    tax_table(as.matrix(tax_sp)))

# Glom to Genus taxonomic level
MTX_DSQ <- tax_glom(MTX_DSQ, taxrank = "V7")

MTX_DSQ_LU <- subset_samples(MTX_DSQ, (TYPE == "Inf_1M" | TYPE == "Inf_6M"))
dds_mtx = phyloseq_to_deseq2(MTX_DSQ_LU, ~TYPE)
```

```
## converting counts to integer mode
```

```
dds_mtx = DESeq(dds_mtx, fitType = "mean", test = "Wald", betaPrior = FALSE)
```

```
## estimating size factors
```

```
## estimating dispersions
```

```
## gene-wise dispersion estimates
```

```
## mean-dispersion relationship
```

```
## final dispersion estimates
```

```
## fitting model and testing
```

```
## -- replacing outliers and refitting for 58 genes
## -- DESeq argument 'minReplicatesForReplace' = 7 
## -- original counts are preserved in counts(dds)
```

```
## estimating dispersions
```

```
## fitting model and testing
```

```
res_mtx = results(dds_mtx, cooksCutoff = FALSE)
alpha = 0.05
sigtab_mtx = res_mtx[which(res_mtx$padj < alpha), ]

sigtab_mtxLU = cbind(as(sigtab_mtx, "data.frame"), as(tax_table(MTX_DSQ_LU)[rownames(sigtab_mtx), 
    ], "matrix"))

kable(sigtab_mtxLU, caption = "genera different in 1 month at 6 month old infants (neg higher in 1 month olds)")
```

genera different in 1 month at 6 month old infants (neg higher in 1 month olds)

|  | baseMean | log2FoldChange | lfcSE | stat | pvalue | padj | V2 | V3 | V4 | V5 | V6 | V7 | V8 |
| --- | --- | --- | --- | --- | --- | --- | --- | --- | --- | --- | --- | --- | --- |
| OTU15 | 5.499066e+01 | 6.612734 | 1.0676060 | 6.193983 | 0.0000000 | 0.0000000 | Bacteria | Actinobacteria | Actinobacteria | Actinomycetales | Actinomycetaceae | Varibaculum | NA |
| OTU17 | 4.783429e+02 | -7.820859 | 0.8565197 | -9.130975 | 0.0000000 | 0.0000000 | Bacteria | Actinobacteria | Actinobacteria | Actinomycetales | Corynebacteriaceae | Corynebacterium | NA |
| OTU23 | 4.867661e+03 | -8.873296 | 1.0658900 | -8.324776 | 0.0000000 | 0.0000000 | Bacteria | Actinobacteria | Actinobacteria | Actinomycetales | Propionibacteriaceae | Propionibacterium | NA |
| OTU100 | 1.717973e+04 | -11.269756 | 1.1201310 | -10.061106 | 0.0000000 | 0.0000000 | Bacteria | Firmicutes | Bacilli | Bacillales | Staphylococcaceae | Staphylococcus | NA |
| OTU105 | 7.662201e+00 | 2.559405 | 0.7665074 | 3.339048 | 0.0008407 | 0.0023392 | Bacteria | Firmicutes | Bacilli | Lactobacillales | Carnobacteriaceae | Granulicatella | NA |
| OTU136 | 6.609206e+04 | -3.370768 | 0.8368547 | -4.027901 | 0.0000563 | 0.0001637 | Bacteria | Firmicutes | Bacilli | Lactobacillales | Streptococcaceae | Streptococcus | NA |
| OTU150 | 1.116592e+05 | 13.179275 | 1.1474472 | 11.485736 | 0.0000000 | 0.0000000 | Bacteria | Firmicutes | Clostridia | Clostridiales | Clostridiaceae | Clostridium | NA |
| OTU159 | 6.354018e+01 | -5.316918 | 1.1262997 | -4.720696 | 0.0000024 | 0.0000075 | Bacteria | Firmicutes | Clostridia | Clostridiales | Clostridiales\_Family\_XI\_Incertae\_Sedis | Finegoldia | NA |
| OTU162 | 6.143498e+02 | 9.104779 | 1.1315437 | 8.046334 | 0.0000000 | 0.0000000 | Bacteria | Firmicutes | Clostridia | Clostridiales | Clostridiales\_noname | Clostridiales\_noname | NA |
| OTU163 | 1.772787e+01 | 4.946569 | 0.9821537 | 5.036451 | 0.0000005 | 0.0000016 | Bacteria | Firmicutes | Clostridia | Clostridiales | Clostridiales\_noname | Flavonifractor | NA |
| OTU167 | 1.137249e+04 | 11.316069 | 1.1085168 | 10.208298 | 0.0000000 | 0.0000000 | Bacteria | Firmicutes | Clostridia | Clostridiales | Eubacteriaceae | Eubacterium | NA |
| OTU175 | 1.483157e+02 | 6.455572 | 0.9869245 | 6.541100 | 0.0000000 | 0.0000000 | Bacteria | Firmicutes | Clostridia | Clostridiales | Lachnospiraceae | Anaerostipes | NA |
| OTU179 | 9.894210e+04 | 8.201315 | 1.2744056 | 6.435404 | 0.0000000 | 0.0000000 | Bacteria | Firmicutes | Clostridia | Clostridiales | Lachnospiraceae | Blautia | NA |
| OTU197 | 2.869960e+03 | 8.867645 | 0.9976193 | 8.888806 | 0.0000000 | 0.0000000 | Bacteria | Firmicutes | Clostridia | Clostridiales | Lachnospiraceae | Lachnospiraceae\_noname | NA |
| OTU206 | 3.881814e+02 | 7.436219 | 0.9947525 | 7.475447 | 0.0000000 | 0.0000000 | Bacteria | Firmicutes | Clostridia | Clostridiales | Lachnospiraceae | Roseburia | NA |
| OTU208 | 1.803337e+03 | 3.469625 | 1.1090811 | 3.128378 | 0.0017577 | 0.0046873 | Bacteria | Firmicutes | Clostridia | Clostridiales | Peptostreptococcaceae | Peptostreptococcaceae\_noname | NA |
| OTU214 | 2.067816e+01 | 5.177760 | 0.9939035 | 5.209520 | 0.0000002 | 0.0000007 | Bacteria | Firmicutes | Clostridia | Clostridiales | Ruminococcaceae | Faecalibacterium | NA |
| OTU215 | 1.036889e+01 | 1.872131 | 0.6584146 | 2.843393 | 0.0044636 | 0.0114268 | Bacteria | Firmicutes | Clostridia | Clostridiales | Ruminococcaceae | Ruminococcus | NA |
| OTU220 | 1.684698e+01 | 3.184653 | 0.7852285 | 4.055702 | 0.0000500 | 0.0001523 | Bacteria | Firmicutes | Clostridia | Clostridiales | Ruminococcaceae | Subdoligranulum | NA |
| OTU222 | 3.816362e+03 | 6.899789 | 1.2803206 | 5.389111 | 0.0000001 | 0.0000003 | Bacteria | Firmicutes | Erysipelotrichia | Erysipelotrichales | Erysipelotrichaceae | Coprobacillus | NA |
| OTU224 | 3.910083e+03 | 9.079131 | 0.9656932 | 9.401672 | 0.0000000 | 0.0000000 | Bacteria | Firmicutes | Erysipelotrichia | Erysipelotrichales | Erysipelotrichaceae | Erysipelotrichaceae\_noname | NA |
| OTU239 | 5.092094e+02 | 9.836301 | 1.1483102 | 8.565892 | 0.0000000 | 0.0000000 | Bacteria | Firmicutes | Negativicutes | Selenomonadales | Veillonellaceae | Megasphaera | NA |
| OTU258 | 4.090873e+02 | 7.963306 | 1.0286378 | 7.741603 | 0.0000000 | 0.0000000 | Bacteria | Proteobacteria | Gammaproteobacteria | Enterobacteriales | Enterobacteriaceae | Citrobacter | NA |
| OTU270 | 2.928160e+00 | 2.099608 | 0.7960758 | 2.637448 | 0.0083533 | 0.0205619 | Bacteria | Proteobacteria | Gammaproteobacteria | Enterobacteriales | Enterobacteriaceae | Raoultella | NA |
| OTU285 | 3.645941e+01 | 6.012597 | 1.0379067 | 5.793003 | 0.0000000 | 0.0000000 | Viruses | Viruses\_noname | Viruses\_noname | Caudovirales | Myoviridae | PhiCD119likevirus | NA |
| OTU291 | 1.188283e+02 | 4.202059 | 0.8220568 | 5.111641 | 0.0000003 | 0.0000011 | Viruses | Viruses\_noname | Viruses\_noname | Caudovirales | Siphoviridae | Siphoviridae\_noname | NA |

```
# Make a figure with ggplot

theme_set(theme_classic())

# Class order
x = tapply(sigtab_mtxLU$log2FoldChange, sigtab_mtxLU$V4, function(x) max(x))
x = sort(x, TRUE)
sigtab_mtxLU$V4 = factor(as.character(sigtab_mtxLU$V4), levels = names(x))
# Genus order
x = tapply(sigtab_mtxLU$log2FoldChange, sigtab_mtxLU$V7, function(x) max(x))
x = sort(x, TRUE)
sigtab_mtxLU$V7 = factor(as.character(sigtab_mtxLU$V7), levels = names(x))
ggplot(sigtab_mtxLU, aes(x = V7, y = log2FoldChange, color = V4)) + geom_point(size = 5) + 
    theme(axis.text.x = element_text(angle = -90, hjust = 0, vjust = 0.5)) + 
    scale_color_brewer(palette = "Set3", "Class") + ggtitle("Differentially abundant in 6 month vs. 1 month old infants")
```

```
# Inf_1M vs. Mot_1M Genus level
temp <- metaphlan_sp[, ] * 10^5 + 1
MTX_DSQ <- phyloseq(otu_table(temp, taxa_are_rows = T), sample_data(sample_data), 
    tax_table(as.matrix(tax_sp)))

MTX_DSQ <- tax_glom(MTX_DSQ, taxrank = "V7")

MTX_DSQ_LU <- subset_samples(MTX_DSQ, (TYPE == "Inf_1M" | TYPE == "Mot_1M"))
dds_mtx = phyloseq_to_deseq2(MTX_DSQ_LU, ~TYPE)
```

```
## converting counts to integer mode
```

```
dds_mtx = DESeq(dds_mtx, fitType = "mean", test = "Wald", betaPrior = FALSE)
```

```
## estimating size factors
```

```
## estimating dispersions
```

```
## gene-wise dispersion estimates
```

```
## mean-dispersion relationship
```

```
## final dispersion estimates
```

```
## fitting model and testing
```

```
## -- replacing outliers and refitting for 58 genes
## -- DESeq argument 'minReplicatesForReplace' = 7 
## -- original counts are preserved in counts(dds)
```

```
## estimating dispersions
```

```
## fitting model and testing
```

```
res_mtx = results(dds_mtx, cooksCutoff = FALSE)
alpha = 0.05
sigtab_mtx = res_mtx[which(res_mtx$padj < alpha), ]
sigtab_mtx = cbind(as(sigtab_mtx, "data.frame"), as(tax_table(MTX_DSQ_LU)[rownames(sigtab_mtx), 
    ], "matrix"))


# Make a figure with ggplot Class order
x = tapply(sigtab_mtx$log2FoldChange, sigtab_mtx$V4, function(x) max(x))
x = sort(x, TRUE)
sigtab_mtx$V4 = factor(as.character(sigtab_mtx$V4), levels = names(x))
# Genus order
x = tapply(sigtab_mtx$log2FoldChange, sigtab_mtx$V7, function(x) max(x))
x = sort(x, TRUE)
sigtab_mtx$V7 = factor(as.character(sigtab_mtx$V7), levels = names(x))
ggplot(sigtab_mtx, aes(x = V7, y = log2FoldChange, color = V4)) + geom_point(size = 3) + 
    theme(axis.text.x = element_text(angle = -90, hjust = 0, vjust = 0.5)) + 
    scale_color_brewer(palette = "Set3", "Class") + ggtitle("Differentially abundant in 1 month old infants vs. mothers\n1 month post partum")
```
